# Contribution of smoking to the global burden of bladder cancer from 1990 to 2021 and projections to 2046

**DOI:** 10.18332/tid/202237

**Published:** 2025-03-28

**Authors:** Kai Qi, Honghui Cheng, Yiwei Jiang, Yichun Zheng

**Affiliations:** 1 Department of Urology, The Fourth Affiliated Hospital of School of Medicine, and International School of Medicine, International Institutes of Medicine, Zhejiang University, Yiwu, People’s Republic of China

**Keywords:** global burden of disease, bladder cancer, age-standardized rate, socioeconomic, projection

## Abstract

**INTRODUCTION:**

Based on the results extracted from the Global Burden of Disease (GBD) 2021, the objective of this research is to examine the spatiotemporal trends of bladder cancer attributable to smoking from 1990 to 2021, and to make projections up to the year 2046.

**METHODS:**

This study conducted a secondary dataset analysis of smoking-attributable bladder cancer data extracted from GBD 2021. Bladder cancer was classified using the International Classification of Diseases 10th Revision (ICD-10) in GBD, and smoking exposure was defined as both current and past use of smoked tobacco products. By employing a Bayesian age-period-cohort (BAPC) model, the average annual percentage change (AAPC) was determined to examine trends over time.

**RESULTS:**

From 1990 to 2021, the number of deaths and disability-adjusted life years (DALYs) due to smoking-attributable bladder cancer increased significantly. The age-standardized death rate (ASDR) decreased, with an AAPC of -1.54 (95% CI: -1.62 – -1.46). The age-standardized DALY rate (ASDLR) also showed a decline, with an AAPC of -1.68 (95% CI: -1.81 – -1.56). The regions that experienced the most significant age-standardized rate (ASR) burden were Central Europe and Western Europe. Regions with high-medium sociodemographic index (SDI) values had the highest number of deaths and DALYs, as well as the highest ASR for both indicators. The heaviest global disease burden is concentrated among males and individuals aged ≥70 years. Smoking-attributable bladder cancer deaths are projected to rise over the next 25 years, reaching 90021.45 by 2046.

**CONCLUSIONS:**

Despite a decrease in the ASRs of smoking-attributable bladder cancer, the absolute burden has increased and is expected to continue growing. Therefore, continuous and targeted tobacco control measures and medical strategies are needed, especially for developed regions, the elderly, and male populations. And due to the unique mechanisms by which tobacco causes disease, the youth and female populations should not be neglected.

## INTRODUCTION

Bladder cancer (BCa) ranks among the leading malignancies of the urinary system and significantly contributes to global cancer mortality. Approximately 6.14 million newly diagnosed BCa cases and 2.20 million deaths globally were recorded by GLOBOCAN 2022^1^. Predictive analyses of bladder cancer also show that the rates of both incidence and mortality for BCa will maintain an upward trend in the next decade^2^. The pervasive incidence and mortality rates of bladder cancer constitute a major threat to public health worldwide.

The global tobacco epidemic remains a significant and critical challenge for the field of global health. In 2019, the number of regular smokers exceeded 1 billion, leading to nearly 8 million smoking-related deaths^3^. A substantial body of research has confirmed that smoking has become a major risk factor for the development of bladder cancer, with smoking increasing the associated risk by 2 to 4 times^4^. A number of studies have also indicated that smoking is closely associated with the development and prognosis of bladder cancer^5,6^. A meta-analysis based on secondary data by Ślusarczyk et al.^5^ confirms that smoking increases the risk of perioperative complications and mortality in bladder cancer surgery. Fortunately, smoking is also the most modifiable risk factor for bladder cancer. A series of national and global policies and measures have proven instrumental in the decline of smoking^7,8^. However, the imbalances in tobacco control between countries and increasing numbers of smokers caused by population growth are still huge problems we need to face.

GBD 2021 provides new data on smoking-attributable bladder cancer, and the newly released results should be analyzed and discussed promptly^9^. In this study, we used the data extracted from the GBD 2021 to examine the heterogeneous patterns of smoking-attributable bladder cancer across 204 countries and territories between 1990 and 2021, aiming to inform health policy formulation and guide the development of global and regional interventions for smoking, by providing a more comprehensive perspective.

## METHODS

### Research data

In this secondary dataset analysis, we sourced the data regarding the burden of bladder cancer attributable to smoking from the Global Health Data Exchange (GHDx) query tool (http://ghdx.healthdata.org/gbd-results-tool). The tool encompasses global data on 369 diseases and injuries and 87 associated risk factors across 204 countries and territories, spanning from 1990 to 2021^9^. The burden of these diseases was estimated by age, gender, country and region.

### Definitions

In the GBD 2021, bladder cancer was classified using the International Classification of Diseases (ICD) coding system. For bladder cancer incidence and mortality, the ICD-10 codes are C67, C67.1, C67.2, C67.3, C67.8, and C67.9 respectively, in the 10th Revision of the International Classification of Diseases (ICD-10)^10^. Exposure to smoking was characterized by the incidence of both current and past use of smoked tobacco products^11^. Therefore, we selected ‘smoking’ in the GBD query tool under the risk category, while ‘chewing tobacco’ and ‘secondhand smoke’ were not included in our selection. For those who currently smoke, it measures daily cigarette consumption and the total pack-years of tobacco use. In contrast, for ex-smokers, it estimates the distribution of the interval of time post-smoking cessation^12^. The sociodemographic index (SDI) of each country was also calculated by researchers in GBD 2021 as a composite indicator of economic and social conditions and therefore can be a reliable indicator for assessing health outcomes^13^. The calculation took into account the typical education level for people aged ≥15 years, the overall fertility rate among those aged <25 years, and the per capita lag distributed income (LDI). Utilizing the SDI, which has a scale from 0 to 1, nations or regions are categorized into five distinct groups: low, low-middle, middle, high-middle, and high. The data for these five distinct groups can be directly obtained through the GBD query tool^13^. The SDI data specific to GBD regions and countries can be downloaded from the GBD 2021 and matched with the corresponding regional and national data for further analysis.

### The burden prediction

To forecast the forthcoming trend in the burden of smoking-attributable bladder cancer, the Bayesian age-period-cohort (BAPC) model of integrated nested Laplace approximations (INLA), was further applied. The BAPC model demonstrated superior precision compared to alternative models^14^. And an INLA was employed to estimate the marginal posterior distributions within the BAPC model^15^. Furthermore, the baseline, negative, and positive reference were introduced for the better presentation of prediction results. The baseline reference was established according to the mortality in 2021. And 1% annual increase and a 1% annual decrease in 2021 mortality rates serve as the negative and positive references, respectively. Based on fluctuations in death rate, we ascertained the definite number of deaths. The *BAPC R* and *INLA-based R* packages were employed to perform the predictions.

### Statistical analysis

To assess the burden of bladder cancer attributable to smoking, death, DALYs, age-standardized death rate (ASDR) and age-standardized DALYs rate (ASDLR) were used in this research. The DALYs, a comprehensive metric for disease burden, are determined by combining the years of life with disability (YLDs) and the years of life lost (YLLs)^16^. Age-standardized rates were calculated by standardization to the global age structure as reported in the GBD study. The ASRs were calculated by standardizing to the global age structure from the GBD, from the formula:


$$\text{ASR}=\frac{\Sigma_{\text{i}=1}^{\text{A}}{\text{a}}_{\text{i}}{\text{w}}_{\text{i}}}{\Sigma_{\text{i}=1}^{\text{A}}{\text{w}}_{\text{i}}}\times 100000$$

where A represents the overall count of age groups, while and correspond to the age-specific ratios of the th age group and the number (or weight) of the same age group in the reference standard, respectively. The ASRs are presented per 100000 individuals, with the corresponding 95% uncertainty interval incorporated into the outcome. Using the Joinpoint software, the temporal trend in ASRs for smoking-related bladder cancer from 1990 to 2021 was measured using the APC (annual percent change) and the AAPC, as well as their 95% confidence intervals (95% CI)^17^. We configured the upper limit of joinpoints at five, which provided an adequate level of flexibility for trend detection without leading to overfitting of the model. If the APC and AAPC values, along with their corresponding 95% CIs, are above zero, the trend of ASRs is considered to be increasing. Conversely, if the APC and AAPC values, along with their 95% CIs, are below zero, the trend in ASRs is a decline. In order to identify the influential factors contributing to the burden of smoking-related bladder cancer, we conducted a Pearson correlation analysis to assess the association of AAPCs, ASRs and SDI. In this study, different subgroups (such as regions, SDI levels, and age groups) are considered independent in statistical analysis because they represent distinct demographic and socioeconomic characteristics with no significant overlap^18^. We used the R program (Version 4.3.2) to performed most statistics and visualization.

## RESULTS

### Temporal trends of bladder cancer attributable to smoking

**Figure 1:**
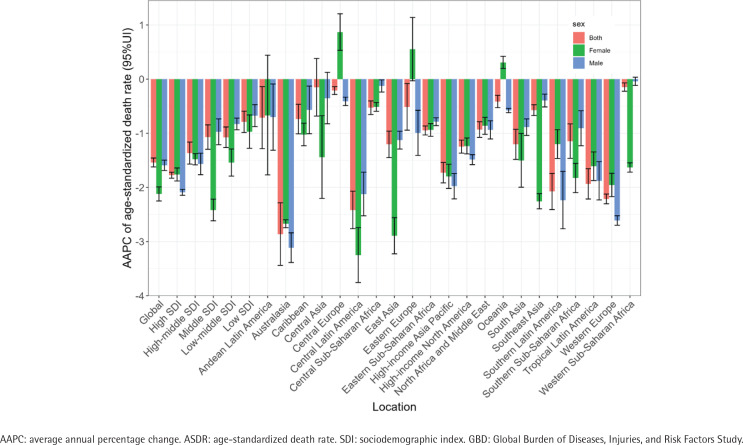
The AAPC of ASDR for bladder cancer attributable to smoking from 1990 to 2021, globally, in SDI regions, and 21 GBD regions, by sex

**Figure 2:**
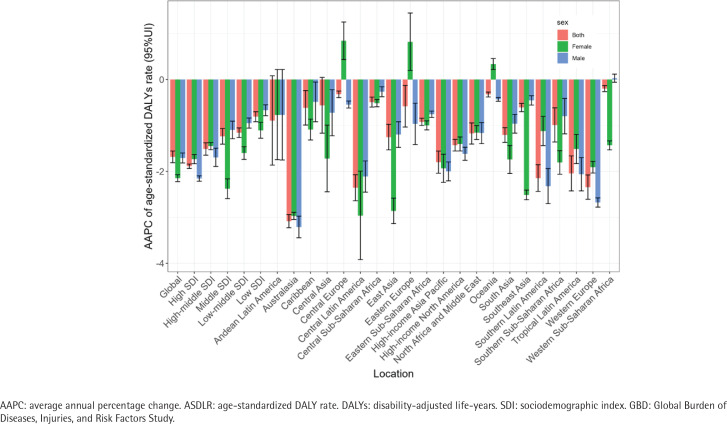
The AAPC of ASDLR for bladder cancer attributable to smoking from 1990 to 2021, globally, in SDI regions, and 21 GBD regions, by sex

In 2021, the number of deaths due to smoking-attributable bladder cancer was 58767 (accounting for 26.48% of the total bladder cancer-related deaths), compared to 41114 deaths in 1990. In 2021, the global number of DALYs due to smoking-attributable bladder cancer was 1.24 million, compared to 0.95 million DALYs in 1990 (Table 1). From 1990 to 2021, there was a significant increase in both the number of deaths and DALYs attributed to smoking-attributable bladder cancer. Between 1990 and 2021, the ASDR decreased annually by -1.54 (95% CI: -1.62 – -1.46) (Figure 1). Over the study period, the ASDLR also showed a decline, with an AAPC of -1.68 (95% CI: -1.81 – -1.56) (Figure 2). Especially from 2001 to 2007, the reduction in ASDR and ASDLR was the most significant, with APC of -2.30 (95% CI: -2.64 – -1.97) and -2.42 (95% CI: -2.74 – -1.65), respectively (Supplementary file Figure S2).

**Table 1:** The number, age-standardized rate, and temporal trend of bladder cancer burden attributable to smoking from 1990 to 2021

	*Deaths*					*DALYs*				
	*1990*		*2021*		*AAPC* *(95% CI)* *1990–2021*	*1990*		*2021*		*AAPC* *(95% CI)* *1990–2021*
	*Cases (×10^3^)*	*ASDR per 100000*	*Cases (×10^3^)*	*ASDR per 100000*		*Count (×10^3^)*	*ASDLR per 100000*	*Count (×10^3^)*	*ASDLR per 100000*	
**Overall**	41114.23	1.13	58766.87	0.70	-1.54 (-1.62 – -1.46)	945485.82	24.20	1238303.16	14.33	-1.68 (-1.81 – -1.56)
**Sex**										
Female	4691.39	0.24	5676.73	0.12	-2.12 (-2.25 – -1.99)	98445.19	4.71	111887.39	2.41	-2.15 (-2.22 – -2.07)
Male	36422.85	2.37	53090.14	1.45	-1.59 (-1.69 – -1.5)	847040.63	48.58	1126415.78	28.52	-1.71 (-1.82 – -1.6)
**Sociodemographic index**										
High	16539.95	1.46	19185.34	0.84	-1.77 (-1.83 – -1.72)	357231.23	32.04	375065.89	17.81	-1.89 (-1.94 – -1.84)
High–middle	14201.71	1.50	19649.72	0.99	-1.37 (-1.57 – -1.16)	337236.84	33.61	423433.61	21.06	-1.51 (-1.65 – -1.38)
Middle	6774.02	0.77	13622.64	0.55	-1.07 (-1.3 – -0.84)	163541.75	16.30	295301.59	11.12	-1.24 (-1.41 – -1.07)
Low–middle	2903.05	0.56	5102.59	0.40	-1.07 (-1.26 – -0.88)	70999.96	12.01	116525.07	8.33	-1.16 (-1.26 – -1.05)
Low	636.80	0.35	1127.42	0.27	-0.79 (-0.99 – -0.59)	15087.27	7.18	26247.54	5.53	-0.81 (-0.92 – -0.7)
**Region**										
Andean Latin America	37.40	0.21	87.95	0.16	-0.71 (-1.28 – -0.14)	781.69	4.03	1745.78	3.01	-0.9 (-1.86–0.08)
Australasia	240.51	1.00	235.40	0.41	-2.86 (-3.44 – -2.28)	5380.72	22.46	4531.46	8.45	-3.08 (-3.23 – -2.94)
Caribbean	181.66	0.73	318.01	0.59	-0.74 (-1.01 – -0.47)	3870.97	15.08	6681.57	12.35	-0.62 (-0.99 – -0.24)
Central Asia	312.56	0.68	481.04	0.65	-0.15 (-0.68 –0.38)	8359.53	17.25	11976.56	14.57	-0.56 (-1.17–0.05)
Central Europe	2415.29	1.60	3487.57	1.50	-0.21 (-0.28 – -0.14)	59560.78	38.61	77675.29	35.09	-0.32 (-0.39 – -0.25)
Central Latin America	266.26	0.37	424.87	0.18	-2.42 (-2.76 – -2.07)	5807.31	7.34	8905.53	3.60	-2.36 (-2.64 – -2.07)
Central Sub-Saharan Africa	48.14	0.26	99.79	0.22	-0.53 (-0.65 – -0.4)	1261.84	5.69	2666.11	4.86	-0.49 (-0.6 – -0.38)
East Asia	8667.06	1.22	17715.15	0.86	-1.2 (-1.45 – -0.96)	209256.52	25.10	371442.18	17.03	-1.25 (-1.53 – -0.97)
Eastern Europe	3079.06	1.08	3266.55	0.90	-0.51 (-0.94 – -0.08)	78417.37	27.06	78927.49	22.12	-0.58 (-1.03 – -0.13)
Eastern Sub-Saharan Africa	183.19	0.31	312.31	0.23	-0.95 (-1.03 – -0.86)	4328.21	6.28	7379.73	4.69	-0.92 (-1.00 – -0.84)
High-income Asia Pacific	1713.11	0.90	3038.30	0.53	-1.73 (-1.92 – -1.54)	36862.53	18.42	51133.99	10.56	-1.8 (-2.04 – -1.56)
High-income North America	4190.67	1.15	5554.16	0.80	-1.25 (-1.37 – -1.13)	95154.51	27.25	118016.81	17.77	-1.43 (-1.56 – -1.3)
North Africa and Middle East	1961.22	1.31	3819.64	0.97	-0.93 (-1.08 – -0.79)	51147.62	30.06	92413.45	20.98	-1.17 (-1.4 – -0.94)
Oceania	6.20	0.24	14.52	0.21	-0.41 (-0.53 – -0.3)	176.77	5.73	423.18	5.19	-0.33 (-0.38 – -0.27)
South Asia	2159.37	0.48	4190.35	0.33	-1.21 (-1.48 – -0.93)	49787.56	9.48	90949.81	6.43	-1.21 (-1.37 – -1.05)
South-East Asia	1005.57	0.48	2276.01	0.40	-0.57 (-0.67 – -0.47)	23545.94	9.77	51742.13	8.15	-0.61 (-0.7 – -0.52)
Southern Latin America	527.28	1.13	518.18	0.58	-2.08 (-2.41 – -1.74)	12781.46	27.07	11867.99	13.69	-2.15 (-2.44 – -1.85)
Southern Sub-Saharan Africa	161.55	0.67	246.01	0.47	-1.14 (-1.46 – -0.83)	3862.50	14.47	6229.01	10.60	-0.99 (-1.36 – -0.62)
Tropical Latin America	764.41	0.94	1258.34	0.50	-1.93 (-2.21 – -1.65)	17678.46	19.87	26514.15	10.32	-2.04 (-2.42 – -1.66)
Western Europe	13085.18	2.16	11199.94	1.08	-2.21 (-2.3 – -2.12)	274885.56	47.17	211726.19	22.82	-2.34 (-2.61 – -2.08)
Western Sub-Saharan Africa	108.56	0.15	222.77	0.14	-0.15 (-0.22 – -0.07)	2577.97	3.03	5354.75	2.84	-0.2 (-0.26 – -0.13)

ASDR: age-standardized death rate. ASDLR: age-standardized DALY rate. AAPC: average annual percentage change. DALYs: disability-adjusted life-years.

### Regional and national burdens of bladder cancer attributable to smoking

In 2021, the regions that experienced the most significant ASDR burden were Central Europe, Western Europe, and North Africa and Middle East (Table 1). Regarding the ASDLR, Central Europe, Western Europe, and Eastern Europe were the regions with the most substantial ASR burdens (Supplementary file Figures S1A and S1B). Andean Latin America, Oceania, and South-East Asia experienced the most significant rises in ASRs of smoking-attributable bladder cancer, whereas the largest decreases were observed in Western Europe, Australasia, and Southern Latin America (Supplementary file Table S1).

**Figure 3:**
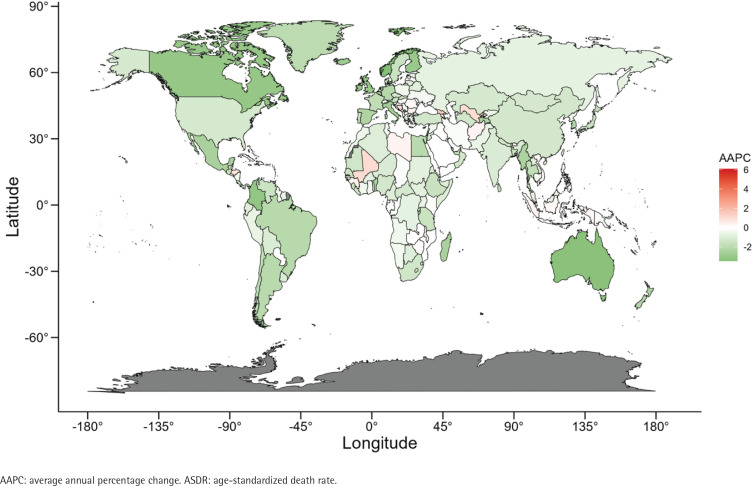
The AAPC of ASDR of bladder cancer attributable to smoking for both genders in 204 countries and territories from 1990 to 2021

**Figure 4:**
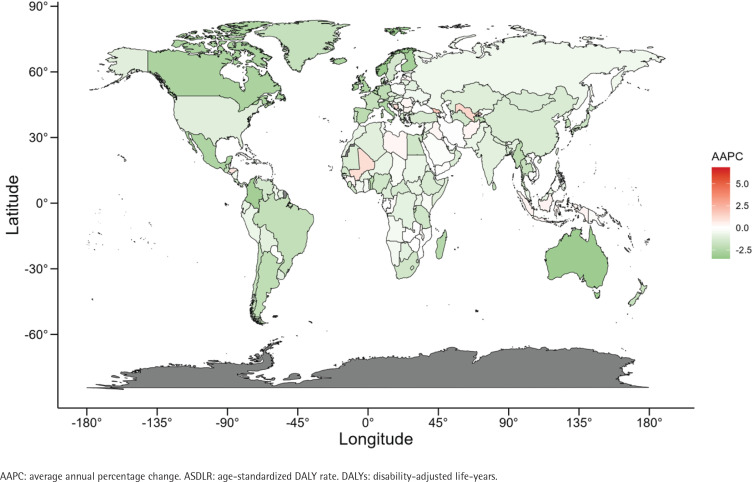
The AAPC of ASDLR of bladder cancer attributable to smoking for both genders in 204 countries and territories from 1990 to 2021

At the national level, when exploring the indicators of ASDR and ASDLR, the burden of bladder cancer was highest in Lebanon, Armenia, and Greece, positioning these countries as the top three worldwide (Supplementary file Table S2). It is worth noting that the highest increase in the ASDR burden was in Cabo Verde, Georgia, and Uzbekistan (Figure 3). Cabo Verde and Georgia also rank among the top three nations with the sharpest escalation in their ASDLR burden (Figure 4). San Marino, Norway, Australia, and Singapore have shown a significant downward trend in ASRs (Supplementary file Table S2).

### The relationship between SDI and smoking-attributable bladder cancer burden

When we consider the stratification of the SDI level, the regions with high-middle SDI and high SDI showed the highest ASDR and ASDLR for the study disease. Fortunately, across all SDI regions, there has been a substantial decrease in the smoking-attributed burden of bladder cancer (Figures 1 and 2).

As depicted in Supplementary file Figures 3A and 3B, a significant negative correlation was observed between the SDI and the AAPC of ASR of smoking-attributable bladder cancer for the year 2021. This correlation becomes particularly pronounced in countries and territories where the SDI ≥0.6 (AAPC of ASDR: ρ= -0.4172, p=5.852×10^-7^; AAPC of ASDLR: ρ= -0.4495, p=5.733×10^-8^). In contrast, no significant correlation was observed in countries and territories with an SDI <0.6. The study also detected a positive association between the ASRs of BCa attributable to smoking and the SDI in 2021, with this correlation being particularly evident in countries and regions with an SDI of ≥0.6 (ASDR: ρ= 0.2706, p=0.00163; ASDLR: ρ=0.2596, p=0.0025) (Supplementary file Figures S3C and S3D). Additionally, Supplementary file Figure 4 illustrates a comparable association between the ASRs of smoking-attributable bladder cancer and the SDI, but a downward trend in ASRs is observed when SDI exceeds approximately 0.75.

### Age composition and sex disparity of the burden of bladder cancer attributable to smoking

Considering that the GBD database only includes data on bladder cancer attributable to smoking for individuals aged ≥30 years, we stratify the population into three age groups: 30–49, 50–69 and ≥70 years. Across the globe, there was a rise in both bladder cancer deaths and DALYs attributed to smoking among all the three age groups over time. For most years between 1990 and 2021, the ≥70 years age group had the highest number of deaths, while the 50–69 years age group recorded the highest DALYs. The death rate and DALYs rate of smoking-associated bladder cancer were both highest in the ≥70 years age group and have declined during the study period (Supplementary file Figures S5A and S5B). The burden of smoking-attributable bladder cancer has remained relatively stable only in the 30–49 years age group.

The data analysis for the year 2021 further confirms that the age group of ≥70 years has the highest death and DALY rates (Supplementary file Figure S6). In most regions, the age group of ≥70 years has the highest number of deaths, while the age group of 50–69 years has the highest DALYs (Supplementary file Figures S7A and S7B). In terms of the percentage changes in mortality and DALYs rates, the burden in the age group of ≥70 years in Central Asia has increased significantly more than in other regions (Supplementary file Figures S8A and S8B).

There are significant disparities between males and females in the bladder cancer burden associated with smoking (Supplementary file Figure S6). Within every GBD region, the burden of bladder cancer attributable to smoking was significantly greater in males compared to females. Globally, 9.66% (5676.73) of bladder cancer deaths attributed to smoking occurred in females, in comparison to 90.34% (53090.14) in males in the year 2021 (Table 1). The same disparity was observed in the DALY metrics, with males exhibiting a tenfold higher number of DALYs compared to females. It is worth noting that an abnormal increasing trend in the burden of smoking-attributable bladder cancer in females is observed in regions such as Central Europe, Eastern Europe, and Oceania (Figure 2).

### The projection in the burden of bladder cancer attributable to smoking

The projections suggest that the global number of deaths from bladder cancer attributable to smoking will continue to show an increasing trend from 2022 to 2046 (Supplementary file Figure S9). The projected total mortality for both sexes is anticipated to rise from 59997.82 in 2021 to 90021.45 in 2046 (Supplementary file Table S7). Within this increase, males constitute the predominant share, exhibiting a 1.57-fold rise, compared to a 1.22-fold rise among females. Our forecasted outcomes for males and females were marginally below the optimistic reference of a 1% annual decrease. It is projected that the global ASDR will show a long-term downward trend. Particularly for the female population, the prediction of this downward trend is considered to have a higher degree of credibility (Supplementary file Figure S10).

## DISCUSSION

We have investigated the burden of BCa attributed to smoking globally, along with its temporal patterns spanning the years 1990 to 2021, and provided forecasted trends extending to 2046. Our research found that while the number of deaths and DALYs of smoking-attributable BCa rose in the majority of regions throughout the study period, there was a gradual decline in the age-standardized death rates and DALYs rates. The joinpoint analysis also indicated that especially from 2001 to 2007, the reduction in ASDR and ASDLR was the most significant. This is in line with the World Health Organization’s report on the trend of tobacco use from 2000 to 2025, which anticipates a continued decline in the prevalence of tobacco use^19^. This contradiction can be attributed to two reasons: on the one hand, the growth and aging of the global population have stimulated increased smoking and added to the disease burden^20^. On the other hand, it cannot be ignored that the prevalence of tobacco has shown a downward trend due to the implementation of health regulations worldwide, particularly in the early 21st century^21^. This is a positive change, but it also indicates the necessity for further exploration to identify shortcomings and then take action to reduce the global disease burden.

In further exploring the regional disparities in the disease burden of bladder cancer attributed to smoking, we found significant differences among various regions and countries. Analysis by SDI strata revealed that regions with high-middle SDI and high SDI values had the highest number of bladder cancer deaths and DALYs, as well as the highest age-standardized rates for both indicators. This corresponds with tobacco epidemiological survey results, which show that as of 2017, the smoking prevalence in high-, middle-, and low-income countries was 21.6%, 19.5%, and 11.2%, respectively^7^. Additionally, the GBD risk factor analysis for 2021 clearly identified smoking as the leading risk factor in high SDI regions^20^. It is evident that economic status is a major risk factor for increasing the burden of smoking-attributable bladder cancer. The analysis of the aforementioned results can be attributed to the following reasons. First, tobacco use is closely related to household economic status, leading to fewer smokers in low SDI regions and, consequently, a relatively lighter disease burden. Several studies^7,22^ have confirmed the above viewpoint, among which in 2024, Daniel et al.^7^ pointed out in a multicenter study that increased national income and higher relative prices of cigarettes are significantly associated with a lower likelihood of quitting. Moreover, more advanced medical resources in economically developed areas help to more accurately document and report a greater number of smoking-related deaths. However, it is also worth noting and contemplating that this may mean that in less economically developed regions, there may be more unrecorded data on the burden of bladder cancer attributable to smoking. These factors, influenced by different SDI levels, collectively affect the disease burden of bladder cancer, making it subject to the balance between the number of smokers and the level of medical services.

It is noteworthy that in regions with SDI ≥0.6, the AAPC exhibits a significant negative correlation with SDI, which intensifies with increasing SDI. This indicates that despite high-middle SDI and high SDI countries bearing a higher burden of smoking-attributable bladder cancer, the death and DALY rates for smoking-attributable bladder cancer are continuing to decline. Therefore, we speculate that in economically developed regions, where economic growth contributes to an increased burden of smoking-attributable bladder cancer, there is also a greater capacity for effective identification, treatment, and management of the disease. Additionally, in developed regions, heightened awareness of the poor prognosis of smoking-attributable bladder cancer increases the likelihood of quitting after diagnosis. Studies also show that raising awareness and encouraging smoking cessation have significant cost benefits^23,24^.

At the specific level of countries and regions, the three regions with the highest ASDR and ASDLR in 2021 were Central Europe, Western Europe, North Africa and the Middle East, and Central Europe, Western Europe, and Eastern Europe. All these regions, with the exception of North Africa and the Middle East, have a high SDI. The situation in North Africa and the Middle East is more complex. Some Gulf countries, due to their rich oil resources, have a higher per capita income and better healthcare systems, potentially classifying them as high SDI areas. Additionally, these regions have a long-standing historical and cultural tradition of waterpipe smoking, which is quite prevalent. In the analysis at the national level, we found that Lebanon in the Middle East has the highest burden, and studies show that the prevalence of waterpipe smoking in Lebanon is as high as 37.2%^25^. It is evident that the smoking culture is also one of the main reasons for the increased burden of smoking-attributable bladder cancer. Additionally, we found that, as indicated by the AAPC, the countries with the most significant downward trends in ASDR and ASDLR are economically developed nations such as San Marino, Norway, Australia, and Singapore. This suggests that although developed regions bear a heavier burden of bladder cancer attributed to smoking, these countries have made significant progress in reducing the burden of bladder cancer due to smoking. They are among the earliest adopters of tobacco control policies, such as smoke-free policies in public places, restrictions on tobacco advertising and promotion, increased taxation on tobacco products, and ratification of the Framework Convention on Tobacco Control (FCTC)^26^. This is consistent with current research findings, which suggest that strengthening tobacco control policies and enforcement at the national level is significantly associated with a greater likelihood of achieving smoking cessation^7,8^.

Significant differences in gender and age distribution are also key points that require our attention. Our study observed that males bear a significantly higher burden of bladder cancer attributed to smoking compared to females. This is consistent with relevant research findings, as reported in the 2023 Cancer Statistics, which indicate that the incidence rate of bladder cancer in males is 3–4 times higher than in females^4^. Sociocultural factors play a crucial role in the differences in smoking and drinking behaviors between men and women. Smoking, in some contexts, is a social behavior traditionally considered a symbol of masculinity^27^. Additionally, research suggests that gender-based burden differences may be explained by variations in the intensity of smoking between males and females^28^. However, our study requires more attention to the fact that, although the burden of smoking-attributed bladder cancer among females has been declining in most regions, an abnormal increasing trend is observed in Central Europe, Eastern Europe, and Oceania. These are consistent with related research indicating that female smoking behavior is most prevalent in European and Oceanian countries, with the total prevalence rates reaching as high as 38% and 36%, respectively^29^. This may be related to changes in the smoking culture among women in these regions and the greater difficulties women face in quitting smoking compared to men^30^. Moreover, an increasing number of studies have pointed out that the association between smoking and the risk of bladder cancer in women is stronger than in men^31^. Additionally, women have poorer prognoses after falling ill, and shorter survival times, which to some extent increases the death burden among women^32^. Therefore, we believe that for these regions, anti-smoking campaigns should be intensified among women, and when formulating and implementing anti-smoking policies, appropriate consideration should be given towards women.

Our research indicates that in the age group of ≥70 years, the death rate and DALY rate are the highest. This situation is closely related to the long-term exposure to cancer risks associated with smoking. A meta-analysis by Zhao et al.^33^ points out that the impact of smoking on bladder cancer has a dose-response relationship, increasing with the number of years and the amount of smoking. Additionally, it is related to the general decline in physiological and cognitive functions in the elderly, as well as increased susceptibility to comorbidities. Concurrently, the group aged 50–69 years experiences the highest number of DALYs due to smoking-attributable bladder cancer. This may be because there are fewer individuals aged ≥70 years compared to those aged 50–69 years, which is consistent with data from the World Bank^34^. It is worth noting that although the age group of 30–49 years does not show any statistical advantages, this should be related to the dose-response mechanism of smoking-induced bladder cancer incidence. Furthermore, the 1994 Surgeon General’s report in the United States stated that almost all adult smokers first used cigarettes during high school, and almost no one started smoking after the age of 20 years^35^. Additionally, multiple studies by Daniel et al.^7^ and Beard et al.^8^ have indicated that the difficulty of quitting smoking increases with age. Therefore, although the burden of bladder cancer is mainly concentrated in the middle-aged and elderly groups, tobacco control policies aimed at preventing youth smoking and promoting smoking cessation among young adults may be the most effective way to achieve a long-term reduction in smoking across all populations and is an effective method to alleviate the burden of smoking-related diseases^36^.

With the ongoing implementation of global tobacco control policies, the prevalence of smoking is predicted to continue declining, which also seems promising for the outlook of smoking-attributable bladder cancer. However, our predictive analysis reveals a concerning trend: from 2022 to 2046, despite the further decline in ASDR for both sexes, the global number of deaths due to smoking-induced bladder cancer is expected to continue increasing. This may be attributed to the growth of the global population, which could lead to a continuous increase in the number of smokers. Additionally, the dose-response relationship between smoking and bladder cancer means that the effectiveness of smoking cessation policies has a certain degree of latency^33^. These factors make the decline in ASDR insufficient to compensate for the absolute increase in disease burden caused by the aforementioned factors. This indicates that the situation regarding smoking-induced bladder cancer remains very serious. This trend highlights the urgency of taking further countermeasures.

### Limitations

This study has several limitations. Although the GBD study employs rigorous statistical methods to estimate data, the absence of actual data in some regions inevitably introduces bias into our research. For instance, the scarcity of cancer registries in certain areas with limited economic capacity has resulted in insufficient data on smoking-attributable bladder cancer. Moreover, the AAPC analysis relies on certain assumptions, such as the continuity of data and the linearity of trends. These assumptions may affect the interpretation of the results, especially when there are significant fluctuations or nonlinear trends in the data. Despite considering major confounding factors such as age, gender, region, and SDI, and assessing their impact on the burden of bladder cancer through stratified and correlation analyses, there may still be residual confounding factors that have not been identified or fully adjusted. These factors may affect the accuracy of the results. Therefore, this may have an impact on the determination of disease burden trends. Additionally, our study focuses on the burden of bladder cancer attributable to smoking, excluding other forms of tobacco consumption and other contributing factors.

## CONCLUSIONS

The present study reveals a global trend of increased deaths and DALYs due to smoking-attributable BCa, while observing a decline in ASRs. This not only highlights the severity of the burden of bladder cancer but also reflects the positive effects of public health interventions. We have found that the burden of smoking-attributable bladder cancer is particularly significant among males, the elderly, and individuals living in countries and regions with middle-high SDI and high SDI. Moreover, due to the unique mechanisms by which tobacco causes disease, the youth and female populations should be given priority. Furthermore, according to our projections, in the absence of further preventive measures, the number of deaths due to smoking-attributable bladder cancer is likely to rise over the next 25 years. Therefore, to mitigate the impact of smoking on bladder cancer, the implementation of sustained and effective tobacco control policies, along with public health interventions tailored to different population groups, is of paramount importance.

## Supplementary Material



## Data Availability

The data supporting this research are available from the following link: http://ghdx.healthdata.org/gbd-results-tool.
