# Supplementary material for: Contribution of smoking to the global burden of bladder cancer from 1990 to 2021 and projections to 2046
Source: Tob Induc Dis. 2025 Mar 28;23:10.18332/tid/202237. doi: 10.18332/tid/202237 (PMC11951971; doi:10.18332/tid/202237)
Supplement: Supplementary file 1 [file TID-23-44-s1.pdf]

Supplementary Material

1.Supplementary Data

Supplementary Figure 1. The rank of ASR burden for bladder cancer attributable to smoking over the past 32 years.

Supplementary Figure 2. APC of bladder cancer attributed to smoking in global and SDI regions (1990-2021).

Supplementary Figure 3. The relationship between SDI in 2021 and ASR and AAPC of bladder cancer attributable to smoking in 2021. A The correlation between AAPC of ASDR and SDI.

Supplementary Figure 4. The trend of bladder cancer burden attributable to smoking cross 21 GBD regions, 1990-2021.

Supplementary Figure 5. The burden of bladder cancer attributable to smoking for both genders in global by age group from 1990 to 2021.

Supplementary Figure 6. The death and DALY rate of bladder cancer attributable to smoking in global, SDI regions, and 21 GBD regions in 2021 by age group and sex.

Supplementary Figure 7. The proportion of deaths (A) or DALYs (B) of bladder cancer attributable to smoking in different age groups in 2021 across global, SDI regions, and 21 GBD regions.

Supplementary Figure 8. The percentage change in death (A) and DALY rate (B) of bladder cancer attributable to smoking in different age groups for global, SDI and 21 GBD regions.

Supplementary Figure 9. The observed (dashed line) and predicted (solid line) deaths of bladder cancer attributable to smoking from 1990 to 2046.

Supplementary Figure 10. The predicted trend of age-standardized death rate of bladder cancer attributable to smoking by sex until 2046.

Supplementary Table 1. Three regions with the largest and lowest number of deaths or DALYs of bladder cancer attributable to smoking.

Supplementary Table 2. Three countries with the largest and lowest number of deaths or DALYs of bladder cancer attributable to smoking.

Supplementary Table 3. The death cases and age-standardized death rate of bladder cancer attributable to smoking in 1990 and 2021, and its temporal trends from 1990 to 2021.

Supplementary Table 4. The DALYs and age-standardized DALY rate of bladder cancer attributable to smoking in 1990 and 2021, and its temporal trends from 1990 to 2021.

Supplementary Table 5. Age distribution of death rate (per 100,000) for bladder cancer attributable to smoking in different region by sex in 2021.

Supplementary Table 6. Age distribution of DALY rate (per 100,000) for bladder cancer attributable to smoking in different region by sex in 2021.

Supplementary Table 7. Prediction the burden of bladder cancer attributable to smoking

2. Supplementary Figures and Tables

(1) Supplementary Figures

Supplementary Figure 2. The rank of ASR burden for bladder cancer attributable to smoking over the past 32 years. A ASDR in bladder cancer; B age-standardized DALY rate in bladder cancer.

A

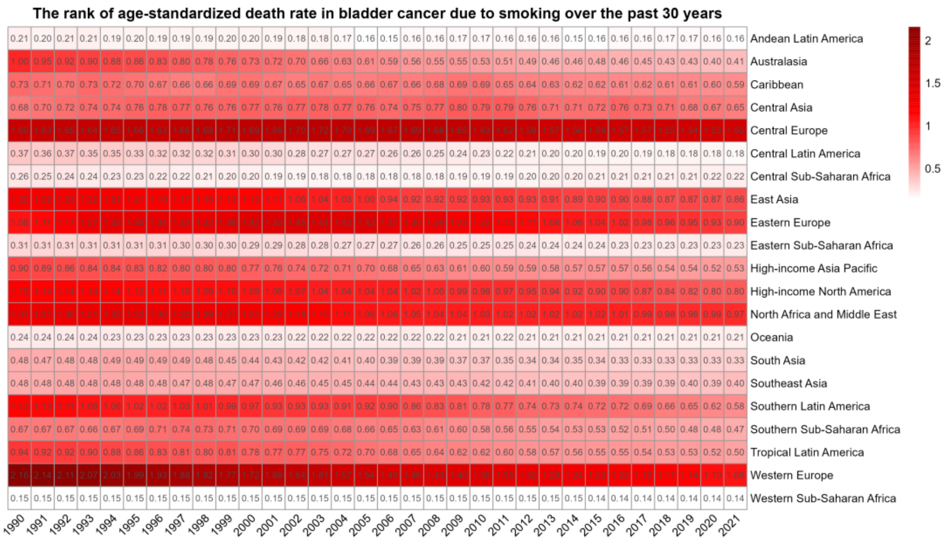

B

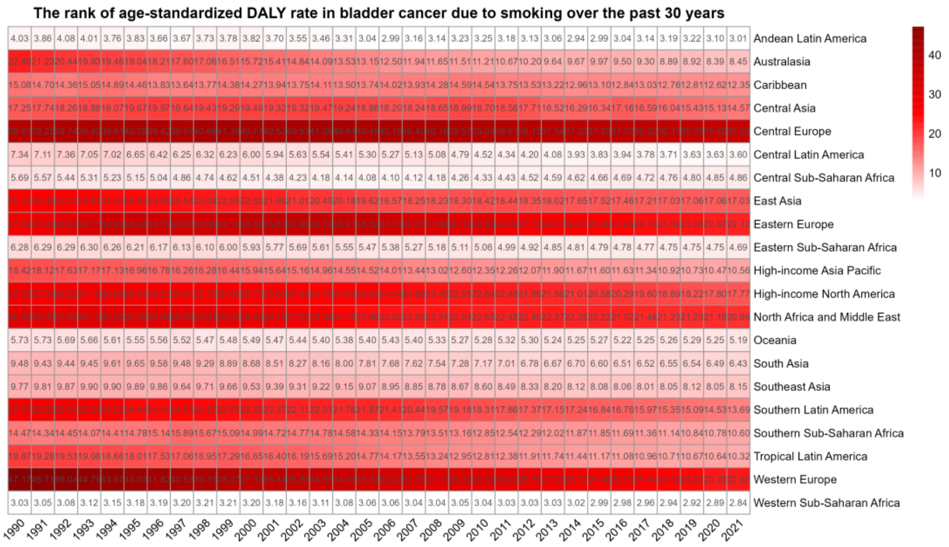

ASR: age-standardized rate. ASDR: age-standardized death rate

Supplementary Figure 2. APC of bladder cancer attributed to smoking in global and SDI regions (1990-2021) : A APC in death rate of bladder cancer attributable to smoking; B APC in DALYs rate of bladder cancer attributable to smoking.

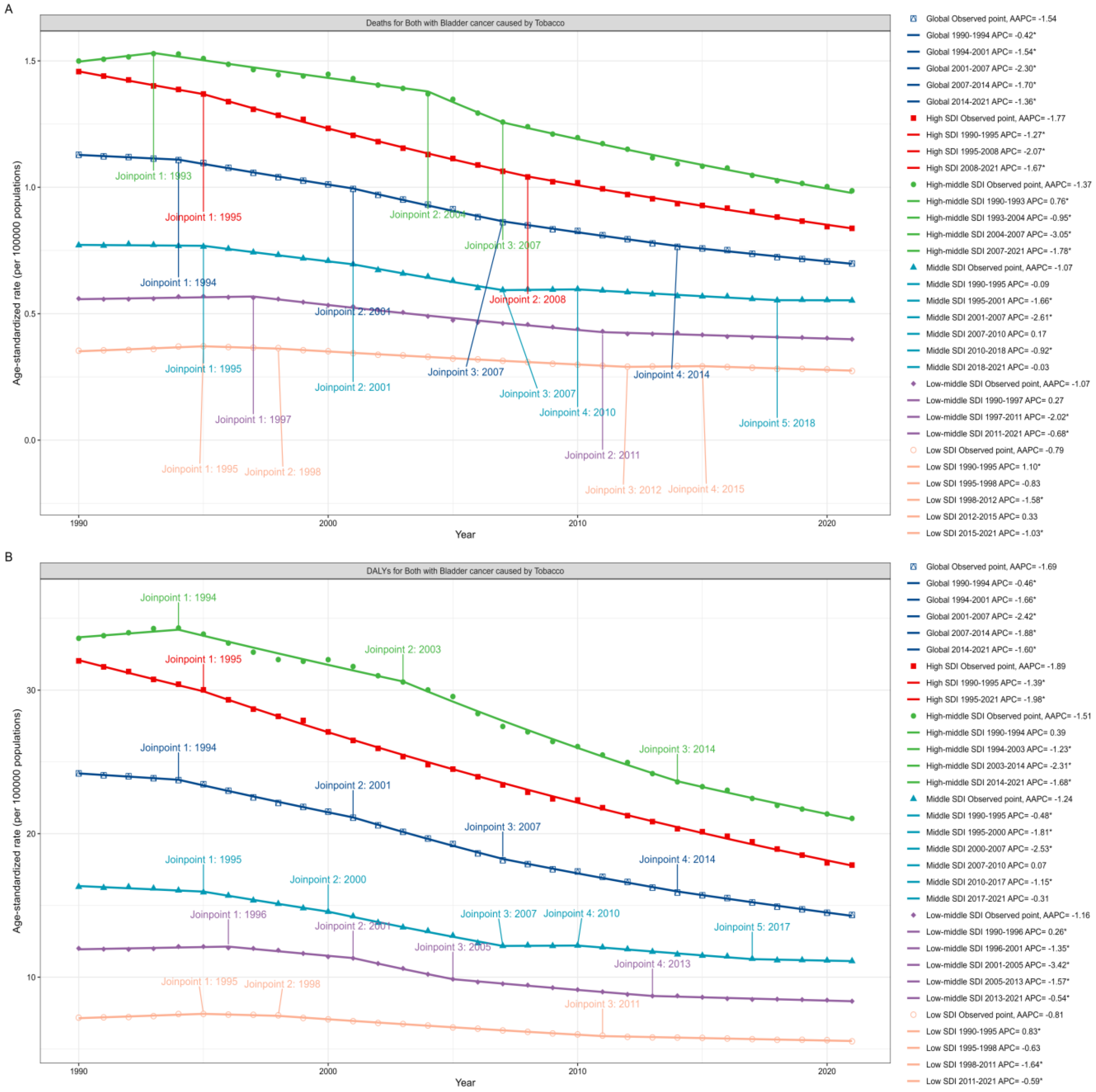

APC: Annual Percent Change. DALYs: Disability Adjusted Life Years. SDI: Socio-demographic Index.

Supplementary Figure 3. The relationship between SDI in 2021 and ASR and AAPC of bladder cancer attributable to smoking in 2021. A The correlation between AAPC of ASDR and SDI; B The correlation between AAPC of age-standardized DALY rate and SDI; C The correlation between ASDR and SDI; D The correlation between age-standardized DALY rate and SDI.

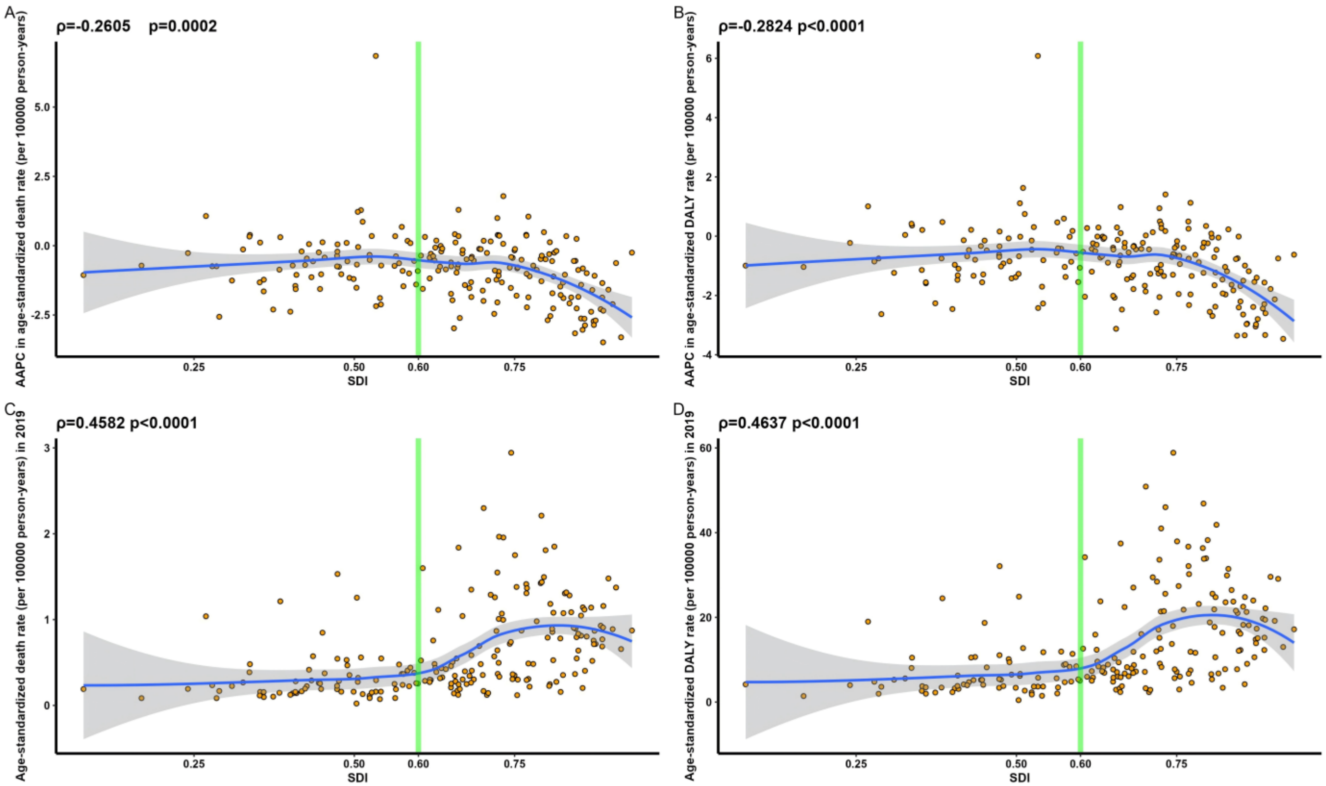

Each point represents a country or a region that were available on SDI data. AAPC average annual percentage change. ASR age-standardized rate. SDI socio-demographic index. ASDR age-standardized death rate.

Supplementary Figure 4. The trend of bladder cancer burden attributable to smoking cross 21 GBD regions, 1990-2021. A The trend of ASDR of bladder cancer; B The trend of age-standardized DALY rate in bladder cancer.

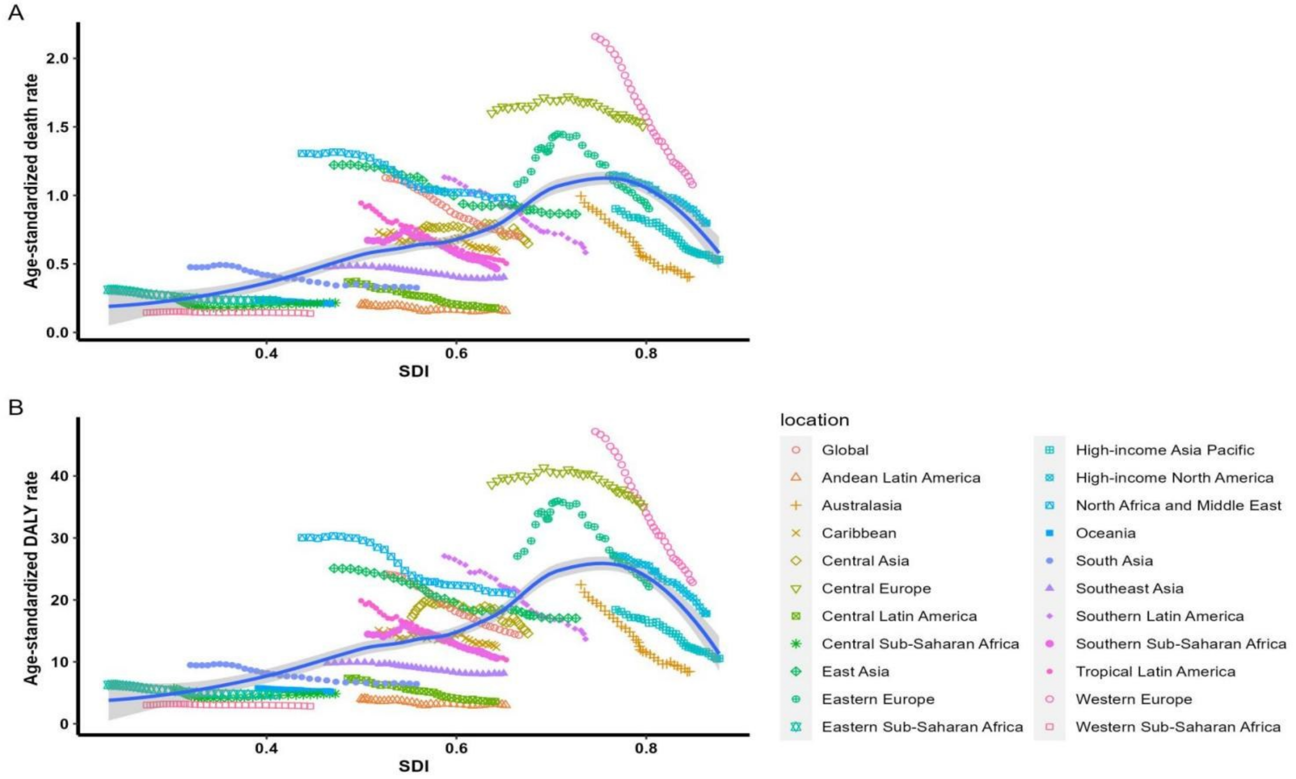

For each region, points from left to right depict estimates from each year from 1990 to 2021. GBD Global Burden of Diseases, Injuries, and Risk Factors Study. ASDR age-standardized death rate.

Supplementary Figure 5. The burden of bladder cancer attributable to smoking for both genders in global by age group from 1990 to 2021. A Global number of death cases and death rate; B Global number of DALYs and DALY rate.

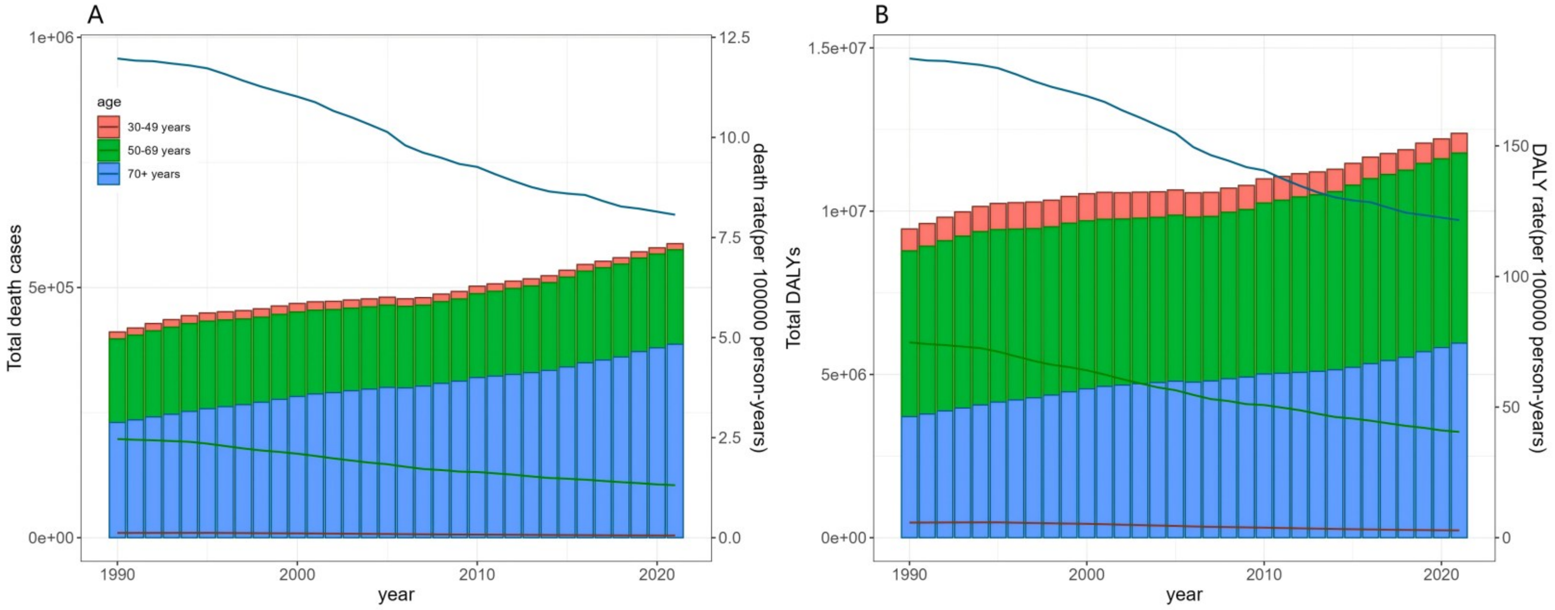

Each lines represent death/ DALY rates for age group, and each bars represent the number of deaths/DALYs. DALYs disability-adjusted life-years.

Supplementary Figure 6. The death and DALY rate of bladder cancer attributable to smoking in global, SDI regions, and 21 GBD regions in 2021 by age group and sex. A The death rate ; B The DALY rate.

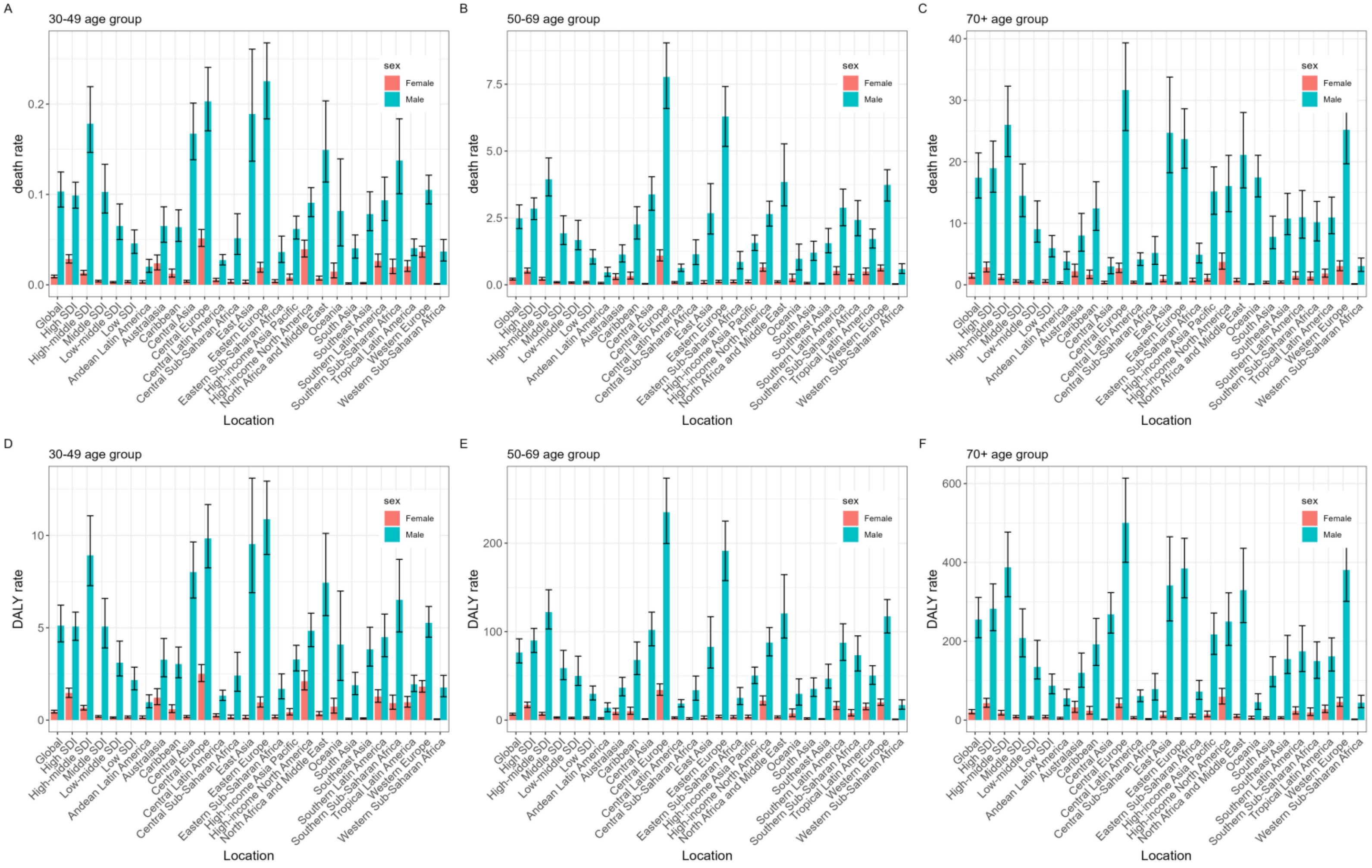

SDI socio-demographic index. GBD Global Burden of Diseases, Injuries, and Risk Factors Study. DALYs disability-adjusted life-years.

Supplementary Figure 7. The proportion of deaths (A) or DALYs (B) of bladder cancer attributable to smoking in different age groups in 2021 across global, SDI regions, and 21 GBD regions

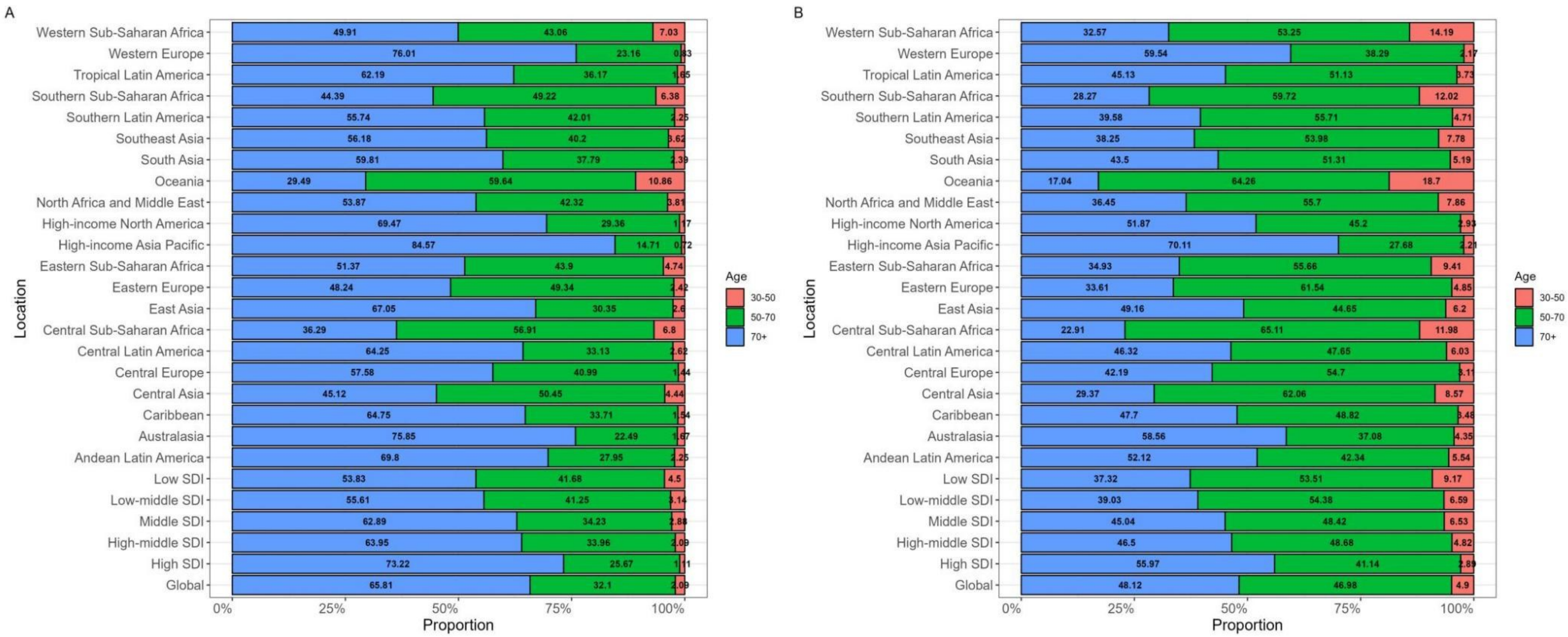

GBD Global Burden of Diseases, Injuries, and Risk Factors Study. SDI socio-demographic index.

Supplementary Figure 8. The percentage change in death (A) and DALY rate (B) of bladder cancer attributable to smoking in different age groups for global, SDI and 21 GBD regions.

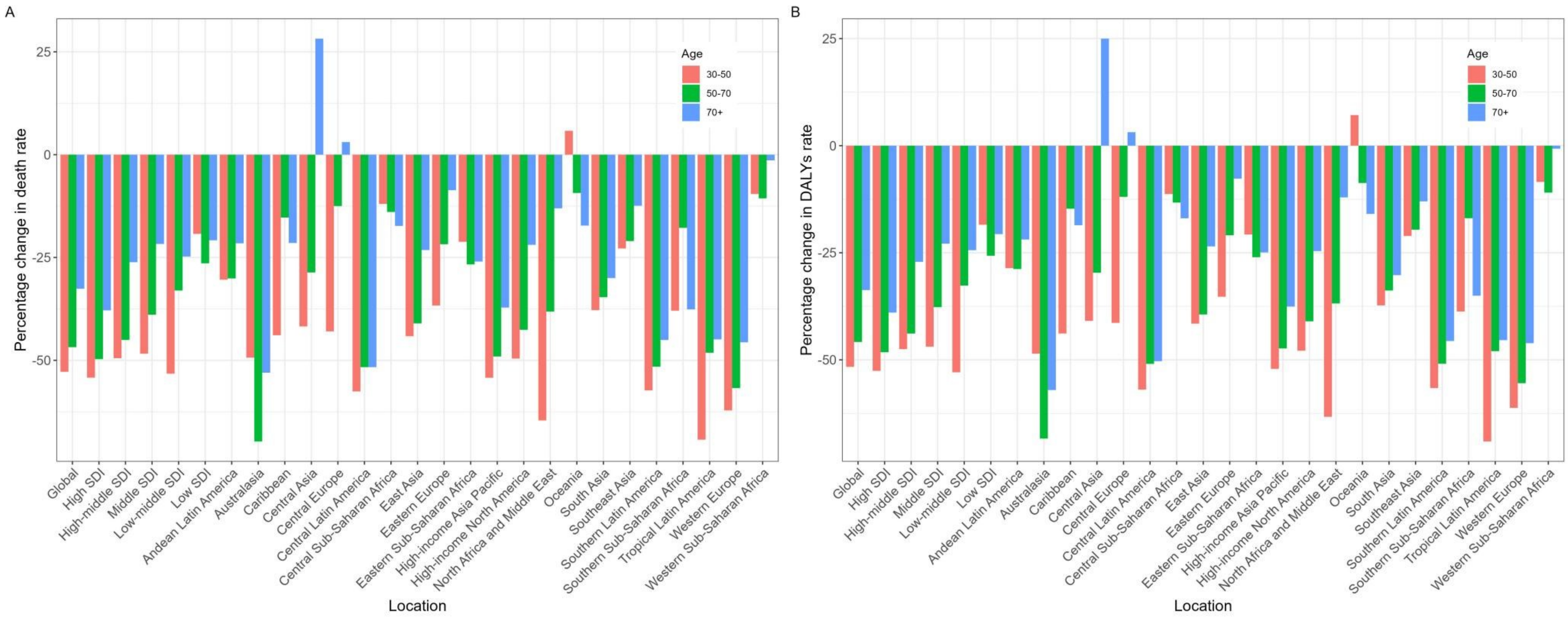

GBD Global Burden of Diseases, Injuries, and Risk Factors Study. SDI socio-demographic index

Supplementary Figure 9. The observed (dashed line) and predicted (solid line) deaths of bladder cancer attributable to smoking from 1990 to 2046.

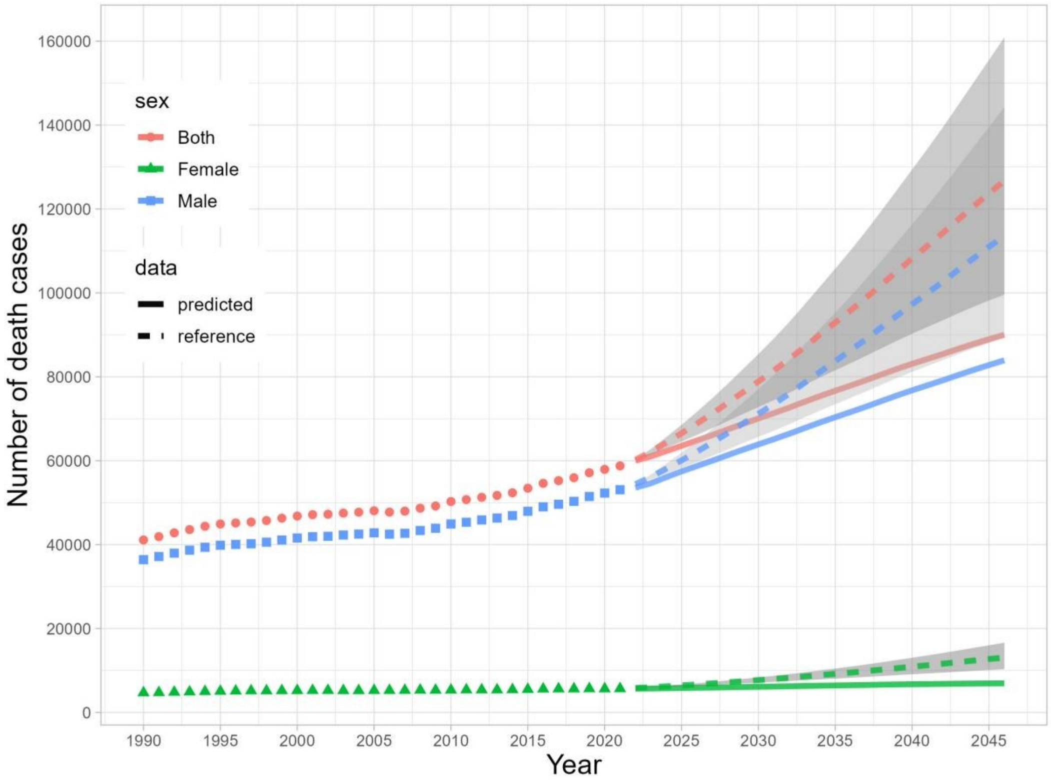

The upper bound of Shading represents the rate increased by 1% per year (pessimistic reference) and the lower bound represents decreased by 1% per year (optimistic reference) based on the rate observed in 2021.

Supplementary Figure 10. The predicted trend of age-standardized death rate of bladder cancer attributable to smoking by sex until 2046. A male's age-standardized death rate of bladder cancer attributable to smoking; B female's age-standardized death rate of bladder cancer attributable to smoking.

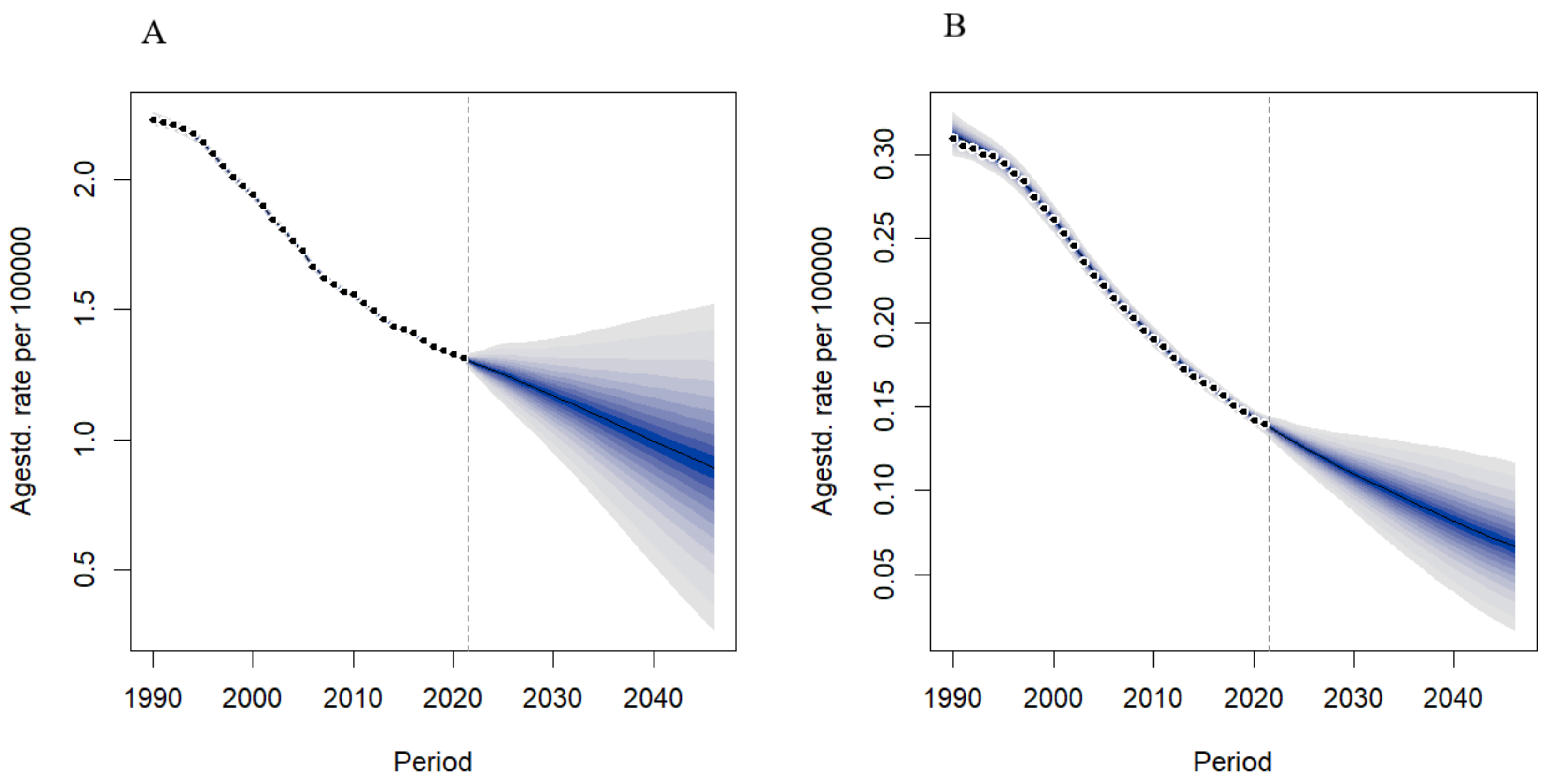

The confidence intervals of the forecasted values, that is, the shaded areas in the graph, provide a range of uncertainty for the predictions. Narrower confidence intervals suggest that the forecasted values are more precise, while wider confidence intervals indicate greater uncertainty in the forecasts.

(2) Supplementary Tables

Supplementary Table 1. Three regions with the largest and lowest number of deaths or DALYs of bladder cancer attributable to smoking.

measure	sex	top three regions			buttom three regions		
2021 ASR(per100,000 people)							
ASDR							
	both	Central Europe(1.50)	Western Europe(1.08)	North Africa and Middle East(0.97)	Western Sub-Saharan Africa(0.14)	Andean Latin America(0.16)	Central Latin America(0.18)
	female	Central Europe(0.34)	High-income North America(0.33)	Western Europe(0.29)	Western Sub-Saharan Africa(0.01)	Central Asia(0.01)	Central Sub-Saharan Africa(0.02)
	male	Central Europe(3.15)	Eastern Europe(2.46)	Western Europe(2.21)	Western Sub-Saharan Africa(0.28)	Andean Latin America(0.30)	Central Latin America(0.35)
Age Standardized DALY Rate							
	both	Central Europe(35.09)	Western Europe(22.82)	Eastern Europe(22.12)	Western Sub-Saharan Africa(2.84)	Andean Latin America(3.01)	Central Latin America(3.60)
	female	Central Europe(8.60)	High-income North America(7.53)	Western Europe(6.37)	Western Sub-Saharan Africa(0.24)	Central Asia(0.33)	Central Sub-Saharan Africa(0.42)
	male	Central Europe(69.42)	Eastern Europe(55.93)	Western Europe(44.59)	Andean Latin America(5.72)	Western Sub-Saharan Africa(5.76)	Central Latin America(6.89)

1990-2021 increase times

Death(cases)

both	Andean Latin America(2.35)	Oceania(2.34)	Southeast Asia(2.26)	Western Europe(0.86)	Australasia(0.98)	Southern Latin America(0.98)
female	Oceania(2.80)	Andean Latin America(2.62)	Central Sub-Saharan Africa(2.19)	Western Europe(0.86)	Central Asia(1.00)	Australasia(1.00)
male	Southeast Asia（2.33）	Andean Latin America(2.33)	Oceania(2.27)	Western Europe(0.84)	Southern Latin America(0.94)	Australasia(0.97)

DALY(Year)

both	Oceania（2.39）	Andean Latin America(2.23)	Southeast Asia(2.20)	Western Europe(0.77)	Australasia(0.84)	Southern Latin America(0.93)
female	Oceania(2.83)	Andean Latin America(2.42)	Central Sub-Saharan Africa(2.13)	Western Europe(0.81)	Australasia(0.86)	Central Asia(0.97)
male	Oceania(2.31)	Southeast Asia(2.27)	Andean Latin America(2.21)	Western Europe(0.76)	Australasia(0.84)	Southern Latin America(0.88)

AAPC

Death

both	Western Sub-Saharan Africa(-0.15)	Central Asia(-0.15)	Central Europe(-0.21)	Australasia(-2.86)	Central Latin America(-2.42)	Western Europe(-2.21)
female	Central Europe(0.87)	Eastern Europe(0.55)	Oceania(0.31)	Central Latin America(-3.25)	East Asia(-2.89)	Australasia(-2.67)
male	Western Sub-Saharan Africa(-0.04)	Central Sub-Saharan Africa(-0.13)	Central Asia(-0.35)	Australasia(-3.11)	Western Europe(-2.61)	Southern Latin America(-2.24)

DALY							
	both	Western Sub-Saharan Africa(-0.20)	Central Europe(-0.32)	Oceania(-0.33)	Australasia(-3.0)	Central Latin America(-2.36)	Western Europe(-2.34)
	female	Central Europe(0.84)	Eastern Europe(0.82)	Oceania(0.34)	Australasia(-2.97)	Central Latin America(-2.96)	East Asia(-2.86)
	male	Western Sub-Saharan Africa(0.03)	Central Sub-Saharan Africa(-0.27)	Oceania(-0.43)	Australasia(-3.21)	Western Europe(-2.68)	Southern Latin America(-2.32)

Supplementary Table 2. Three countries with the largest and lowest number of deaths or DALYs of bladder cancer attributable to smoking.

measure	sex	top three countries			buttom three countries		
2021ASR(per100,000 people)							
ASDR							
	both	Lebanon(2.94)	Armenia(2.30)	Greece(2.21)	Nigeria(0.02)	Guatemala(0.07)	Niger(0.08)
	female	Lebanon(0.7)	Denmark(0.66)	United Kingdom(0.59)	Morocco(<0.01)	Azerbaijan(<0.01)	Tajikistan(<0.01)
	male	Armenia(5.64)	Lebanon(5.59)	Georgia(5.01)	Nigeria(0.04)	Guatemala(0.14)	El Salvador(0.16)
Age Standardized DALY Rate							
	both	Lebanon(58.87)	Armenia(50.89)	Greece(46.88)	Nigeria(0.45)	Niger(1.41)	Guatemala(1.48)
	female	Lebanon(13.79)	Denmark(13.39)	Hungary(11.66)	Morocco(0.05)	Niger(0.08)	Palau(0.09)
	male	Armenia(120.3)	Lebanon(112.23)	Georgia(110.79)	Nigeria(0.87)	Guatemala(2.75)	Niger(2.83)
1990-2021 increase times							
Death(cases)							
	both	Cabo Verde(11.48)	Kuwait(5.4)	Djibouti(4.74)	Norway（0.53）	United Kingdom（0.65）	Belgium（0.72）
	female	Northern Mariana Islands(20.09)	American Samoa(7.08)	Cabo Verde(5.48)	Norway(0.51)	Burundi(0.63)	Portugal(0.65)

	male	Cabo Verde(11.74)	Kuwait(5.66)	Djibouti(4.84)	Norway(0.54)	United Kingdom(0.62)	Belgium(0.71)
DALY(Year)							
	both	Cabo Verde(12.7)	Kuwait(5.19)	United Arab Emirates(4.63)	Norway(0.5)	United Kingdom(0.57)	Belgium(0.67)
	female	Northern Mariana Islands(18.28)	American Samoa(7.03)	Cabo Verde(6.45)	Norway(0.47)	Myanmar(0.61)	Burundi(0.63)
	male	Cabo Verde(12.94)	Kuwait(5.39)	United Arab Emirates(4.69)	Norway(0.50)	United Kingdom(0.54)	Belgium(0.66)
AAPC							
Death							
	both	Cabo Verde(6.84)	Georgia(1.79)	Uzbekistan(1.29)	San Marino(-3.49)	Norway(-3.30)	Australia(-3.16)
	female	Northern Mariana Islands(5.24)	Cabo Verde(3.85)	American Samoa(3.74)	Mexico(-4.07)	Mauritius(-3.96)	Myanmar(-3.88)
	male	Cabo Verde(7.19)	Georgia(1.67)	Lesotho(1.18)	San Marino(-3.83)	Norway(-3.51)	Australia(-3.50)
DALY							
	both	Cabo Verde(6.08)	Lesotho(1.63)	Georgia(1.41)	Norway(-3.46)	Australia(-3.36)	Singapore(-3.34)
	female	Northern Mariana Islands(5.3)	Cabo Verde(4.08)	American Samoa(3.88)	Myanmar(-4.13)	Mauritius(-4.11)	Mexico(-3.83)
	male	Cabo Verde(6.10)	Lesotho(1.48)	Georgia(1.35)	Norway(-3.64)	Australia(-3.60)	San Marino(-3.57)

Supplementary Table 3. The death cases and age-standardized death rate of bladder cancer attributable to smoking in 1990 and 2021, and its temporal trends from 1990 to 2021.

Nation	Sex	Death Case No. (95% UI)		Change in absolute number (%)	Age-standardized death rate per 100,000 No. (95% UI)		1990-2021 AAPC No. (95 CI)
		1990	2021		1990	2021	
Afghanistan	Both	27.19(49.62 - 14.25)	38.36(57.7 - 21.75)	11.18	0.43(0.77 - 0.23)	0.48(0.71 - 0.28)	0.39(0.29 - 0.49)
Albania	Both	2.35(3.17 - 1.75)	5.42(7.86 - 3.66)	3.07	0.13(0.18 - 0.1)	0.12(0.18 - 0.08)	-0.26(-0.9 - 0.38)
Algeria	Both	60.15(79.76 - 40.41)	140.5(193.78 - 98.91)	80.35	0.74(0.98 - 0.48)	0.55(0.75 - 0.38)	-0.97(-1.1 - -0.85)
American Samoa	Both	0.07(0.09 - 0.05)	0.17(0.22 - 0.13)	0.10	0.33(0.46 - 0.24)	0.37(0.48 - 0.28)	0.39(0.09 - 0.69)
Andorra	Both	1.27(1.85 - 0.84)	1.82(2.64 - 1.14)	0.55	2.3(3.32 - 1.52)	1.14(1.65 - 0.71)	-2.38(-2.88 - -1.88)
Angola	Both	13.61(20.13 - 9.24)	35.78(50.24 - 25.49)	22.17	0.43(0.63 - 0.3)	0.37(0.53 - 0.26)	-0.43(-0.65 - -0.2)
Antigua and Barbuda	Both	0.21(0.27 - 0.16)	0.35(0.45 - 0.27)	0.14	0.37(0.47 - 0.29)	0.35(0.44 - 0.27)	-0.17(-1.32 - 0.99)
Argentina	Both	406.89(478.19 - 332.24)	365.32(447.01 - 287.3)	-41.57	1.24(1.47 - 1.01)	0.64(0.78 - 0.51)	-2.04(-2.63 - -1.45)
Armenia	Both	56.39(68.35 - 46.29)	101.3(124.75 - 81.65)	44.91	2.16(2.61 - 1.77)	2.3(2.83 - 1.85)	0.18(-0.67 - 1.03)
Australia	Both	194.63(232.55 - 162.17)	177.33(234.81 - 132.83)	-17.30	0.97(1.16 - 0.81)	0.36(0.48 - 0.28)	-3.16(-3.22 - -3.1)
Austria	Both	158.76(187.52 - 132.53)	158.56(199.47 - 126.41)	-0.20	1.28(1.5 - 1.08)	0.81(1 - 0.65)	-1.41(-2.07 - -0.75)

Azerbaijan	Both	35.24(49.14 – 26.03)	65.21(92.13 – 44.7)	29.97	0.72(0.99 – 0.53)	0.69(0.96 – 0.48)	-0.12(-0.48 – 0.25)
Bahamas	Both	0.35(0.46 – 0.26)	0.93(1.2 – 0.67)	0.58	0.24(0.32 – 0.18)	0.25(0.32 – 0.18)	0.12(-0.52 – 0.77)
Bahrain	Both	3.13(4.09 – 2.29)	8.02(11.89 – 5.32)	4.89	2.47(3.32 – 1.8)	1.38(1.96 – 0.93)	-1.8(-2.49 – -1.11)
Bangladesh	Both	209.94(336.91 – 126.16)	422.03(864.85 – 239.22)	212.10	0.52(0.84 – 0.31)	0.35(0.73 – 0.21)	-1.16(-1.71 – -0.6)
Barbados	Both	1.2(1.47 – 0.93)	1.63(2.21 – 1.12)	0.43	0.37(0.45 – 0.29)	0.31(0.42 – 0.21)	-0.44(-0.98 – 0.11)
Belarus	Both	171.63(196.17 – 148.03)	161.61(197.98 – 126.76)	-10.02	1.31(1.5 – 1.13)	0.98(1.2 – 0.77)	-0.87(-1.55 – -0.19)
Belgium	Both	373.62(436.5 – 314.57)	267.3(328.31 – 214.12)	-106.32	2.33(2.72 – 1.96)	1.05(1.27 – 0.86)	-2.62(-3.6 – -1.64)
Belize	Both	0.25(0.31 – 0.2)	0.76(0.95 – 0.58)	0.51	0.28(0.34 – 0.22)	0.29(0.36 – 0.21)	0.32(-0.47 – 1.11)
Benin	Both	3.92(5.76 – 2.76)	4.63(6.53 – 3.24)	0.71	0.21(0.31 – 0.15)	0.1(0.15 – 0.07)	-2.3(-2.49 – -2.12)
Bermuda	Both	0.72(0.93 – 0.54)	1.14(1.51 – 0.84)	0.43	1.18(1.53 – 0.89)	0.78(1.02 – 0.58)	-1.47(-2.02 – -0.93)
Bhutan	Both	0.38(0.67 – 0.19)	1.03(2.39 – 0.58)	0.65	0.21(0.38 – 0.11)	0.19(0.44 – 0.11)	-0.36(-0.49 – -0.23)
Bolivia (Plurinational State of)	Both	9.45(13.61 – 6.26)	20.64(30.41 – 13.38)	11.19	0.34(0.49 – 0.22)	0.25(0.37 – 0.16)	-0.91(-0.99 – -0.84)
Bosnia and Herzegovina	Both	43.41(53.6 – 35.39)	101.06(134.8 – 72.56)	57.65	1.15(1.4 – 0.93)	1.55(2.06 – 1.11)	1(0.74 – 1.25)
Botswana	Both	2.58(3.48 – 1.82)	5.16(7.24 – 3.68)	2.58	0.56(0.76 – 0.4)	0.42(0.58 – 0.31)	-0.85(-1.09 – -0.62)
Brazil	Both	755.02(860.91 – 654.35)	1235.22(1523.76 – 998.77)	480.19	0.96(1.09 – 0.82)	0.5(0.62 – 0.41)	-1.97(-2.25 – -1.69)

Brunei Darussalam	Both	0.91(1.23 – 0.69)	1.43(1.84 – 1.05)	0.52	1.06(1.42 – 0.8)	0.53(0.69 – 0.39)	-2.26(-2.61 – -1.91)
Bulgaria	Both	159.28(189.77 – 133.06)	206.55(261.07 – 164.05)	47.27	1.26(1.49 – 1.05)	1.41(1.79 – 1.13)	0.39(-0.13 – 0.92)
Burkina Faso	Both	4.18(6.08 – 2.81)	7.16(10.19 – 5.06)	2.98	0.11(0.15 – 0.07)	0.08(0.12 – 0.06)	-0.74(-0.93 – -0.56)
Burundi	Both	7.79(12.01 – 4.83)	6.7(9.95 – 4.37)	-1.08	0.38(0.58 – 0.23)	0.17(0.25 – 0.11)	-2.56(-2.64 – -2.49)
Cabo Verde	Both	0.06(0.18 – 0.03)	0.68(1.01 – 0.34)	0.62	0.03(0.08 – 0.01)	0.16(0.24 – 0.08)	6.84(5.13 – 8.59)
Cambodia	Both	21.81(32.28 – 15.82)	56.04(85.59 – 37.77)	34.23	0.59(0.86 – 0.43)	0.54(0.83 – 0.37)	-0.22(-0.32 – -0.11)
Cameroon	Both	8.69(11.53 – 6.37)	18.34(26.18 – 12.09)	9.64	0.22(0.29 – 0.16)	0.16(0.23 – 0.11)	-1.03(-1.15 – -0.9)
Canada	Both	603.35(695.78 – 510.69)	596.26(751.34 – 459.12)	-7.09	1.83(2.11 – 1.55)	0.76(0.94 – 0.59)	-2.73(-3.62 – -1.83)
Central African Republic	Both	3.28(4.93 – 2.06)	4.23(6.14 – 2.82)	0.95	0.33(0.49 – 0.21)	0.23(0.32 – 0.15)	-1.26(-1.38 – -1.14)
Chad	Both	5.42(7.61 – 3.36)	9.15(13.53 – 5.6)	3.73	0.21(0.3 – 0.13)	0.19(0.29 – 0.12)	-0.27(-0.36 – -0.18)
Chile	Both	54.83(67.13 – 43.3)	80.29(100.39 – 62.4)	25.46	0.56(0.69 – 0.44)	0.31(0.39 – 0.24)	-1.86(-2.65 – -1.07)
China	Both	8405.2(10516.63 – 5639.73)	17178.3(23470.82 – 12935.82)	8773.10	1.24(1.53 – 0.84)	0.87(1.18 – 0.66)	-1.23(-1.48 – -0.97)
Colombia	Both	56.13(68.01 – 46.76)	76.96(102.79 – 57.76)	20.83	0.35(0.43 – 0.29)	0.14(0.19 – 0.1)	-2.98(-3.65 – -2.31)
Comoros	Both	0.56(0.89 – 0.34)	1.12(1.96 – 0.59)	0.56	0.36(0.56 – 0.22)	0.28(0.49 – 0.15)	-0.76(-0.85 – -0.66)
Congo	Both	3.56(5.17 – 2.19)	8.36(12.13 – 5.69)	4.80	0.39(0.57 – 0.25)	0.41(0.58 – 0.28)	0.17(0.05 – 0.3)

Cook Islands	Both	0.07(0.09 – 0.05)	0.13(0.17 – 0.09)	0.06	0.61(0.8 – 0.44)	0.48(0.65 – 0.35)	-0.72(-0.8 – -0.63)
Costa Rica	Both	9.98(11.9 – 8.26)	19.26(24.52 – 14.87)	9.29	0.61(0.73 – 0.5)	0.35(0.45 – 0.27)	-1.62(-2 – -1.24)
Croatia	Both	117.53(140.75 – 97.87)	176.27(218.26 – 138.58)	58.73	2.08(2.5 – 1.72)	1.81(2.23 – 1.43)	-0.49(-0.6 – -0.32)
Cuba	Both	120.27(140.73 – 99.59)	210.85(259.48 – 164.34)	90.58	1.19(1.39 – 0.98)	1.04(1.28 – 0.81)	-0.28(-1.05 – 0.07)
Cyprus	Both	15.54(21.08 – 11.58)	27.21(37.65 – 19.53)	11.67	2.41(3.28 – 1.74)	1.28(1.78 – 0.92)	-2.08(-0.41 – -0.14)
Czechia	Both	259.29(308.5 – 215.92)	301.01(380.78 – 234.16)	41.72	1.82(2.16 – 1.51)	1.31(1.65 – 1.01)	-1.05(-2.41 – -1.75)
C么te d'Ivoire	Both	7.82(10.7 – 5.25)	19.95(28.92 – 13.18)	12.13	0.26(0.36 – 0.18)	0.22(0.32 – 0.15)	-0.46(-1.31 – -0.79)
Democratic People's Republic of Korea	Both	83.88(115.17 – 57.31)	154.86(229.89 – 111.35)	70.98	0.58(0.79 – 0.4)	0.48(0.71 – 0.34)	-0.63(-0.67 – -0.59)
Democratic Republic of the Congo	Both	25.51(36.08 – 16.67)	47.33(69.33 – 31.02)	21.82	0.19(0.28 – 0.13)	0.15(0.22 – 0.1)	-0.86(-0.97 – -0.76)
Denmark	Both	209.06(265.93 – 168.47)	194.59(233.29 – 160.5)	-14.46	2.44(3.08 – 1.98)	1.48(1.77 – 1.23)	-1.59(-2.34 – -0.83)
Djibouti	Both	0.49(0.76 – 0.3)	2.32(3.83 – 1.35)	1.83	0.51(0.79 – 0.33)	0.53(0.87 – 0.32)	0.1(0.01 – 0.19)
Dominica	Both	0.25(0.35 – 0.18)	0.3(0.43 – 0.21)	0.04	0.42(0.57 – 0.3)	0.36(0.52 – 0.25)	-0.44(-0.51 – -0.36)
Dominican Republic	Both	10.28(13.25 – 7.69)	28.5(40.69 – 19.55)	18.22	0.34(0.44 – 0.25)	0.3(0.42 – 0.2)	-0.29(-0.83 – 0.25)
Ecuador	Both	11.2(13.67 – 8.92)	26.98(35.93 – 19.12)	15.79	0.24(0.3 – 0.19)	0.17(0.23 – 0.12)	-0.79(-2.52 – 0.97)
Egypt	Both	634.2(803.62 – 394.34)	802.82(1427.65 – 531.06)	168.62	2.62(3.38 – 1.9)	1.6(2.94 – 1.03)	-1.56(-2.03 – -1.08)

El Salvador	Both	2.21(2.99 – 1.58)	5.31(7.38 – 3.68)	3.10	0.08(0.11 – 0.06)	0.09(0.12 – 0.06)	0.34(-0.46 – 1.15)
Equatorial Guinea	Both	0.46(0.7 – 0.29)	1.16(1.87 – 0.7)	0.69	0.27(0.4 – 0.18)	0.28(0.46 – 0.17)	0.12(-0.07 – 0.3)
Eritrea	Both	1.76(2.7 – 1.04)	3.32(5.17 – 2.1)	1.56	0.18(0.27 – 0.1)	0.14(0.22 – 0.09)	-0.71(-0.81 – -0.62)
Estonia	Both	21.81(25.98 – 18.27)	25.1(30.73 – 19.86)	3.30	1.05(1.25 – 0.88)	0.89(1.09 – 0.72)	-0.55(-1.07 – -0.02)
Eswatini	Both	0.7(0.97 – 0.49)	1.03(1.52 – 0.66)	0.33	0.31(0.43 – 0.22)	0.22(0.32 – 0.14)	-1(-1.13 – -0.87)
Ethiopia	Both	25.51(41.13 – 13.97)	35.56(59.93 – 21.16)	10.06	0.14(0.23 – 0.08)	0.1(0.16 – 0.06)	-1.23(-1.36 – -1.09)
Fiji	Both	0.95(1.24 – 0.65)	1.83(2.65 – 1.03)	0.88	0.29(0.38 – 0.2)	0.25(0.35 – 0.14)	-0.49(-0.92 – -0.06)
Finland	Both	67.27(80.08 – 54.9)	55.46(70.22 – 42.73)	-11.81	0.92(1.09 – 0.75)	0.4(0.5 – 0.31)	-2.87(-2.98 – -2.76)
France	Both	1698.86(1981.77 – 1420.83)	1669.28(2071.15 – 1311.01)	-29.58	1.97(2.28 – 1.65)	1.09(1.32 – 0.87)	-1.87(-2.07 – -1.67)
Gabon	Both	1.72(2.42 – 1.11)	2.94(4.38 – 2.06)	1.22	0.32(0.45 – 0.21)	0.32(0.48 – 0.22)	-0.01(-0.12 – 0.11)
Gambia	Both	0.6(0.82 – 0.38)	1.23(1.76 – 0.83)	0.63	0.2(0.27 – 0.12)	0.14(0.21 – 0.1)	-1.05(-1.68 – -0.42)
Georgia	Both	75.98(96.28 – 58.39)	118.9(143.02 – 96.5)	42.92	1.2(1.52 – 0.92)	1.96(2.37 – 1.58)	1.79(0.04 – 3.56)
Germany	Both	2338.63(2686.02 – 1958.36)	1902.96(2332.83 – 1492.44)	-435.67	1.75(1.99 – 1.47)	0.89(1.08 – 0.71)	-2.11(-2.56 – -1.65)
Ghana	Both	9.39(12.76 – 6.44)	22.88(34.64 – 15.27)	13.49	0.21(0.3 – 0.15)	0.19(0.28 – 0.12)	-0.38(-0.55 – -0.22)
Greece	Both	507.13(571.55 – 439.31)	598.42(711.72 – 495.31)	91.30	3.27(3.68 – 2.83)	2.21(2.61 – 1.86)	-1.28(-1.73 – -0.83)

Greenland	Both	0.57(0.72 – 0.37)	0.7(0.98 – 0.47)	0.13	1.98(2.49 – 1.3)	1.07(1.47 – 0.72)	-1.89(-2.21 – -1.57)
Grenada	Both	0.25(0.32 – 0.18)	0.34(0.44 – 0.25)	0.09	0.33(0.43 – 0.25)	0.3(0.39 – 0.23)	-0.16(-0.72 – 0.41)
Guam	Both	0.18(0.24 – 0.14)	0.48(0.62 – 0.36)	0.30	0.25(0.33 – 0.18)	0.23(0.29 – 0.17)	-0.3(-0.83 – 0.22)
Guatemala	Both	3.48(4.39 – 2.71)	7.46(9.72 – 5.58)	3.98	0.13(0.17 – 0.1)	0.07(0.09 – 0.05)	-1.89(-2.88 – -0.88)
Guinea	Both	10.3(14.48 – 7)	18.94(27.72 – 12.4)	8.64	0.35(0.49 – 0.23)	0.39(0.57 – 0.25)	0.33(0.25 – 0.41)
Guinea-Bissau	Both	0.55(0.84 – 0.31)	1.02(1.53 – 0.66)	0.47	0.15(0.23 – 0.09)	0.16(0.24 – 0.1)	0.11(-0.06 – 0.29)
Guyana	Both	0.89(1.12 – 0.7)	1.26(1.73 – 0.87)	0.37	0.26(0.32 – 0.2)	0.21(0.29 – 0.15)	-0.6(-1.37 – 0.16)
Haiti	Both	7.72(11.82 – 4.94)	11.46(16.92 – 6.9)	3.74	0.27(0.41 – 0.17)	0.19(0.29 – 0.11)	-1.03(-1.13 – -0.94)
Honduras	Both	1.88(2.6 – 1.3)	7.45(11.3 – 4.59)	5.57	0.1(0.14 – 0.07)	0.13(0.2 – 0.08)	0.86(0.7 – 1.02)
Hungary	Both	259.83(305.54 – 215.71)	281.87(354.33 – 225.23)	22.04	1.72(2.03 – 1.44)	1.42(1.78 – 1.14)	-0.48(-0.99 – 0.03)
Iceland	Both	4.91(5.76 – 4.11)	4.44(5.68 – 3.43)	-0.47	1.66(1.94 – 1.39)	0.72(0.91 – 0.56)	-2.7(-3.03 – -2.36)
India	Both	1162(1512.74 – 792.41)	2494.4(3204.24 – 1952.1)	1332.41	0.31(0.41 – 0.21)	0.24(0.3 – 0.19)	-0.91(-1.44 – -0.38)
Indonesia	Both	243.06(329.58 – 184.22)	667.52(1192.55 – 419.12)	424.46	0.3(0.42 – 0.23)	0.35(0.63 – 0.21)	0.41(0.35 – 0.48)
Iran (Islamic Republic of)	Both	115.07(151.69 – 82.42)	340.33(423 – 252.44)	225.26	0.5(0.67 – 0.36)	0.48(0.6 – 0.35)	-0.12(-0.36 – 0.11)
Iraq	Both	122.37(171.03 – 83.85)	347.31(502.41 – 227.25)	224.94	1.69(2.36 – 1.16)	1.84(2.63 – 1.22)	0.34(0.04 – 0.64)
Ireland	Both	74.77(86.23 – 63.72)	60.33(75.35 – 47.12)	-14.44	1.78(2.06 – 1.52)	0.71(0.89 – 0.56)	-2.86(-3.32 – -2.4)

Israel	Both	72.59(86.03 – 59.81)	111.76(140.87 – 87.31)	39.17	1.49(1.79 – 1.23)	0.86(1.07 – 0.67)	-1.72(-2.13 – -1.3)
Italy	Both	2382.27(2718.66 – 2025.75)	1942.95(2357.32 – 1541.12)	-439.31	2.58(2.94 – 2.19)	1.18(1.41 – 0.96)	-2.54(-2.81 – -2.27)
Jamaica	Both	8.68(10.72 – 6.98)	12.49(17.27 – 8.55)	3.81	0.47(0.58 – 0.38)	0.4(0.56 – 0.28)	-0.4(-2.22 – 1.46)
Japan	Both	1410.35(1588.02 – 1236.57)	2375.52(2936.84 – 1845.69)	965.17	0.86(0.97 – 0.75)	0.52(0.62 – 0.42)	-1.6(-1.86 – -1.34)
Jordan	Both	14.54(19.11 – 10.58)	60.29(85.13 – 41.93)	45.76	1.36(1.79 – 0.99)	1.01(1.41 – 0.71)	-0.93(-1.14 – -0.73)
Kazakhstan	Both	90.14(124.37 – 65.81)	87.74(109.21 – 68.52)	-2.40	0.72(0.98 – 0.52)	0.5(0.62 – 0.39)	-1.08(-1.63 – -0.53)
Kenya	Both	8.22(12.2 – 5.22)	20.52(26.67 – 14.78)	12.30	0.12(0.18 – 0.08)	0.11(0.15 – 0.08)	-0.33(-0.5 – -0.16)
Kiribati	Both	0.05(0.06 – 0.04)	0.1(0.13 – 0.07)	0.05	0.14(0.17 – 0.1)	0.15(0.19 – 0.11)	0.35(0.27 – 0.42)
Kuwait	Both	3.33(4.03 – 2.65)	17.97(23.26 – 13.6)	14.64	0.72(0.88 – 0.57)	0.8(1.03 – 0.6)	0.25(-0.64 – 1.14)
Kyrgyzstan	Both	17.31(21.14 – 13.46)	23.54(30.21 – 18.35)	6.22	0.6(0.74 – 0.47)	0.52(0.67 – 0.4)	-0.36(-1.3 – 0.59)
Lao People's Democratic Republic	Both	10.07(15.91 – 5.97)	17.5(26.92 – 11.21)	7.43	0.56(0.87 – 0.33)	0.47(0.73 – 0.3)	-0.56(-0.64 – -0.48)
Latvia	Both	43.32(50.57 – 37.04)	53.81(66.07 – 42.74)	10.49	1.2(1.39 – 1.02)	1.32(1.62 – 1.05)	0.34(-0.11 – 0.8)
Lebanon	Both	64.86(90.99 – 42.03)	188.72(258.8 – 136.46)	123.86	3.38(4.76 – 2.2)	2.94(4.01 – 2.12)	-0.41(-0.6 – -0.22)
Lesotho	Both	2.92(4.27 – 2.01)	5.43(8.24 – 3.1)	2.51	0.39(0.56 – 0.27)	0.56(0.83 – 0.34)	1.28(1.02 – 1.54)
Liberia	Both	1.79(2.58 – 1.19)	2.14(3.11 – 1.38)	0.35	0.17(0.25 – 0.12)	0.12(0.17 – 0.07)	-1.32(-1.47 – -1.18)

Libya	Both	29.15(42.25 – 18.81)	85.56(123.66 – 59.14)	56.41	1.76(2.56 – 1.11)	1.97(2.89 – 1.36)	0.38(0.05 – 0.71)
Lithuania	Both	61.68(71.98 – 51.03)	71.14(85.99 – 56.77)	9.46	1.36(1.59 – 1.13)	1.14(1.38 – 0.93)	-0.82(-3.03 – 1.44)
Luxembourg	Both	10.38(12.31 – 8.53)	10.27(13.07 – 7.97)	-0.11	1.85(2.19 – 1.51)	0.93(1.17 – 0.73)	-2.29(-2.84 – -1.74)
Madagascar	Both	10.36(14.12 – 7.37)	9.31(13.25 – 6.04)	-1.05	0.26(0.36 – 0.19)	0.12(0.18 – 0.08)	-2.38(-2.53 – -2.23)
Malawi	Both	31.82(40.76 – 23.64)	71.74(98.19 – 49.16)	39.92	1.11(1.45 – 0.83)	1.21(1.66 – 0.85)	0.31(0.16 – 0.45)
Malaysia	Both	67.19(97.04 – 37.72)	174.74(238.9 – 115.72)	107.56	0.84(1.22 – 0.46)	0.72(0.98 – 0.47)	-0.79(-1.22 – -0.35)
Maldives	Both	0.45(0.66 – 0.29)	1.2(1.56 – 0.89)	0.75	0.78(1.14 – 0.5)	0.46(0.6 – 0.34)	-1.74(-1.94 – -1.53)
Mali	Both	21.93(29.02 – 15.68)	66.62(94.62 – 45.99)	44.69	0.75(1.01 – 0.54)	1.04(1.49 – 0.71)	1.07(0.89 – 1.25)
Malta	Both	8(9.58 – 6.61)	8.01(10.17 – 6.26)	0.01	1.87(2.23 – 1.53)	0.79(0.99 – 0.62)	-2.69(-3.26 – -2.11)
Marshall Islands	Both	0.05(0.07 – 0.03)	0.11(0.16 – 0.07)	0.07	0.3(0.45 – 0.2)	0.37(0.53 – 0.23)	0.68(0.49 – 0.86)
Mauritania	Both	2.09(3.03 – 1.46)	3.04(4.95 – 1.75)	0.95	0.23(0.33 – 0.16)	0.16(0.26 – 0.09)	-1.2(-1.32 – -1.08)
Mauritius	Both	7.81(9.01 – 6.73)	10.14(11.83 – 8.6)	2.32	1.22(1.42 – 1.04)	0.57(0.67 – 0.48)	-2.46(-3.25 – -1.66)
Mexico	Both	153.94(180.85 – 128.75)	224.52(277.71 – 175.18)	70.58	0.44(0.52 – 0.36)	0.19(0.24 – 0.15)	-2.61(-3.19 – -2.02)
Micronesia (Federated States of)	Both	0.2(0.28 – 0.13)	0.32(0.46 – 0.21)	0.12	0.43(0.61 – 0.29)	0.44(0.62 – 0.29)	0.09(0.06 – 0.13)
Monaco	Both	1.26(1.85 – 0.76)	1.49(3.68 – 0.66)	0.23	1.66(2.43 – 1)	1.37(3.39 – 0.61)	-0.63(-0.66 – -0.6)

Mongolia	Both	4.25(6.14 – 2.64)	6.39(9.2 – 4.51)	2.14	0.42(0.61 – 0.26)	0.3(0.43 – 0.21)	-1.18(-1.73 – -0.63)
Montenegro	Both	8.28(11.01 – 6.07)	14.99(20.34 – 10.8)	6.70	1.35(1.8 – 0.99)	1.49(2.03 – 1.07)	0.49(0.15 – 0.83)
Morocco	Both	40.67(54.65 – 26.61)	80.75(114.97 – 55.3)	40.08	0.31(0.42 – 0.2)	0.25(0.36 – 0.17)	-0.73(-0.8 – -0.66)
Mozambique	Both	12.96(18.73 – 8.64)	23.61(33.64 – 17.05)	10.65	0.28(0.4 – 0.19)	0.27(0.38 – 0.19)	-0.14(-0.23 – -0.04)
Myanmar	Both	112.65(155.66 – 83.18)	121.64(199.27 – 82.67)	9.00	0.58(0.8 – 0.43)	0.29(0.49 – 0.2)	-2.18(-2.24 – -2.12)
Namibia	Both	1.47(1.91 – 1.1)	2.85(3.74 – 2.17)	1.38	0.31(0.41 – 0.23)	0.27(0.36 – 0.21)	-0.43(-0.56 – -0.3)
Nauru	Both	0.02(0.03 – 0.01)	0.03(0.04 – 0.02)	＜0.01	0.57(0.81 – 0.35)	0.48(0.7 – 0.29)	-0.54(-0.57 – -0.5)
Nepal	Both	29.23(46.09 – 18.05)	55.53(110.25 – 35.78)	26.31	0.42(0.66 – 0.25)	0.29(0.57 – 0.19)	-1.14(-1.27 – -1)
Netherlands	Both	499.26(568.92 – 422.55)	449.32(551.81 – 368.16)	-49.94	2.41(2.74 – 2.04)	1.16(1.42 – 0.95)	-2.34(-2.88 – -1.81)
New Zealand	Both	45.89(54.73 – 37.9)	58.07(73.97 – 44.58)	12.18	1.13(1.35 – 0.94)	0.64(0.81 – 0.49)	-1.85(-2.59 – -1.1)
Nicaragua	Both	1.35(1.74 – 0.98)	3.84(5.15 – 2.76)	2.49	0.1(0.13 – 0.07)	0.09(0.12 – 0.06)	-0.51(-0.85 – -0.17)
Niger	Both	2.02(3.19 – 1.29)	4.39(7.72 – 2.57)	2.37	0.11(0.17 – 0.06)	0.08(0.15 – 0.05)	-0.72(-0.87 – -0.57)
Nigeria	Both	12.62(19.06 – 8.6)	16.51(26.6 – 11.04)	3.89	0.03(0.05 – 0.02)	0.02(0.04 – 0.01)	-1.52(-1.71 – -1.32)
Niue	Both	0.01(0.01 – 0)	0.01(0.01 – 0)	＜0.01	0.3(0.4 – 0.2)	0.33(0.47 – 0.22)	0.36(0.29 – 0.42)
North Macedonia	Both	31.76(39.9 – 24.79)	56.14(73.81 – 40.41)	24.38	1.8(2.27 – 1.4)	1.75(2.29 – 1.28)	-0.12(-0.5 – 0.26)
Northern Mariana Islands	Both	0.05(0.08 – 0.03)	0.24(0.31 – 0.18)	0.19	0.35(0.59 – 0.24)	0.49(0.64 – 0.38)	1.05(0.72 – 1.38)

Norway	Both	136.89(159.73 – 117.06)	72.92(91.76 – 54.64)	-63.97	1.85(2.15 – 1.59)	0.66(0.82 – 0.5)	-3.3(-3.52 – -3.09)
Oman	Both	1.79(2.59 – 1.14)	3.16(4.28 – 2.2)	1.37	0.31(0.45 – 0.2)	0.22(0.3 – 0.15)	-1.1(-1.93 – -0.27)
Pakistan	Both	757.82(996.65 – 559.49)	1217.35(1781.88 – 858.13)	459.52	1.55(2.06 – 1.13)	1.25(1.84 – 0.87)	-0.68(-0.72 – -0.64)
Palau	Both	0.01(0.02 – 0.01)	0.03(0.04 – 0.02)	0.01	0.15(0.21 – 0.11)	0.12(0.16 – 0.09)	-0.82(-0.96 – -0.67)
Palestine	Both	11.19(15.56 – 7.57)	23.23(31.33 – 17.37)	12.04	1.47(2.04 – 1)	1.11(1.53 – 0.83)	-0.9(-1.24 – -0.55)
Panama	Both	2.99(3.64 – 2.46)	6.39(8.65 – 4.58)	3.40	0.22(0.26 – 0.18)	0.14(0.19 – 0.1)	-1.25(-1.62 – -0.88)
Papua New Guinea	Both	2.9(4.52 – 1.52)	7.86(12.47 – 4.27)	4.95	0.18(0.28 – 0.1)	0.16(0.26 – 0.09)	-0.21(-0.38 – -0.03)
Paraguay	Both	9.39(12.11 – 7.16)	23.12(32.06 – 15.9)	13.74	0.47(0.6 – 0.36)	0.43(0.6 – 0.3)	-0.21(-0.62 – 0.21)
Peru	Both	16.75(23.22 – 11.48)	40.33(60.92 – 25.41)	23.58	0.16(0.22 – 0.11)	0.12(0.19 – 0.08)	-0.67(-1.58 – 0.24)
Philippines	Both	76.14(98.31 – 58.71)	189.11(270.76 – 143.79)	112.97	0.32(0.42 – 0.25)	0.25(0.37 – 0.19)	-0.75(-0.93 – -0.57)
Poland	Both	875.7(981.09 – 766.46)	1397.81(1680.63 – 1142.14)	522.11	1.97(2.21 – 1.72)	1.85(2.22 – 1.52)	-0.25(-0.39 – -0.11)
Portugal	Both	168.51(199.65 – 140.86)	189.1(234.71 – 147.41)	20.59	1.19(1.42 – 1)	0.74(0.9 – 0.59)	-1.52(-2.16 – -0.88)
Puerto Rico	Both	18.89(24.1 – 14.47)	27.56(37.14 – 19.84)	8.67	0.53(0.68 – 0.4)	0.35(0.47 – 0.26)	-1.33(-2.25 – -0.4)
Qatar	Both	0.85(1.2 – 0.59)	3.44(5.43 – 2.08)	2.59	1.12(1.57 – 0.8)	0.55(0.86 – 0.35)	-2.26(-3.32 – -1.2)
Republic of Korea	Both	291.53(357.86 – 219.7)	644.21(850.96 – 448.03)	352.68	1.22(1.51 – 0.92)	0.69(0.9 – 0.48)	-1.87(-2.15 – -1.59)

Republic of Moldova	Both	41.91(48.99 – 34.11)	61.41(72.23 – 50.6)	19.50	0.95(1.12 – 0.77)	1(1.17 – 0.82)	0.07(-1.67 – 1.83)
Romania	Both	330.87(387.63 – 279.46)	499.54(612.17 – 397.25)	168.66	1.17(1.37 – 0.98)	1.31(1.59 – 1.05)	0.36(-0.02 – 0.75)
Russian Federation	Both	1973.63(2213.89 – 1741.48)	2196.73(2587.76 – 1816.03)	223.10	1.07(1.2 – 0.94)	0.89(1.05 – 0.74)	-0.56(-1.02 – -0.09)
Rwanda	Both	14.96(20.78 – 10.66)	26.73(41.68 – 17.64)	11.77	0.68(0.96 – 0.49)	0.57(0.88 – 0.38)	-0.56(-0.64 – -0.49)
Saint Kitts and Nevis	Both	0.15(0.2 – 0.12)	0.18(0.24 – 0.13)	0.02	0.38(0.5 – 0.29)	0.3(0.42 – 0.22)	-0.78(-1.15 – -0.41)
Saint Lucia	Both	0.52(0.66 – 0.41)	0.94(1.26 – 0.69)	0.42	0.63(0.81 – 0.49)	0.4(0.53 – 0.29)	-1.42(-2.06 – -0.77)
Saint Vincent and the Grenadines	Both	0.23(0.3 – 0.18)	0.46(0.59 – 0.35)	0.22	0.33(0.41 – 0.26)	0.33(0.42 – 0.25)	-0.1(-0.62 – 0.42)
Samoa	Both	0.35(0.5 – 0.25)	0.5(0.7 – 0.34)	0.15	0.45(0.63 – 0.33)	0.38(0.53 – 0.26)	-0.55(-0.61 – -0.49)
San Marino	Both	0.89(1.18 – 0.65)	0.76(1.11 – 0.48)	-0.13	2.39(3.15 – 1.74)	0.91(1.36 – 0.57)	-3.49(-3.91 – -3.06)
Sao Tome and Principe	Both	0.12(0.16 – 0.08)	0.27(0.42 – 0.17)	0.15	0.19(0.26 – 0.13)	0.27(0.42 – 0.17)	1.22(0.92 – 1.52)
Saudi Arabia	Both	14.98(23.03 – 9.3)	44.57(83.38 – 27.52)	29.59	0.29(0.45 – 0.18)	0.27(0.49 – 0.17)	-0.19(-0.27 – -0.12)
Senegal	Both	7.61(10.28 – 5.22)	11.59(17.45 – 7.54)	3.98	0.25(0.33 – 0.17)	0.16(0.24 – 0.1)	-1.45(-1.68 – -1.22)
Serbia	Both	169.26(221.25 – 127.31)	248.24(336.07 – 175.64)	78.98	1.66(2.18 – 1.25)	1.44(1.93 – 1.03)	-0.36(-0.5 – -0.22)
Seychelles	Both	0.85(1.08 – 0.65)	1.11(1.45 – 0.87)	0.26	1.51(1.92 – 1.14)	1.07(1.39 – 0.84)	-1.32(-2.27 – -0.37)
Sierra Leone	Both	4.93(6.61 – 3.46)	5.21(7.55 – 3.68)	0.28	0.26(0.36 – 0.19)	0.16(0.23 – 0.11)	-1.65(-1.71 – -1.59)

Singapore	Both	10.32(12.24 – 8.37)	17.14(21.53 – 13.37)	6.82	0.52(0.62 – 0.41)	0.2(0.26 – 0.16)	-3.03(-4.01 – -2.05)
Slovakia	Both	93.25(121.24 – 72.44)	106.9(146.65 – 76.65)	13.66	1.54(2 – 1.2)	1.08(1.47 – 0.78)	-1.11(-1.41 – -0.82)
Slovenia	Both	25.86(31.03 – 21.12)	41.03(52.65 – 31.37)	15.18	1.04(1.25 – 0.86)	0.9(1.15 – 0.7)	-0.55(-1.41 – 0.32)
Solomon Islands	Both	0.5(0.77 – 0.24)	1.33(2.04 – 0.74)	0.83	0.41(0.63 – 0.21)	0.42(0.63 – 0.24)	0.06(-0.06 – 0.18)
Somalia	Both	4.72(8.09 – 2.8)	9.09(15.24 – 4.97)	4.37	0.27(0.46 – 0.16)	0.19(0.31 – 0.11)	-1.06(-1.15 – -0.98)
South Africa	Both	103.11(144.9 – 73.81)	147.81(179.74 – 116.17)	44.70	0.55(0.78 – 0.39)	0.34(0.42 – 0.27)	-1.53(-2.08 – -0.97)
South Sudan	Both	6.79(10.28 – 4.39)	6.98(10.77 – 4.31)	0.19	0.3(0.44 – 0.19)	0.24(0.36 – 0.15)	-0.74(-0.81 – -0.67)
Spain	Both	1552.05(1783.25 – 1323.23)	1455.63(1803.74 – 1158.86)	-96.42	2.74(3.15 – 2.35)	1.37(1.66 – 1.09)	-2.22(-2.47 – -1.97)
Sri Lanka	Both	26.28(35.14 – 19.68)	42.11(60.99 – 23.67)	15.83	0.31(0.42 – 0.23)	0.17(0.24 – 0.1)	-1.99(-2.69 – -1.29)
Sudan	Both	56.92(99.81 – 31.96)	90.95(132.75 – 61.25)	34.03	0.68(1.19 – 0.38)	0.55(0.8 – 0.37)	-0.71(-0.73 – -0.7)
Suriname	Both	0.98(1.28 – 0.73)	1.96(2.77 – 1.28)	0.99	0.41(0.55 – 0.3)	0.32(0.45 – 0.21)	-0.75(-1.49 – -0.01)
Sweden	Both	195.05(232.4 – 161.76)	192.16(240.44 – 145.16)	-2.89	1.18(1.4 – 0.99)	0.77(0.95 – 0.59)	-1.35(-1.6 – -1.09)
Switzerland	Both	102.02(120.31 – 84.99)	182.1(222.69 – 144.32)	80.08	0.93(1.09 – 0.77)	0.87(1.06 – 0.7)	-0.25(-1.17 – 0.68)
Syrian Arab Republic	Both	38.86(53.39 – 27.85)	82.98(119.26 – 54.81)	44.12	0.85(1.18 – 0.6)	0.76(1.1 – 0.5)	-0.38(-0.71 – -0.04)
Taiwan (Province of China)	Both	177.97(204.54 – 152.65)	381.99(450.69 – 312.82)	204.02	1.28(1.47 – 1.08)	0.87(1.02 – 0.71)	-1.08(-1.38 – -0.78)

Tajikistan	Both	7.98(13.11 – 5.15)	8.14(12.44 – 5.17)	0.16	0.31(0.52 – 0.2)	0.16(0.24 – 0.11)	-2.12(-2.6 – -1.63)
Thailand	Both	299.65(381.5 – 212.49)	638.89(889.63 – 456.15)	339.24	1.03(1.33 – 0.73)	0.59(0.81 – 0.42)	-1.85(-2.11 – -1.59)
Timor-Leste	Both	0.57(0.91 – 0.34)	1.92(3.1 – 1.24)	1.35	0.28(0.44 – 0.17)	0.26(0.43 – 0.17)	-0.24(-0.35 – -0.14)
Togo	Both	4.52(6.06 – 3.23)	9.02(12.85 – 6.19)	4.50	0.46(0.61 – 0.33)	0.28(0.4 – 0.19)	-1.57(-1.7 – -1.44)
Tokelau	Both	0(0.01 – 0)	0(0.01 – 0)	＜0.01	0.36(0.52 – 0.23)	0.3(0.43 – 0.19)	-0.54(-0.71 – -0.37)
Tonga	Both	0.22(0.36 – 0.13)	0.34(0.54 – 0.21)	0.12	0.44(0.73 – 0.27)	0.44(0.7 – 0.27)	-0.07(-0.34 – 0.21)
Trinidad and Tobago	Both	3.45(4.07 – 2.84)	5.82(7.77 – 4.04)	2.37	0.43(0.51 – 0.36)	0.3(0.4 – 0.2)	-1.01(-1.37 – -0.65)
Tunisia	Both	64.67(90.45 – 45.25)	165.29(235.18 – 102.54)	100.62	1.53(2.1 – 1.06)	1.35(1.91 – 0.84)	-0.45(-0.58 – -0.33)
Turkey	Both	615.95(840.23 – 436.52)	1172.3(1598.8 – 851.58)	556.36	1.94(2.65 – 1.39)	1.29(1.76 – 0.94)	-1.32(-1.72 – -0.91)
Turkmenistan	Both	7.56(9 – 6.32)	10.37(14.14 – 7.39)	2.81	0.41(0.49 – 0.34)	0.26(0.36 – 0.19)	-1.46(-2.3 – -0.62)
Tuvalu	Both	0.02(0.03 – 0.02)	0.04(0.05 – 0.02)	0.01	0.35(0.48 – 0.24)	0.36(0.52 – 0.24)	0.16(0.12 – 0.19)
Uganda	Both	12.59(17.12 – 8.8)	23.1(32.03 – 16.91)	10.51	0.24(0.32 – 0.17)	0.2(0.27 – 0.14)	-0.7(-0.84 – -0.55)
Ukraine	Both	765.09(1016.57 – 595.01)	696.75(972.44 – 454.5)	-68.34	1.04(1.37 – 0.82)	0.87(1.22 – 0.57)	-0.51(-0.98 – -0.03)
United Arab Emirates	Both	4.1(7.82 – 2.19)	17.48(24.5 – 12.15)	13.38	1.16(2.25 – 0.62)	0.83(1.16 – 0.58)	-0.88(-1.86 – 0.12)
United Kingdom	Both	2495.44(2843.68 – 2154.75)	1633.23(2023.47 – 1287.55)	-862.21	2.59(2.95 – 2.24)	1.12(1.37 – 0.89)	-2.64(-2.77 – -2.5)

United Republic of Tanzania	Both	37.05(52.95 – 26.42)	54.54(79.73 – 36.99)	17.49	0.42(0.59 – 0.3)	0.26(0.38 – 0.17)	-1.56(-1.69 – -1.43)
United States of America	Both	3586.65(4216.83 – 3037.22)	4957.11(6195.55 – 3947.98)	1370.45	1.08(1.27 – 0.92)	0.8(1 – 0.64)	-1.78(-1.25 – -0.73)
United States Virgin Islands	Both	0.21(0.3 – 0.15)	0.31(0.46 – 0.19)	0.10	0.27(0.39 – 0.19)	0.16(0.23 – 0.1)	-0.99(-2.42 – -1.13)
Uruguay	Both	65.53(77.37 – 54.31)	72.55(88.94 – 58.25)	7.02	1.63(1.92 – 1.36)	1.26(1.52 – 1.02)	-0.9(-1.49 – -0.3)
Uzbekistan	Both	17.7(25.09 – 11.74)	59.44(79.41 – 43.01)	41.74	0.16(0.23 – 0.1)	0.24(0.33 – 0.18)	1.29(0.74 – 1.85)
Vanuatu	Both	0.16(0.24 – 0.09)	0.36(0.55 – 0.22)	0.20	0.29(0.43 – 0.17)	0.23(0.34 – 0.14)	-0.75(-0.93 – -0.56)
Venezuela (Bolivarian Republic of)	Both	34.31(40.38 – 28.06)	73.68(101.29 – 52.48)	39.37	0.39(0.47 – 0.32)	0.26(0.36 – 0.18)	-1.4(-2.35 – -0.44)
Viet Nam	Both	137.6(182.93 – 93.83)	350.92(446.82 – 265.7)	213.33	0.37(0.49 – 0.25)	0.39(0.5 – 0.3)	0.18(0.09 – 0.28)
Yemen	Both	36.19(56.98 – 20.76)	102.05(141.22 – 68.73)	65.86	0.81(1.27 – 0.47)	0.85(1.18 – 0.58)	0.12(0.03 – 0.22)
Zambia	Both	7.48(9.99 – 5.67)	17.39(31.74 – 9.97)	9.91	0.33(0.44 – 0.25)	0.32(0.55 – 0.19)	-0.05(-0.26 – 0.16)
Zimbabwe	Both	50.77(66.76 – 37.51)	83.73(113.98 – 62.01)	32.96	1.54(2.04 – 1.13)	1.53(2.04 – 1.13)	0.05(-0.1 – 0.19)
Afghanistan	Female	1.71(3.39 – 0.63)	2.96(5.37 – 1.3)	1.25	0.06(0.11 – 0.02)	0.06(0.11 – 0.03)	0.41(0.33 – 0.49)
Albania	Female	0.16(0.23 – 0.1)	0.45(0.69 – 0.29)	0.29	0.02(0.02 – 0.01)	0.02(0.03 – 0.01)	0.58(0.24 – 0.91)
Algeria	Female	1.4(2.27 – 0.85)	2.61(4.69 – 1.36)	1.21	0.05(0.09 – 0.02)	0.03(0.05 – 0.01)	-1.84(-2.27 – -1.41)
American Samoa	Female	0(0.01 – 0)	0.03(0.05 – 0.02)	0.03	0.04(0.08 – 0.03)	0.13(0.18 – 0.08)	3.74(2.59 – 4.9)

Andorra	Female	0.01(0.02 – 0.01)	0.03(0.04 – 0.02)	0.01	0.05(0.07 – 0.03)	0.03(0.05 – 0.02)	-1.48(-1.71 – -1.24)
Angola	Female	0.63(0.99 – 0.38)	1.91(2.83 – 1.19)	1.29	0.04(0.06 – 0.02)	0.03(0.05 – 0.02)	-0.3(-0.55 – -0.04)
Antigua and Barbuda	Female	0.05(0.07 – 0.03)	0.08(0.11 – 0.06)	0.04	0.14(0.19 – 0.1)	0.15(0.21 – 0.11)	0.21(-0.87 – 1.31)
Argentina	Female	52.35(66.75 – 40.35)	63.55(82.03 – 49.4)	11.20	0.29(0.37 – 0.22)	0.2(0.25 – 0.15)	-0.98(-1.27 – -0.69)
Armenia	Female	0.38(0.55 – 0.25)	0.6(0.84 – 0.41)	0.23	0.02(0.03 – 0.02)	0.02(0.03 – 0.02)	0.09(-0.61 – 0.8)
Australia	Female	46.85(57.33 – 37.54)	47.14(65.55 – 31.99)	0.29	0.41(0.5 – 0.33)	0.17(0.23 – 0.12)	-2.71(-2.87 – -2.56)
Austria	Female	24.56(31.59 – 18.92)	22.67(31.13 – 16.39)	-1.89	0.32(0.4 – 0.25)	0.21(0.28 – 0.16)	-1.3(-1.52 – -1.08)
Azerbaijan	Female	0.13(0.21 – 0.08)	0.21(0.34 – 0.12)	0.08	0(0.01 – 0)	0(0.01 – 0)	-0.67(-1.16 – -0.18)
Bahamas	Female	0.05(0.07 – 0.03)	0.14(0.19 – 0.1)	0.09	0.06(0.09 – 0.04)	0.07(0.1 – 0.05)	0.37(-0.52 – 1.26)
Bahrain	Female	0.15(0.2 – 0.1)	0.37(0.59 – 0.23)	0.22	0.25(0.36 – 0.16)	0.15(0.26 – 0.09)	-1.58(-2.08 – -1.08)
Bangladesh	Female	4.7(7.06 – 3.14)	8.62(15.33 – 5.28)	3.91	0.03(0.04 – 0.02)	0.01(0.03 – 0.01)	-1.98(-2.51 – -1.44)
Barbados	Female	0.08(0.11 – 0.05)	0.11(0.15 – 0.07)	0.03	0.04(0.06 – 0.03)	0.04(0.05 – 0.02)	-0.46(-1.25 – 0.33)
Belarus	Female	2.64(3.71 – 1.84)	2.14(3.03 – 1.43)	-0.49	0.03(0.05 – 0.02)	0.02(0.03 – 0.01)	-1.31(-2.38 – -0.24)
Belgium	Female	55.74(70.22 – 43.28)	41.51(56.75 – 29.45)	-14.23	0.58(0.71 – 0.46)	0.29(0.38 – 0.22)	-2.09(-2.94 – -1.22)
Belize	Female	0.03(0.04 – 0.02)	0.09(0.13 – 0.07)	0.06	0.06(0.09 – 0.04)	0.07(0.09 – 0.05)	0.1(-0.5 – 0.7)
Benin	Female	0.2(0.31 – 0.12)	0.26(0.41 – 0.15)	0.05	0.02(0.03 – 0.01)	0.01(0.02 – 0.01)	-2.23(-2.37 – -2.1)

Bermuda	Female	0.1(0.15 – 0.07)	0.14(0.21 – 0.09)	0.03	0.3(0.42 – 0.2)	0.15(0.23 – 0.11)	-2.07(-3.05 – -1.08)
Bhutan	Female	0.04(0.06 – 0.02)	0.07(0.15 – 0.04)	0.03	0.03(0.06 – 0.02)	0.02(0.05 – 0.01)	-1.24(-1.39 – -1.09)
Bolivia (Plurinational State of)	Female	0.86(1.45 – 0.45)	2.1(3.46 – 1.21)	1.24	0.05(0.09 – 0.03)	0.05(0.08 – 0.03)	-0.49(-0.56 – -0.41)
Bosnia and Herzegovina	Female	5.29(7.12 – 4)	11.66(17 – 7.22)	6.38	0.24(0.33 – 0.18)	0.32(0.46 – 0.2)	0.93(0.55 – 1.31)
Botswana	Female	0.32(0.51 – 0.19)	0.6(0.89 – 0.39)	0.28	0.13(0.21 – 0.08)	0.09(0.13 – 0.06)	-1.26(-2.22 – -0.29)
Brazil	Female	139.31(170.56 – 110.71)	272.99(340.11 – 213.42)	133.67	0.32(0.4 – 0.26)	0.2(0.24 – 0.15)	-1.62(-1.89 – -1.35)
Brunei Darussalam	Female	0.14(0.21 – 0.09)	0.24(0.34 – 0.16)	0.10	0.35(0.52 – 0.23)	0.17(0.26 – 0.12)	-2.23(-2.49 – -1.98)
Bulgaria	Female	10.14(13 – 7.48)	17.67(22.84 – 13.13)	7.53	0.15(0.19 – 0.11)	0.24(0.31 – 0.19)	1.66(0.74 – 2.59)
Burkina Faso	Female	0.22(0.33 – 0.13)	0.26(0.43 – 0.15)	0.04	0.01(0.02 – 0.01)	0.01(0.01 – 0)	-2.12(-2.25 – -1.98)
Burundi	Female	0.6(0.94 – 0.35)	0.38(0.56 – 0.24)	-0.22	0.05(0.09 – 0.03)	0.02(0.03 – 0.01)	-3.13(-3.26 – -3)
Cabo Verde	Female	0(0 – 0)	0.01(0.02 – 0)	0.01	0(0 – 0)	0.01(0.01 – 0)	3.85(3.34 – 4.37)
Cambodia	Female	1.34(2.19 – 0.72)	2.41(3.71 – 1.53)	1.07	0.06(0.1 – 0.03)	0.04(0.06 – 0.02)	-1.48(-1.55 – -1.42)
Cameroon	Female	0.46(0.77 – 0.26)	0.72(1.16 – 0.38)	0.26	0.03(0.05 – 0.02)	0.02(0.03 – 0.01)	-1.94(-2.1 – -1.78)
Canada	Female	123.04(148.99 – 96.7)	138.65(182.52 – 101.5)	15.62	0.64(0.78 – 0.51)	0.31(0.41 – 0.24)	-2.18(-2.77 – -1.58)
Central African Republic	Female	0.14(0.26 – 0.07)	0.19(0.31 – 0.1)	0.04	0.03(0.05 – 0.02)	0.02(0.03 – 0.01)	-1.24(-1.35 – -1.13)
Chad	Female	0.39(0.59 – 0.23)	0.41(0.65 – 0.25)	0.03	0.03(0.05 – 0.02)	0.02(0.03 – 0.01)	-1.26(-1.42 – -1.11)

Chile	Female	11.04(14.12 – 8.58)	15.4(19.95 – 11.8)	4.36	0.2(0.26 – 0.16)	0.11(0.14 – 0.08)	-1.96(-2.33 – -1.6)
China	Female	581.29(763.98 – 425.08)	759.06(1045.08 – 513.64)	177.77	0.18(0.24 – 0.13)	0.07(0.1 – 0.05)	-2.92(-3.27 – -2.58)
Colombia	Female	9.23(12.08 – 6.79)	12.5(16.62 – 9.03)	3.27	0.11(0.14 – 0.08)	0.04(0.05 – 0.03)	-3.35(-3.9 – -2.79)
Comoros	Female	0.04(0.07 – 0.02)	0.09(0.14 – 0.05)	0.05	0.05(0.09 – 0.03)	0.04(0.07 – 0.02)	-0.66(-0.8 – -0.53)
Congo	Female	0.09(0.16 – 0.05)	0.18(0.26 – 0.1)	0.08	0.02(0.03 – 0.01)	0.01(0.02 – 0.01)	-0.78(-0.97 – -0.59)
Cook Islands	Female	0.02(0.03 – 0.01)	0.02(0.04 – 0.02)	0.01	0.29(0.44 – 0.19)	0.19(0.27 – 0.12)	-1.42(-2.01 – -0.83)
Costa Rica	Female	1.07(1.41 – 0.78)	1.97(2.68 – 1.36)	0.89	0.13(0.17 – 0.09)	0.06(0.09 – 0.04)	-2.14(-2.67 – -1.62)
Croatia	Female	16.34(21.3 – 12.31)	31.02(41.86 – 22.27)	14.68	0.46(0.6 – 0.34)	0.54(0.7 – 0.39)	-0.84(-1.15 – -0.52)
Cuba	Female	14.82(19.59 – 10.96)	24.96(33.75 – 17.37)	10.14	0.29(0.39 – 0.22)	0.23(0.3 – 0.16)	0.46(-0.97 – 1.91)
Cyprus	Female	1.09(1.58 – 0.72)	1.58(2.18 – 1.11)	0.50	0.27(0.41 – 0.17)	0.15(0.2 – 0.1)	-0.74(-1.05 – -0.43)
Czechia	Female	35.26(45.03 – 26.48)	49.95(67.33 – 36.55)	14.69	0.41(0.52 – 0.31)	0.38(0.52 – 0.29)	-1.89(-2.33 – -1.45)
C么te d'Ivoire	Female	0.54(0.83 – 0.35)	1.18(1.85 – 0.72)	0.64	0.03(0.05 – 0.02)	0.02(0.04 – 0.01)	-0.28(-0.51 – -0.06)
Democratic People's Republic of Korea	Female	4.88(8.14 – 2.77)	8.39(15.29 – 4.97)	3.51	0.06(0.1 – 0.03)	0.04(0.08 – 0.03)	-0.95(-0.99 – -0.91)
Democratic Republic of the Congo	Female	1.23(1.95 – 0.74)	2.34(3.52 – 1.45)	1.11	0.02(0.02 – 0.01)	0.01(0.02 – 0.01)	-0.78(-0.91 – -0.64)
Denmark	Female	56.85(67.97 – 46.89)	48.16(61.61 – 36.34)	-8.68	1.14(1.35 – 0.95)	0.66(0.83 – 0.51)	-1.72(-2.25 – -1.2)

Djibouti	Female	0.05(0.08 – 0.02)	0.18(0.31 – 0.1)	0.13	0.1(0.16 – 0.05)	0.08(0.14 – 0.04)	-0.43(-0.5 – -0.36)
Dominica	Female	0.05(0.08 – 0.04)	0.05(0.07 – 0.04)	＜0.01	0.15(0.21 – 0.1)	0.11(0.16 – 0.08)	-0.78(-0.9 – -0.65)
Dominican Republic	Female	2.97(4.09 – 2.15)	7.57(10.94 – 4.95)	4.60	0.21(0.29 – 0.14)	0.15(0.21 – 0.1)	-1.13(-1.69 – -0.56)
Ecuador	Female	1.21(1.63 – 0.87)	3.08(4.48 – 2.05)	1.87	0.05(0.07 – 0.03)	0.04(0.05 – 0.02)	-0.87(-2.64 – 0.93)
Egypt	Female	6.63(9.41 – 4.5)	5.31(8.78 – 3.28)	-1.32	0.08(0.13 – 0.05)	0.04(0.07 – 0.02)	-2.6(-2.9 – -2.3)
El Salvador	Female	0.4(0.56 – 0.29)	1.06(1.49 – 0.71)	0.66	0.03(0.04 – 0.02)	0.03(0.04 – 0.02)	0.45(0.32 – 0.59)
Equatorial Guinea	Female	0.02(0.03 – 0.01)	0.04(0.06 – 0.02)	0.02	0.02(0.03 – 0.01)	0.01(0.02 – 0.01)	-0.29(-0.65 – 0.07)
Eritrea	Female	0.05(0.09 – 0.02)	0.11(0.17 – 0.06)	0.06	0.01(0.02 – 0.01)	0.01(0.02 – 0)	-0.57(-0.62 – -0.51)
Estonia	Female	2.32(3.14 – 1.64)	2.56(3.47 – 1.84)	0.24	0.17(0.23 – 0.12)	0.14(0.19 – 0.11)	-0.7(-0.93 – -0.46)
Eswatini	Female	0.17(0.28 – 0.1)	0.25(0.43 – 0.13)	0.08	0.14(0.23 – 0.08)	0.1(0.16 – 0.05)	-1.04(-1.26 – -0.83)
Ethiopia	Female	0.97(1.71 – 0.45)	1.39(2.06 – 0.87)	0.43	0.01(0.02 – 0.01)	0.01(0.01 – 0)	-1.39(-1.56 – -1.22)
Fiji	Female	0.1(0.14 – 0.07)	0.19(0.28 – 0.11)	0.09	0.05(0.07 – 0.04)	0.05(0.07 – 0.03)	-0.53(-0.9 – -0.15)
Finland	Female	9.87(13.35 – 7.34)	8.45(11.6 – 5.89)	-1.42	0.22(0.29 – 0.17)	0.11(0.15 – 0.08)	-2.11(-2.28 – -1.93)
France	Female	215.64(280.38 – 159.33)	208.65(292.94 – 143.45)	-6.99	0.39(0.49 – 0.29)	0.23(0.3 – 0.17)	-1.69(-1.96 – -1.43)
Gabon	Female	0.06(0.09 – 0.03)	0.09(0.14 – 0.05)	0.03	0.02(0.03 – 0.01)	0.02(0.03 – 0.01)	-0.26(-0.47 – -0.05)
Gambia	Female	0.01(0.02 – 0.01)	0.02(0.03 – 0.01)	0.01	0.01(0.01 – 0)	0(0.01 – 0)	-1.39(-1.76 – -1.03)

Georgia	Female	2.3(3.41 – 1.43)	1.9(2.66 – 1.34)	-0.40	0.06(0.09 – 0.04)	0.05(0.07 – 0.04)	-0.16(-1.3 – 0.99)
Germany	Female	427.59(530.71 – 332.07)	317.27(427.05 – 225.61)	-110.32	0.5(0.61 – 0.4)	0.27(0.34 – 0.21)	-2.04(-2.49 – -1.59)
Ghana	Female	0.65(0.95 – 0.41)	1.53(2.39 – 0.94)	0.88	0.03(0.04 – 0.02)	0.02(0.03 – 0.01)	-0.87(-0.99 – -0.74)
Greece	Female	26.63(34.07 – 21.12)	55.85(75.86 – 39.79)	29.22	0.32(0.41 – 0.25)	0.37(0.47 – 0.27)	0.5(0.09 – 0.92)
Greenland	Female	0.14(0.18 – 0.1)	0.12(0.17 – 0.08)	-0.02	0.93(1.21 – 0.65)	0.4(0.6 – 0.28)	-2.64(-3.33 – -1.94)
Grenada	Female	0.04(0.06 – 0.03)	0.05(0.07 – 0.03)	＜0.01	0.1(0.13 – 0.07)	0.08(0.12 – 0.06)	-0.5(-1.1 – 0.11)
Guam	Female	0.01(0.01 – 0.01)	0.04(0.05 – 0.03)	0.03	0.02(0.04 – 0.02)	0.03(0.05 – 0.02)	1.02(-0.47 – 2.54)
Guatemala	Female	0.44(0.63 – 0.31)	1(1.4 – 0.7)	0.57	0.03(0.05 – 0.02)	0.02(0.03 – 0.01)	-2.29(-3.8 – -0.75)
Guinea	Female	0.31(0.48 – 0.2)	0.44(0.67 – 0.27)	0.12	0.02(0.03 – 0.01)	0.02(0.03 – 0.01)	-0.51(-0.63 – -0.39)
Guinea-Bissau	Female	0.02(0.03 – 0.01)	0.04(0.06 – 0.02)	0.02	0.01(0.02 – 0.01)	0.01(0.02 – 0.01)	0.08(-0.06 – 0.22)
Guyana	Female	0.09(0.13 – 0.07)	0.13(0.2 – 0.09)	0.04	0.05(0.07 – 0.04)	0.04(0.06 – 0.03)	-0.64(-1.13 – -0.14)
Haiti	Female	1.15(2.09 – 0.53)	1.76(3.28 – 0.9)	0.61	0.08(0.14 – 0.04)	0.06(0.1 – 0.03)	-1.02(-1.12 – -0.93)
Honduras	Female	0.14(0.19 – 0.1)	0.57(0.85 – 0.37)	0.43	0.01(0.02 – 0.01)	0.02(0.03 – 0.01)	0.83(0.3 – 1.37)
Hungary	Female	31.35(39.84 – 23.96)	46.3(60.74 – 34.95)	14.95	0.37(0.46 – 0.28)	0.42(0.54 – 0.32)	0.51(-0.11 – 1.13)
Iceland	Female	1.19(1.51 – 0.88)	0.99(1.39 – 0.67)	-0.19	0.71(0.89 – 0.53)	0.29(0.39 – 0.21)	-3.02(-3.55 – -2.48)
India	Female	68.86(95.19 – 48.67)	138.98(192.02 – 100.33)	70.13	0.04(0.05 – 0.03)	0.03(0.04 – 0.02)	-1.19(-1.92 – -0.45)

Indonesia	Female	8.84(13.94 – 5.05)	19.24(30.99 – 11)	10.40	0.02(0.04 – 0.01)	0.02(0.04 – 0.01)	-0.13(-0.26 – -0.01)
Iran (Islamic Republic of)	Female	4.07(5.75 – 2.78)	11.56(15.95 – 7.86)	7.49	0.04(0.06 – 0.03)	0.03(0.05 – 0.02)	-0.53(-0.71 – -0.36)
Iraq	Female	8.83(12.78 – 5.93)	21.03(30.05 – 14.18)	12.20	0.23(0.34 – 0.15)	0.21(0.3 – 0.14)	-0.22(-0.44 – 0)
Ireland	Female	19.37(23.35 – 16.35)	16.1(22.26 – 11.47)	-3.27	0.82(0.98 – 0.69)	0.35(0.47 – 0.25)	-2.61(-3.12 – -2.09)
Israel	Female	8.99(12.03 – 6.58)	13.75(20.27 – 9.21)	4.76	0.35(0.47 – 0.25)	0.18(0.26 – 0.13)	-2.16(-2.48 – -1.83)
Italy	Female	225.55(281.9 – 178.13)	217.72(305.95 – 148.55)	-7.83	0.42(0.52 – 0.33)	0.23(0.3 – 0.17)	-1.83(-1.98 – -1.69)
Jamaica	Female	1.55(2.17 – 1.11)	2.23(3.27 – 1.45)	0.68	0.15(0.21 – 0.11)	0.13(0.18 – 0.09)	-0.3(-0.74 – 0.14)
Japan	Female	135.83(170.74 – 105.13)	248.93(393.93 – 143.66)	113.10	0.14(0.18 – 0.11)	0.09(0.12 – 0.06)	-1.64(-1.87 – -1.4)
Jordan	Female	0.76(1.06 – 0.51)	2.54(3.82 – 1.54)	1.78	0.16(0.23 – 0.1)	0.11(0.17 – 0.07)	-1.09(-2.04 – -0.14)
Kazakhstan	Female	1.78(2.64 – 1.11)	1.37(1.87 – 0.97)	-0.41	0.02(0.03 – 0.01)	0.01(0.02 – 0.01)	-1.7(-2.33 – -1.05)
Kenya	Female	1.27(1.9 – 0.85)	2.27(3.36 – 1.49)	1.00	0.04(0.06 – 0.02)	0.02(0.04 – 0.02)	-1.51(-1.69 – -1.33)
Kiribati	Female	0.02(0.03 – 0.01)	0.05(0.07 – 0.03)	0.03	0.11(0.15 – 0.07)	0.13(0.17 – 0.07)	0.45(0.31 – 0.6)
Kuwait	Female	0.25(0.37 – 0.17)	0.57(0.89 – 0.35)	0.32	0.14(0.21 – 0.09)	0.06(0.1 – 0.03)	-2.48(-4.19 – -0.73)
Kyrgyzstan	Female	0.2(0.28 – 0.13)	0.4(0.58 – 0.27)	0.21	0.01(0.02 – 0.01)	0.01(0.02 – 0.01)	1(-1.07 – 3.11)
Lao People's Democratic Republic	Female	0.51(0.9 – 0.23)	0.76(1.22 – 0.45)	0.26	0.06(0.1 – 0.03)	0.04(0.06 – 0.02)	-1.19(-1.23 – -1.14)

Latvia	Female	2.22(2.99 – 1.58)	2.17(2.93 – 1.54)	-0.05	0.1(0.13 – 0.07)	0.09(0.12 – 0.07)	-0.47(-0.72 – -0.21)
Lebanon	Female	7.74(11.69 – 4.95)	24.34(35.35 – 15.94)	16.60	0.77(1.16 – 0.49)	0.7(1.01 – 0.47)	-0.28(-0.51 – -0.04)
Lesotho	Female	0.61(0.98 – 0.34)	0.88(1.45 – 0.48)	0.27	0.13(0.21 – 0.07)	0.17(0.28 – 0.09)	0.96(0.66 – 1.26)
Liberia	Female	0.09(0.13 – 0.05)	0.1(0.15 – 0.06)	0.02	0.02(0.03 – 0.01)	0.01(0.02 – 0.01)	-1.53(-1.71 – -1.34)
Libya	Female	0.23(0.34 – 0.14)	0.52(0.8 – 0.32)	0.30	0.03(0.04 – 0.02)	0.02(0.04 – 0.01)	-0.52(-0.81 – -0.24)
Lithuania	Female	1.49(1.97 – 1.11)	2.21(2.96 – 1.6)	0.72	0.05(0.07 – 0.04)	0.07(0.09 – 0.05)	0.61(-0.34 – 1.57)
Luxembourg	Female	1.66(2.22 – 1.19)	1.87(2.56 – 1.31)	0.22	0.5(0.66 – 0.36)	0.3(0.41 – 0.22)	-1.62(-2.11 – -1.13)
Madagascar	Female	0.85(1.31 – 0.53)	0.88(1.33 – 0.53)	0.03	0.05(0.08 – 0.03)	0.02(0.04 – 0.01)	-2.12(-2.21 – -2.03)
Malawi	Female	3.75(5.51 – 2.5)	6.53(10.79 – 3.76)	2.78	0.18(0.26 – 0.12)	0.17(0.27 – 0.1)	-0.25(-0.34 – -0.17)
Malaysia	Female	2.59(4.06 – 1.48)	5.62(8.72 – 3.41)	3.03	0.06(0.1 – 0.04)	0.05(0.09 – 0.03)	-0.59(-0.81 – -0.38)
Maldives	Female	0.01(0.02 – 0.01)	0.03(0.05 – 0.02)	0.02	0.05(0.08 – 0.02)	0.03(0.04 – 0.01)	-2.12(-2.38 – -1.87)
Mali	Female	0.76(1.09 – 0.53)	1.82(2.78 – 1.12)	1.06	0.04(0.07 – 0.03)	0.05(0.08 – 0.03)	0.31(0.19 – 0.43)
Malta	Female	0.99(1.33 – 0.72)	1.26(1.79 – 0.85)	0.27	0.42(0.56 – 0.3)	0.23(0.31 – 0.16)	-2.03(-2.66 – -1.4)
Marshall Islands	Female	0(0 – 0)	0.01(0.01 – 0)	0.01	0.04(0.06 – 0.02)	0.05(0.08 – 0.03)	1.07(1.02 – 1.12)
Mauritania	Female	0.11(0.17 – 0.06)	0.12(0.19 – 0.07)	0.01	0.02(0.03 – 0.01)	0.01(0.02 – 0.01)	-1.88(-2.04 – -1.71)
Mauritius	Female	0.44(0.6 – 0.31)	0.36(0.52 – 0.25)	-0.08	0.12(0.17 – 0.09)	0.04(0.05 – 0.02)	-3.96(-5.19 – -2.7)

Mexico	Female	22.6(30 – 16.74)	22.64(30.8 – 15.96)	0.04	0.13(0.18 – 0.09)	0.04(0.05 – 0.02)	-4.07(-5.3 – -2.82)
Micronesia (Federated States of)	Female	0.02(0.03 – 0.01)	0.05(0.07 – 0.02)	0.02	0.09(0.12 – 0.05)	0.11(0.17 – 0.06)	0.75(0.69 – 0.82)
Monaco	Female	0.21(0.37 – 0.11)	0.27(0.69 – 0.08)	0.06	0.48(0.84 – 0.25)	0.45(1.16 – 0.14)	-0.21(-0.24 – -0.18)
Mongolia	Female	0.51(0.79 – 0.28)	0.39(0.61 – 0.25)	-0.12	0.09(0.14 – 0.05)	0.03(0.05 – 0.02)	-3.34(-3.82 – -2.85)
Montenegro	Female	1.13(1.53 – 0.81)	1.88(2.57 – 1.36)	0.75	0.33(0.44 – 0.23)	0.34(0.46 – 0.25)	0.2(-0.13 – 0.53)
Morocco	Female	0.25(0.35 – 0.15)	0.41(0.63 – 0.25)	0.17	0(0.01 – 0)	0(0 – 0)	-1.24(-1.32 – -1.16)
Mozambique	Female	1.52(2.16 – 0.99)	2.32(3.53 – 1.37)	0.80	0.06(0.09 – 0.04)	0.05(0.07 – 0.03)	-0.71(-0.87 – -0.56)
Myanmar	Female	22.31(36.94 – 12.6)	15.36(26.07 – 10.04)	-6.95	0.21(0.35 – 0.12)	0.06(0.11 – 0.04)	-3.88(-4.05 – -3.72)
Namibia	Female	0.59(0.8 – 0.4)	1.01(1.49 – 0.64)	0.43	0.22(0.3 – 0.15)	0.16(0.24 – 0.1)	-0.94(-1.08 – -0.8)
Nauru	Female	0(0 – 0)	0(0.01 – 0)	＜0.01	0.12(0.19 – 0.07)	0.13(0.21 – 0.07)	0.34(0.27 – 0.41)
Nepal	Female	6.72(10.52 – 4.57)	9.35(17.61 – 6.02)	2.63	0.19(0.3 – 0.13)	0.09(0.17 – 0.06)	-2.32(-2.39 – -2.25)
Netherlands	Female	82.55(102.1 – 65.69)	88.4(113.62 – 65.09)	5.85	0.67(0.81 – 0.54)	0.41(0.53 – 0.31)	-1.58(-2.38 – -0.76)
New Zealand	Female	11.93(14.92 – 9.46)	11.93(15.71 – 8.54)	＜0.01	0.51(0.64 – 0.41)	0.24(0.31 – 0.17)	-2.5(-3.5 – -1.48)
Nicaragua	Female	0.11(0.14 – 0.08)	0.31(0.43 – 0.21)	0.21	0.01(0.02 – 0.01)	0.01(0.02 – 0.01)	-0.33(-0.57 – -0.08)
Niger	Female	0.06(0.1 – 0.04)	0.13(0.21 – 0.07)	0.07	0.01(0.01 – 0)	0(0.01 – 0)	-1.53(-1.63 – -1.44)

Nigeria	Female	2.55(4.26 – 1.51)	2.04(3.34 – 1.2)	-0.51	0.01(0.02 – 0.01)	0.01(0.01 – 0)	-3.16(-3.29 – -3.02)
Niue	Female	0(0 – 0)	0(0 – 0)	＜0.01	0.05(0.08 – 0.03)	0.06(0.09 – 0.03)	0.36(0.25 – 0.48)
North Macedonia	Female	2.81(3.77 – 2.1)	5.72(7.71 – 3.96)	2.90	0.3(0.39 – 0.22)	0.36(0.51 – 0.25)	0.65(0.33 – 0.97)
Northern Mariana Islands	Female	0(0.01 – 0)	0.05(0.07 – 0.02)	0.05	0.04(0.09 – 0.02)	0.19(0.27 – 0.09)	5.24(4.46 – 6.02)
Norway	Female	28.01(36.17 – 20.41)	14.33(21.42 – 9.42)	-13.68	0.64(0.8 – 0.49)	0.23(0.32 – 0.16)	-3.33(-3.81 – -2.85)
Oman	Female	0.13(0.2 – 0.08)	0.16(0.23 – 0.1)	0.03	0.05(0.07 – 0.03)	0.02(0.03 – 0.01)	-2.38(-2.77 – -1.99)
Pakistan	Female	33.22(47.35 – 23.87)	47.88(70.89 – 31.49)	14.66	0.15(0.21 – 0.1)	0.1(0.15 – 0.07)	-1.21(-1.31 – -1.11)
Palau	Female	0(0 – 0)	0(0 – 0)	＜0.01	0(0.01 – 0)	0(0.01 – 0)	-0.64(-0.72 – -0.57)
Palestine	Female	0.4(0.62 – 0.25)	0.5(0.79 – 0.3)	0.10	0.1(0.16 – 0.06)	0.05(0.09 – 0.03)	-2.29(-2.51 – -2.07)
Panama	Female	0.54(0.75 – 0.39)	1.05(1.54 – 0.69)	0.51	0.08(0.11 – 0.06)	0.04(0.06 – 0.03)	-1.78(-2.79 – -0.77)
Papua New Guinea	Female	0.54(0.81 – 0.3)	1.6(2.4 – 0.89)	1.06	0.06(0.09 – 0.03)	0.06(0.1 – 0.04)	0.31(0.05 – 0.56)
Paraguay	Female	1.17(1.58 – 0.8)	2.43(3.59 – 1.6)	1.26	0.11(0.15 – 0.07)	0.08(0.12 – 0.05)	-0.84(-1.25 – -0.42)
Peru	Female	1.32(1.87 – 0.89)	3.69(5.35 – 2.45)	2.37	0.02(0.03 – 0.02)	0.02(0.03 – 0.01)	-0.29(-1.65 – 1.08)
Philippines	Female	9.91(13.48 – 6.92)	18.71(25.5 – 13.69)	8.80	0.11(0.16 – 0.07)	0.05(0.07 – 0.04)	-2.29(-2.68 – -1.89)
Poland	Female	68.24(82.84 – 56.08)	197.64(246.27 – 152.68)	129.40	0.26(0.32 – 0.22)	0.46(0.56 – 0.36)	1.86(1.2 – 2.52)
Portugal	Female	12.97(16.88 – 9.82)	8.39(11.38 – 6.17)	-4.59	0.16(0.2 – 0.12)	0.07(0.09 – 0.05)	-2.69(-3.16 – -2.21)

Puerto Rico	Female	3.83(5.33－2.63)	4.8(7.41－3.13)	0.98	0.2(0.28－0.14)	0.1(0.15－0.07)	-2.05(-2.4－-1.7)
Qatar	Female	0.01(0.02－0.01)	0.05(0.09－0.03)	0.04	0.06(0.1－0.03)	0.03(0.05－0.02)	-2.07(-2.95－-1.19)
Republic of Korea	Female	26.96(38.49－18.04)	67.23(108.96－38.36)	40.27	0.22(0.32－0.14)	0.11(0.18－0.07)	-2.13(-2.46－-1.81)
Republic of Moldova	Female	0.92(1.4－0.6)	0.98(1.38－0.68)	0.05	0.03(0.05－0.02)	0.03(0.04－0.02)	-1.01(-2.2－0.2)
Romania	Female	24.66(33.57－17.77)	32.59(44.09－23.4)	7.93	0.16(0.21－0.12)	0.16(0.21－0.12)	0.1(-0.27－0.47)
Russian Federation	Female	37.93(49.04－28.78)	63(83.17－48.59)	25.07	0.03(0.04－0.02)	0.04(0.05－0.03)	0.81(0－1.62)
Rwanda	Female	4.15(6.49－2.54)	9.1(14.65－4.75)	4.95	0.35(0.55－0.22)	0.33(0.54－0.17)	-0.22(-0.3－-0.14)
Saint Kitts and Nevis	Female	0.04(0.06－0.02)	0.04(0.05－0.02)	＜0.01	0.17(0.24－0.11)	0.12(0.17－0.08)	-1.01(-2.34－0.33)
Saint Lucia	Female	0.09(0.13－0.07)	0.15(0.22－0.1)	0.06	0.2(0.28－0.14)	0.12(0.17－0.08)	-2.07(-2.78－-1.36)
Saint Vincent and the Grenadines	Female	0.03(0.04－0.02)	0.04(0.05－0.03)	0.01	0.08(0.11－0.05)	0.05(0.08－0.04)	-1.25(-2.25－-0.24)
Samoa	Female	0(0－0)	0(0－0)	＜0.01	0(0.01－0)	0(0.01－0)	-0.06(-0.16－0.05)
San Marino	Female	0.1(0.15－0.07)	0.09(0.14－0.05)	-0.01	0.49(0.71－0.33)	0.2(0.31－0.11)	-3.17(-3.57－-2.77)
Sao Tome and Principe	Female	0(0.01－0)	0.01(0.01－0)	＜0.01	0.01(0.02－0.01)	0.01(0.02－0.01)	0.22(0.02－0.42)
Saudi Arabia	Female	0.52(0.86－0.32)	1.36(2.24－0.79)	0.84	0.02(0.04－0.01)	0.02(0.03－0.01)	-0.61(-0.81－-0.4)
Senegal	Female	0.16(0.24－0.1)	0.2(0.3－0.12)	0.04	0.01(0.02－0.01)	0.01(0.01－0)	-2.34(-2.57－-2.12)

Serbia	Female	18.91(24.95 – 14.12)	32.08(42.81 – 23.11)	13.17	0.33(0.44 – 0.25)	0.35(0.46 – 0.25)	0.17(-0.16 – 0.51)
Seychelles	Female	0.05(0.08 – 0.03)	0.06(0.1 – 0.04)	0.01	0.15(0.24 – 0.09)	0.11(0.17 – 0.07)	-1.02(-1.55 – -0.49)
Sierra Leone	Female	0.16(0.23 – 0.1)	0.19(0.28 – 0.13)	0.03	0.02(0.02 – 0.01)	0.01(0.02 – 0.01)	-1.55(-1.79 – -1.32)
Singapore	Female	1.1(1.6 – 0.72)	2.16(3.61 – 1.22)	1.06	0.11(0.16 – 0.07)	0.05(0.08 – 0.03)	-2.67(-3.65 – -1.67)
Slovakia	Female	4.66(6.31 – 3.45)	7.22(9.8 – 5.04)	2.56	0.14(0.19 – 0.1)	0.13(0.18 – 0.1)	-0.13(-0.47 – 0.21)
Slovenia	Female	3.73(4.73 – 2.85)	7.11(9.44 – 5.09)	3.38	0.25(0.32 – 0.19)	0.29(0.38 – 0.21)	0.38(-0.32 – 1.09)
Solomon Islands	Female	0.04(0.06 – 0.02)	0.14(0.21 – 0.08)	0.11	0.06(0.1 – 0.03)	0.08(0.12 – 0.05)	1.05(0.84 – 1.26)
Somalia	Female	0.49(0.86 – 0.25)	1.22(2.11 – 0.61)	0.73	0.05(0.1 – 0.03)	0.05(0.08 – 0.02)	-0.44(-0.54 – -0.35)
South Africa	Female	18.25(25.22 – 13)	21.2(28.78 – 15.39)	2.95	0.16(0.23 – 0.12)	0.08(0.12 – 0.06)	-2.18(-2.37 – -2)
South Sudan	Female	0.43(0.8 – 0.22)	0.53(0.88 – 0.27)	0.10	0.05(0.09 – 0.02)	0.04(0.07 – 0.02)	-0.69(-0.76 – -0.63)
Spain	Female	68.75(92.26 – 51.07)	65.19(89.23 – 45.96)	-3.55	0.22(0.29 – 0.16)	0.12(0.15 – 0.09)	-1.72(-1.99 – -1.45)
Sri Lanka	Female	0.73(1.11 – 0.47)	1.4(2.21 – 0.77)	0.67	0.02(0.03 – 0.01)	0.01(0.02 – 0.01)	-2.09(-2.59 – -1.6)
Sudan	Female	2.01(3.45 – 1.03)	2.45(3.97 – 1.4)	0.44	0.05(0.09 – 0.03)	0.03(0.06 – 0.02)	-1.45(-1.5 – -1.41)
Suriname	Female	0.11(0.15 – 0.08)	0.21(0.3 – 0.14)	0.10	0.09(0.12 – 0.06)	0.06(0.09 – 0.04)	-1.08(-1.41 – -0.75)
Sweden	Female	48.86(62.9 – 37.22)	53.09(71.8 – 38.84)	4.23	0.51(0.64 – 0.4)	0.39(0.52 – 0.29)	-0.82(-1.13 – -0.5)
Switzerland	Female	18.23(24.19 – 13.53)	35.66(50.17 – 24.54)	17.44	0.28(0.36 – 0.21)	0.3(0.4 – 0.22)	0.19(-0.86 – 1.25)

Syrian Arab Republic	Female	1.05(1.53 – 0.7)	1.58(2.4 – 0.96)	0.53	0.05(0.07 – 0.03)	0.03(0.05 – 0.02)	-1.4(-2 – -0.8)
Taiwan (Province of China)	Female	4.98(6.98 – 3.47)	6.5(9.46 – 4.2)	1.53	0.07(0.1 – 0.05)	0.03(0.04 – 0.02)	-2.8(-3.6 – -1.99)
Tajikistan	Female	0.11(0.17 – 0.07)	0.11(0.17 – 0.07)	＜0.01	0.01(0.01 – 0)	0(0.01 – 0)	-2.17(-2.53 – -1.8)
Thailand	Female	18.73(27.4 – 13.08)	24.5(41 – 14.9)	5.78	0.12(0.18 – 0.08)	0.04(0.07 – 0.02)	-3.59(-3.88 – -3.29)
Timor-Leste	Female	0.03(0.05 – 0.01)	0.06(0.11 – 0.04)	0.04	0.03(0.05 – 0.01)	0.02(0.03 – 0.01)	-1.62(-1.75 – -1.49)
Togo	Female	0.31(0.48 – 0.18)	0.59(0.94 – 0.33)	0.28	0.06(0.09 – 0.03)	0.03(0.05 – 0.02)	-2.04(-2.15 – -1.94)
Tokelau	Female	0(0 – 0)	0(0 – 0)	＜0.01	0.06(0.09 – 0.04)	0.06(0.09 – 0.03)	-0.09(-0.18 – -0.01)
Tonga	Female	0.01(0.02 – 0.01)	0.02(0.04 – 0.01)	0.01	0.06(0.09 – 0.03)	0.05(0.09 – 0.03)	-0.27(-0.38 – -0.16)
Trinidad and Tobago	Female	0.48(0.65 – 0.36)	0.68(0.99 – 0.46)	0.20	0.12(0.16 – 0.09)	0.07(0.1 – 0.04)	-1.97(-2.4 – -1.54)
Tunisia	Female	1.15(1.67 – 0.74)	2.42(3.94 – 1.41)	1.28	0.06(0.09 – 0.03)	0.04(0.06 – 0.02)	-1.47(-1.55 – -1.39)
Turkey	Female	18.42(26.31 – 12.26)	29.17(42.45 – 20.32)	10.76	0.1(0.15 – 0.07)	0.06(0.09 – 0.04)	-1.82(-2.12 – -1.52)
Turkmenistan	Female	0.27(0.36 – 0.19)	0.19(0.27 – 0.13)	-0.08	0.02(0.03 – 0.02)	0.01(0.01 – 0.01)	-3.73(-4.83 – -2.61)
Tuvalu	Female	0(0 – 0)	0(0.01 – 0)	＜0.01	0.08(0.12 – 0.05)	0.08(0.13 – 0.05)	0.33(0.25 – 0.41)
Uganda	Female	2.39(3.58 – 1.42)	5.15(7.67 – 3.47)	2.76	0.09(0.15 – 0.05)	0.08(0.12 – 0.05)	-0.56(-0.77 – -0.36)
Ukraine	Female	10.02(14.5 – 6.75)	10.29(15.44 – 6.25)	0.27	0.02(0.03 – 0.02)	0.02(0.03 – 0.01)	-0.04(-1.04 – 0.97)
United Arab Emirates	Female	0.17(0.35 – 0.08)	0.49(0.73 – 0.33)	0.31	0.14(0.27 – 0.07)	0.16(0.25 – 0.1)	0.58(-0.96 – 2.14)

United Kingdom	Female	629.86(737.29 – 526.99)	481.99(617.14 – 368.9)	-147.87	1.09(1.27 – 0.92)	0.59(0.75 – 0.47)	-1.96(-2.15 – -1.76)
United Republic of Tanzania	Female	5.46(7.85 – 3.76)	9.35(14.06 – 6.04)	3.88	0.13(0.2 – 0.09)	0.08(0.13 – 0.05)	-1.44(-1.57 – -1.31)
United States of America	Female	911.73(1096.75 – 749.6)	1161.55(1483.9 – 876.94)	249.81	0.46(0.55 – 0.39)	0.34(0.42 – 0.26)	-1.12(-1.26 – -0.98)
United States Virgin Islands	Female	0.03(0.05 – 0.02)	0.04(0.06 – 0.02)	0.01	0.08(0.12 – 0.05)	0.03(0.06 – 0.02)	-2.63(-3 – -2.25)
Uruguay	Female	5.13(6.76 – 3.8)	7.21(9.4 – 5.43)	2.08	0.23(0.29 – 0.17)	0.23(0.28 – 0.18)	-0.02(-0.38 – 0.35)
Uzbekistan	Female	0.19(0.28 – 0.11)	0.68(0.96 – 0.47)	0.49	0(0 – 0)	0(0.01 – 0)	1.44(0.94 – 1.95)
Vanuatu	Female	0.01(0.01 – 0)	0.02(0.02 – 0.01)	0.01	0.02(0.04 – 0.01)	0.02(0.03 – 0.01)	-0.3(-0.58 – -0.02)
Venezuela (Bolivarian Republic of)	Female	5.38(6.9 – 3.93)	11.24(15.93 – 7.41)	5.86	0.11(0.15 – 0.08)	0.07(0.1 – 0.04)	-1.79(-2.08 – -1.5)
Viet Nam	Female	4.78(6.73 – 3.23)	5.36(8.02 – 3.45)	0.58	0.02(0.03 – 0.01)	0.01(0.02 – 0.01)	-2.32(-2.42 – -2.23)
Yemen	Female	3.27(5.27 – 1.83)	7.87(12.21 – 4.75)	4.60	0.15(0.24 – 0.08)	0.13(0.21 – 0.08)	-0.39(-0.46 – -0.32)
Zambia	Female	2.02(2.81 – 1.33)	4.15(7.81 – 1.91)	2.14	0.21(0.31 – 0.13)	0.17(0.31 – 0.08)	-0.79(-0.92 – -0.65)
Zimbabwe	Female	10.03(14.89 – 6.1)	13.33(22 – 7.13)	3.30	0.65(0.98 – 0.38)	0.52(0.84 – 0.27)	-0.64(-1.07 – -0.22)
Afghanistan	Male	25.48(48.28 – 12.62)	35.4(54.88 – 20.12)	9.93	0.74(1.39 – 0.37)	0.97(1.49 – 0.56)	0.88(0.8 – 0.96)
Albania	Male	2.19(3.01 – 1.59)	4.97(7.4 – 3.3)	2.78	0.31(0.42 – 0.22)	0.25(0.37 – 0.16)	-0.67(-1.35 – 0.01)
Algeria	Male	58.75(78.07 – 39.32)	137.89(190.28 – 95.94)	79.14	1.29(1.7 – 0.84)	0.96(1.31 – 0.66)	-0.96(-1.04 – -0.88)

American Samoa	Male	0.06(0.08 - 0.05)	0.14(0.18 - 0.1)	0.08	0.65(0.89 - 0.47)	0.61(0.81 - 0.47)	-0.09(-0.39 - 0.21)
Andorra	Male	1.26(1.84 - 0.83)	1.8(2.61 - 1.12)	0.53	4.66(6.71 - 3.1)	2.32(3.37 - 1.44)	-2.34(-2.62 - -2.05)
Angola	Male	12.98(19.39 - 8.68)	33.86(47.88 - 24)	20.88	0.86(1.27 - 0.59)	0.85(1.19 - 0.59)	-0.02(-0.22 - 0.18)
Antigua and Barbuda	Male	0.16(0.21 - 0.13)	0.27(0.35 - 0.2)	0.11	0.7(0.9 - 0.53)	0.59(0.76 - 0.43)	-0.51(-1.82 - 0.82)
Argentina	Male	354.54(422 - 288.34)	301.77(371.05 - 234.58)	-52.77	2.5(2.99 - 2.01)	1.23(1.52 - 0.95)	-2.24(-2.8 - -1.67)
Armenia	Male	56.02(67.8 - 46.01)	100.7(123.95 - 81.11)	44.68	5.44(6.6 - 4.44)	5.64(6.95 - 4.54)	0.05(-0.78 - 0.89)
Australia	Male	147.78(177.4 - 122.88)	130.19(171.3 - 98.44)	-17.58	1.72(2.09 - 1.41)	0.59(0.77 - 0.45)	-3.5(-3.64 - -3.35)
Austria	Male	134.19(159.01 - 112.47)	135.89(166.58 - 107.6)	1.69	3.04(3.6 - 2.54)	1.62(1.98 - 1.29)	-1.84(-2.17 - -1.5)
Azerbaijan	Male	35.11(48.99 - 25.92)	65(91.81 - 44.58)	29.90	2.01(2.74 - 1.51)	1.67(2.33 - 1.19)	-0.62(-0.92 - -0.32)
Bahamas	Male	0.3(0.39 - 0.23)	0.79(1.02 - 0.55)	0.49	0.49(0.63 - 0.37)	0.48(0.63 - 0.34)	-0.07(-0.78 - 0.65)
Bahrain	Male	2.98(3.96 - 2.16)	7.65(11.45 - 5.03)	4.67	4.98(6.75 - 3.6)	2.68(3.88 - 1.79)	-1.9(-2.7 - -1.1)
Bangladesh	Male	205.24(329.76 - 121.65)	413.42(853.11 - 233.5)	208.18	0.89(1.44 - 0.53)	0.66(1.36 - 0.38)	-0.86(-1.22 - -0.49)
Barbados	Male	1.12(1.37 - 0.87)	1.52(2.07 - 1.05)	0.40	0.88(1.07 - 0.68)	0.66(0.9 - 0.45)	-0.87(-1.41 - -0.32)
Belarus	Male	168.99(193.01 - 146.03)	159.47(195.48 - 124.64)	-9.52	3.94(4.52 - 3.38)	2.84(3.48 - 2.25)	-0.99(-1.6 - -0.38)
Belgium	Male	317.88(369.28 - 267.34)	225.79(282.91 - 179.48)	-92.09	5.28(6.16 - 4.4)	2.03(2.52 - 1.63)	-3.12(-4.1 - -2.13)
Belize	Male	0.22(0.27 - 0.18)	0.67(0.84 - 0.51)	0.45	0.51(0.62 - 0.4)	0.5(0.63 - 0.37)	0.17(-0.58 - 0.92)

Benin	Male	3.72(5.48 – 2.59)	4.38(6.29 – 2.99)	0.66	0.42(0.61 – 0.29)	0.22(0.31 – 0.15)	-2.04(-2.27 – -1.8)
Bermuda	Male	0.61(0.79 – 0.47)	1.01(1.32 – 0.73)	0.40	2.5(3.25 – 1.88)	1.62(2.13 – 1.17)	-1.57(-2.16 – -0.97)
Bhutan	Male	0.35(0.62 – 0.17)	0.97(2.26 – 0.53)	0.62	0.44(0.79 – 0.21)	0.36(0.84 – 0.2)	-0.64(-0.74 – -0.53)
Bolivia (Plurinational State of)	Male	8.59(12.72 – 5.71)	18.54(27.15 – 11.97)	9.94	0.69(1.03 – 0.46)	0.51(0.75 – 0.32)	-0.98(-1.04 – -0.92)
Bosnia and Herzegovina	Male	38.12(48.11 – 30.57)	89.4(120.12 – 62.8)	51.28	2.45(3.08 – 1.96)	3.23(4.36 – 2.25)	0.94(0.57 – 1.32)
Botswana	Male	2.26(3.1 – 1.55)	4.56(6.54 – 3.09)	2.30	1.18(1.63 – 0.82)	0.97(1.35 – 0.67)	-0.6(-0.85 – -0.35)
Brazil	Male	615.71(698.59 – 536.5)	962.23(1179.68 – 777.77)	346.52	1.74(2 – 1.51)	0.93(1.15 – 0.75)	-1.91(-2.25 – -1.57)
Brunei Darussalam	Male	0.77(1.07 – 0.55)	1.19(1.58 – 0.85)	0.42	1.87(2.59 – 1.31)	1.05(1.42 – 0.72)	-1.87(-2.52 – -1.21)
Bulgaria	Male	149.14(177.56 – 124.11)	188.88(239.31 – 149.12)	39.74	2.57(3.07 – 2.14)	3.01(3.83 – 2.37)	0.51(-0.03 – 1.06)
Burkina Faso	Male	3.96(5.74 – 2.63)	6.9(9.94 – 4.84)	2.94	0.21(0.31 – 0.14)	0.18(0.26 – 0.12)	-0.57(-0.74 – -0.4)
Burundi	Male	7.19(11.29 – 4.36)	6.33(9.52 – 4.08)	-0.86	0.78(1.2 – 0.47)	0.32(0.47 – 0.2)	-2.88(-2.95 – -2.81)
Cabo Verde	Male	0.06(0.17 – 0.03)	0.66(0.99 – 0.33)	0.61	0.06(0.18 – 0.03)	0.4(0.59 – 0.2)	7.19(5.27 – 9.14)
Cambodia	Male	20.47(30.79 – 14.31)	53.63(82.39 – 35.76)	33.16	1.36(2.04 – 0.94)	1.4(2.16 – 0.92)	0.11(-0.01 – 0.24)
Cameroon	Male	8.23(11.06 – 6.01)	17.62(25.32 – 11.44)	9.38	0.42(0.56 – 0.3)	0.32(0.47 – 0.21)	-0.88(-1.02 – -0.74)
Canada	Male	480.31(553.63 – 408.77)	457.61(579.15 – 352.34)	-22.70	3.54(4.1 – 3)	1.32(1.67 – 1.02)	-3.11(-3.4 – -2.83)
Central African Republic	Male	3.14(4.81 – 1.95)	4.04(5.9 – 2.69)	0.90	0.74(1.08 – 0.47)	0.54(0.77 – 0.36)	-1.02(-1.15 – -0.89)

Chad	Male	5.03(7.08 – 3.03)	8.74(13.06 – 5.23)	3.71	0.42(0.59 – 0.25)	0.34(0.51 – 0.21)	-0.62(-0.76 – -0.48)
Chile	Male	43.79(53.61 – 34.42)	64.89(81.61 – 49.09)	21.09	1.02(1.26 – 0.78)	0.57(0.72 – 0.43)	-1.85(-2.51 – -1.18)
China	Male	7823.91(9909.23 – 5005.13)	16419.24(22664.98 – 12229.95)	8595.33	2.73(3.4 – 1.74)	1.94(2.64 – 1.46)	-1.15(-1.33 – -0.97)
Colombia	Male	46.9(57.31 – 38.45)	64.47(86.76 – 47.3)	17.56	0.63(0.77 – 0.51)	0.27(0.36 – 0.2)	-2.74(-3.59 – -1.89)
Comoros	Male	0.52(0.85 – 0.31)	1.03(1.85 – 0.5)	0.51	0.71(1.14 – 0.42)	0.59(1.06 – 0.29)	-0.61(-0.71 – -0.51)
Congo	Male	3.47(5.07 – 2.13)	8.18(11.99 – 5.56)	4.72	0.91(1.33 – 0.59)	0.88(1.26 – 0.6)	-0.06(-0.19 – 0.07)
Cook Islands	Male	0.05(0.07 – 0.04)	0.1(0.14 – 0.07)	0.05	0.92(1.22 – 0.66)	0.81(1.1 – 0.56)	-0.43(-0.64 – -0.22)
Costa Rica	Male	8.9(10.62 – 7.41)	17.3(21.81 – 13.12)	8.39	1.13(1.35 – 0.94)	0.71(0.9 – 0.54)	-1.35(-1.75 – -0.94)
Croatia	Male	101.19(122.2 – 84.17)	145.25(178.93 – 114.52)	44.05	5.17(6.23 – 4.28)	3.74(4.6 – 2.95)	-0.3(-0.45 – -0.14)
Cuba	Male	105.45(123.22 – 87.37)	185.89(231.13 – 143.82)	80.44	2.12(2.49 – 1.75)	2.01(2.5 – 1.56)	-1.12(-1.82 – -0.42)
Cyprus	Male	14.45(20.02 – 10.43)	25.62(35.89 – 18.05)	11.17	5.62(7.77 – 3.87)	2.8(3.97 – 1.95)	-0.09(-0.23 – 0.05)
Czechia	Male	224.03(268.43 – 185.81)	251.05(319.61 – 195.62)	27.02	4.03(4.84 – 3.35)	2.56(3.29 – 1.98)	-2.32(-2.95 – -1.7)
C么te d'Ivoire	Male	7.28(9.99 – 4.83)	18.77(27.66 – 12.27)	11.49	0.48(0.66 – 0.32)	0.44(0.63 – 0.29)	-1.42(-1.71 – -1.13)
Democratic People's Republic of Korea	Male	79(108.52 – 54.28)	146.46(221.8 – 104.64)	67.46	1.63(2.2 – 1.1)	1.24(1.88 – 0.88)	-0.89(-0.96 – -0.82)
Democratic Republic of the Congo	Male	24.28(35.04 – 15.5)	44.98(66.9 – 29.19)	20.71	0.39(0.57 – 0.25)	0.34(0.52 – 0.22)	-0.43(-0.56 – -0.31)

Denmark	Male	152.21(200.21 – 119.93)	146.43(176.45 – 121.31)	-5.78	4.4(5.75 – 3.48)	2.51(3.02 – 2.09)	-1.78(-3.02 – -0.53)
Djibouti	Male	0.44(0.7 – 0.27)	2.14(3.64 – 1.2)	1.70	1.02(1.57 – 0.65)	1(1.66 – 0.58)	-0.08(-0.17 – 0.01)
Dominica	Male	0.2(0.28 – 0.14)	0.25(0.37 – 0.17)	0.05	0.87(1.21 – 0.61)	0.7(1.04 – 0.47)	-0.68(-0.81 – -0.55)
Dominican Republic	Male	7.3(9.83 – 5.27)	20.92(31.64 – 13.37)	13.62	0.47(0.64 – 0.33)	0.46(0.7 – 0.3)	-0.05(-0.88 – 0.78)
Ecuador	Male	9.99(12.27 – 7.96)	23.9(32.16 – 16.69)	13.92	0.45(0.56 – 0.36)	0.34(0.46 – 0.24)	-0.61(-2.54 – 1.36)
Egypt	Male	627.57(797.93 – 387.57)	797.52(1420.21 – 527.05)	169.94	4.89(6.3 – 3.47)	2.8(5.13 – 1.81)	-1.72(-2.05 – -1.4)
El Salvador	Male	1.81(2.55 – 1.25)	4.25(6 – 2.87)	2.44	0.14(0.2 – 0.09)	0.16(0.23 – 0.11)	0.57(-0.36 – 1.51)
Equatorial Guinea	Male	0.45(0.68 – 0.28)	1.12(1.82 – 0.67)	0.67	0.61(0.9 – 0.39)	0.66(1.11 – 0.4)	0.3(0.06 – 0.53)
Eritrea	Male	1.71(2.64 – 1)	3.22(5.03 – 2.02)	1.50	0.45(0.68 – 0.27)	0.37(0.58 – 0.23)	-0.63(-0.73 – -0.52)
Estonia	Male	19.49(23 – 16.25)	22.54(27.52 – 17.73)	3.06	2.78(3.31 – 2.3)	2.21(2.7 – 1.74)	-0.81(-1.33 – -0.28)
Eswatini	Male	0.53(0.76 – 0.36)	0.78(1.14 – 0.48)	0.25	0.6(0.87 – 0.39)	0.46(0.67 – 0.29)	-0.79(-0.89 – -0.69)
Ethiopia	Male	24.54(40.2 – 12.75)	34.17(58.71 – 20.17)	9.63	0.27(0.43 – 0.14)	0.18(0.31 – 0.11)	-1.19(-1.39 – -0.99)
Fiji	Male	0.85(1.12 – 0.56)	1.64(2.39 – 0.87)	0.79	0.59(0.77 – 0.38)	0.54(0.77 – 0.3)	-0.21(-0.35 – -0.07)
Finland	Male	57.4(68.38 – 46.18)	47.02(60.06 – 35.76)	-10.39	2.16(2.6 – 1.74)	0.78(0.98 – 0.59)	-3.44(-3.78 – -3.1)
France	Male	1483.22(1730.22 – 1247.84)	1460.63(1823.82 – 1153.28)	-22.59	4.45(5.17 – 3.72)	2.22(2.76 – 1.77)	-2.15(-2.23 – -2.07)

Gabon	Male	1.66(2.35 – 1.07)	2.85(4.26 – 1.99)	1.19	0.73(1.02 – 0.48)	0.73(1.1 – 0.5)	0(-0.12 – 0.13)
Gambia	Male	0.58(0.81 – 0.37)	1.2(1.73 – 0.81)	0.62	0.4(0.54 – 0.25)	0.31(0.46 – 0.21)	-0.82(-1.36 – -0.28)
Georgia	Male	73.69(93.6 – 56.45)	117.01(140.66 – 94.94)	43.32	3.22(4.09 – 2.42)	5.01(5.99 – 4.06)	1.67(-0.2 – 3.57)
Germany	Male	1911.04(2204.21 – 1613.18)	1585.69(1954.27 – 1249.09)	-325.35	4.14(4.8 – 3.48)	1.71(2.09 – 1.35)	-2.87(-3.21 – -2.53)
Ghana	Male	8.74(12.12 – 5.99)	21.35(33.05 – 13.99)	12.61	0.44(0.61 – 0.3)	0.45(0.69 – 0.29)	0.15(-0.03 – 0.32)
Greece	Male	480.5(541.15 – 416)	542.57(645.58 – 445.74)	62.07	7.03(7.92 – 6.09)	4.48(5.28 – 3.74)	-1.46(-1.88 – -1.04)
Greenland	Male	0.43(0.57 – 0.25)	0.58(0.83 – 0.35)	0.15	3.45(4.52 – 1.99)	1.76(2.51 – 1.06)	-2.12(-2.43 – -1.8)
Grenada	Male	0.21(0.27 – 0.15)	0.29(0.38 – 0.22)	0.09	0.67(0.88 – 0.5)	0.66(0.87 – 0.49)	0.2(-0.77 – 1.18)
Guam	Male	0.18(0.23 – 0.13)	0.44(0.57 – 0.33)	0.27	0.47(0.64 – 0.34)	0.44(0.56 – 0.32)	-0.23(-0.85 – 0.41)
Guatemala	Male	3.04(3.84 – 2.36)	6.46(8.37 – 4.75)	3.41	0.24(0.31 – 0.18)	0.14(0.18 – 0.1)	-1.83(-3.2 – -0.44)
Guinea	Male	9.99(14.11 – 6.72)	18.51(27.17 – 12.03)	8.52	0.67(0.94 – 0.45)	0.75(1.1 – 0.49)	0.34(0.26 – 0.42)
Guinea-Bissau	Male	0.53(0.82 – 0.29)	0.98(1.49 – 0.64)	0.45	0.31(0.46 – 0.18)	0.35(0.54 – 0.23)	0.45(0.27 – 0.63)
Guyana	Male	0.79(1.01 – 0.62)	1.13(1.57 – 0.76)	0.33	0.48(0.61 – 0.38)	0.41(0.57 – 0.28)	-0.41(-0.93 – 0.11)
Haiti	Male	6.57(10.46 – 4.09)	9.7(15.13 – 5.66)	3.13	0.48(0.79 – 0.29)	0.34(0.52 – 0.2)	-1.1(-1.18 – -1.03)
Honduras	Male	1.74(2.44 – 1.18)	6.88(10.61 – 4.08)	5.14	0.2(0.29 – 0.14)	0.27(0.41 – 0.16)	0.87(0.77 – 0.98)

Hungary	Male	228.48(270.73 – 189.22)	235.56(297.78 – 184.23)	7.09	3.81(4.55 – 3.16)	2.89(3.64 – 2.27)	-0.74(-1.27 – -0.22)
Iceland	Male	3.73(4.42 – 3.05)	3.45(4.42 – 2.68)	-0.28	2.84(3.37 – 2.31)	1.19(1.53 – 0.93)	-2.72(-3.17 – -2.27)
India	Male	1093.14(1438.46 – 719)	2355.42(3060.52 – 1815.22)	1262.28	0.59(0.78 – 0.39)	0.49(0.64 – 0.38)	-0.55(-1.34 – 0.26)
Indonesia	Male	234.22(319.83 – 175.61)	648.28(1171.66 – 401.12)	414.06	0.63(0.88 – 0.47)	0.74(1.39 – 0.46)	0.53(0.46 – 0.61)
Iran (Islamic Republic of)	Male	111(147.78 – 78.32)	328.77(410.77 – 243.56)	217.77	0.97(1.3 – 0.67)	0.93(1.17 – 0.68)	-0.11(-0.35 – 0.13)
Iraq	Male	113.54(161.62 – 75.64)	326.28(479.78 – 207.42)	212.74	3.28(4.68 – 2.2)	3.78(5.5 – 2.44)	0.52(0.22 – 0.81)
Ireland	Male	55.4(64.21 – 46.97)	44.23(55.14 – 34.78)	-11.17	3.16(3.67 – 2.67)	1.15(1.44 – 0.9)	-3.21(-3.79 – -2.63)
Israel	Male	63.6(75.49 – 52.11)	98.01(123.2 – 76.32)	34.41	2.86(3.4 – 2.34)	1.69(2.12 – 1.32)	-1.64(-2.07 – -1.2)
Italy	Male	2156.72(2448.86 – 1842.45)	1725.24(2108.13 – 1382.21)	-431.48	5.75(6.56 – 4.9)	2.44(2.96 – 1.97)	-2.74(-3.05 – -2.43)
Jamaica	Male	7.13(8.8 – 5.68)	10.26(14.23 – 6.95)	3.13	0.85(1.04 – 0.68)	0.71(0.99 – 0.48)	-0.5(-2.57 – 1.62)
Japan	Male	1274.52(1424.12 – 1125.31)	2126.59(2615.61 – 1658.97)	852.07	2(2.25 – 1.75)	1.11(1.33 – 0.9)	-1.86(-2.08 – -1.64)
Jordan	Male	13.77(18.29 – 9.99)	57.75(82.08 – 39.59)	43.98	2.56(3.39 – 1.84)	1.82(2.55 – 1.28)	-1.08(-1.31 – -0.84)
Kazakhstan	Male	88.36(122.32 – 64.55)	86.37(107.67 – 67.32)	-1.99	2.03(2.77 – 1.47)	1.32(1.65 – 1.03)	-1.34(-1.86 – -0.82)
Kenya	Male	6.95(10.79 – 4.21)	18.25(24.24 – 12.82)	11.30	0.22(0.33 – 0.13)	0.24(0.32 – 0.16)	0.32(0.16 – 0.48)
Kiribati	Male	0.02(0.03 – 0.02)	0.05(0.07 – 0.03)	0.03	0.18(0.23 – 0.13)	0.19(0.26 – 0.13)	0.26(0.22 – 0.31)

Kuwait	Male	3.07(3.74 – 2.43)	17.39(22.55 – 13.17)	14.32	1.2(1.47 – 0.94)	1.32(1.71 – 0.99)	0.28(-2.02 – 2.64)
Kyrgyzstan	Male	17.12(20.9 – 13.27)	23.13(29.67 – 17.95)	6.02	1.7(2.1 – 1.33)	1.32(1.67 – 1.02)	-0.82(-1.87 – 0.25)
Lao People's Democratic Republic	Male	9.56(15.39 – 5.45)	16.74(26.06 – 10.55)	7.18	1.18(1.89 – 0.68)	0.99(1.55 – 0.61)	-0.59(-0.67 – -0.52)
Latvia	Male	41.1(47.92 – 35.06)	51.64(63.46 – 40.92)	10.54	3.38(3.97 – 2.85)	3.54(4.35 – 2.81)	0.19(-0.28 – 0.66)
Lebanon	Male	57.11(82.24 – 35.53)	164.37(230.93 – 115.85)	107.26	6.41(9.28 – 3.97)	5.59(7.84 – 3.94)	-0.42(-0.6 – -0.23)
Lesotho	Male	2.31(3.56 – 1.55)	4.54(7.13 – 2.52)	2.23	0.94(1.45 – 0.64)	1.32(2.04 – 0.75)	1.18(0.94 – 1.42)
Liberia	Male	1.7(2.49 – 1.12)	2.03(2.98 – 1.3)	0.33	0.31(0.45 – 0.2)	0.21(0.32 – 0.14)	-1.15(-1.32 – -0.98)
Libya	Male	28.92(42.01 – 18.61)	85.04(123.04 – 58.8)	56.12	3.41(4.94 – 2.15)	3.94(5.8 – 2.71)	0.45(0.08 – 0.82)
Lithuania	Male	60.19(70.22 – 49.96)	68.93(83.11 – 55.15)	8.74	3.68(4.33 – 3.06)	3.22(3.88 – 2.57)	-0.7(-3.05 – 1.72)
Luxembourg	Male	8.72(10.34 – 7.25)	8.4(10.46 – 6.44)	-0.33	4.07(4.83 – 3.33)	1.72(2.14 – 1.31)	-2.93(-3.61 – -2.24)
Madagascar	Male	9.51(13.27 – 6.62)	8.42(12.17 – 5.28)	-1.08	0.47(0.67 – 0.32)	0.24(0.36 – 0.15)	-2.16(-2.41 – -1.91)
Malawi	Male	28.07(37.12 – 20.5)	65.21(91.11 – 44.18)	37.14	2.24(2.98 – 1.63)	2.89(4.09 – 1.96)	0.87(0.71 – 1.03)
Malaysia	Male	64.6(93.98 – 35.59)	169.12(232.36 – 109.64)	104.53	1.7(2.51 – 0.92)	1.4(1.93 – 0.89)	-0.98(-1.38 – -0.59)
Maldives	Male	0.43(0.65 – 0.28)	1.16(1.53 – 0.86)	0.73	1.16(1.72 – 0.74)	0.85(1.12 – 0.63)	-1.02(-1.26 – -0.78)
Mali	Male	21.17(28.06 – 15.02)	64.8(92.77 – 44.4)	43.63	1.48(2 – 1.05)	1.96(2.83 – 1.32)	0.96(0.79 – 1.13)

Malta	Male	7.01(8.36 – 5.74)	6.75(8.68 – 5.21)	-0.25	3.8(4.55 – 3.07)	1.45(1.87 – 1.13)	-2.97(-3.59 – -2.34)
Marshall Islands	Male	0.04(0.07 – 0.03)	0.1(0.15 – 0.06)	0.06	0.62(0.94 – 0.4)	0.67(0.98 – 0.42)	0.28(0.18 – 0.39)
Mauritania	Male	1.98(2.91 – 1.38)	2.92(4.83 – 1.64)	0.94	0.48(0.7 – 0.33)	0.3(0.51 – 0.17)	-1.51(-1.63 – -1.39)
Mauritius	Male	7.38(8.49 – 6.33)	9.77(11.39 – 8.29)	2.40	2.96(3.42 – 2.47)	1.31(1.54 – 1.09)	-2.6(-3.42 – -1.77)
Mexico	Male	131.34(154.04 – 110.37)	201.87(250.58 – 156.65)	70.54	0.75(0.88 – 0.63)	0.38(0.47 – 0.29)	-2.21(-2.87 – -1.54)
Micronesia (Federated States of)	Male	0.18(0.26 – 0.11)	0.28(0.4 – 0.17)	0.10	0.83(1.18 – 0.53)	0.86(1.22 – 0.55)	0.11(0.09 – 0.13)
Monaco	Male	1.05(1.63 – 0.61)	1.23(3.52 – 0.53)	0.18	3.41(5.31 – 2.01)	2.52(7.23 – 1.09)	-0.98(-1.06 – -0.89)
Mongolia	Male	3.74(5.57 – 2.26)	6(8.76 – 4.14)	2.26	0.86(1.27 – 0.52)	0.7(1.01 – 0.48)	-0.69(-1.09 – -0.3)
Montenegro	Male	7.15(9.74 – 5.13)	13.11(18.23 – 9.11)	5.96	2.78(3.81 – 2.01)	3.11(4.32 – 2.15)	0.44(0.1 – 0.78)
Morocco	Male	40.43(54.4 – 26.4)	80.34(114.39 – 54.84)	39.91	0.62(0.83 – 0.39)	0.51(0.73 – 0.35)	-0.62(-0.7 – -0.54)
Mozambique	Male	11.44(17.06 – 7.34)	21.29(31.05 – 15.14)	9.84	0.55(0.8 – 0.36)	0.63(0.91 – 0.44)	0.43(0.32 – 0.54)
Myanmar	Male	90.34(130.95 – 63.06)	106.29(179.51 – 71.19)	15.95	1.05(1.52 – 0.74)	0.65(1.13 – 0.44)	-1.53(-1.59 – -1.48)
Namibia	Male	0.88(1.23 – 0.61)	1.83(2.39 – 1.4)	0.95	0.47(0.65 – 0.32)	0.47(0.62 – 0.36)	0.09(-0.04 – 0.21)
Nauru	Male	0.02(0.03 – 0.01)	0.02(0.03 – 0.01)	＜0.01	0.88(1.29 – 0.53)	0.95(1.4 – 0.54)	0.25(0.21 – 0.29)
Nepal	Male	22.51(36.6 – 12.69)	46.18(97.07 – 28.54)	23.67	0.64(1.05 – 0.35)	0.52(1.08 – 0.32)	-0.63(-0.81 – -0.45)

Netherlands	Male	416.71(474.04 – 353.75)	360.92(449.83 – 290.59)	-55.79	5.25(6 – 4.44)	2.14(2.68 – 1.73)	-2.84(-3.57 – -2.09)
New Zealand	Male	33.95(40.93 – 27.77)	46.14(59.19 – 34.51)	12.18	2.05(2.48 – 1.65)	1.13(1.45 – 0.85)	-1.85(-2.6 – -1.09)
Nicaragua	Male	1.24(1.63 – 0.88)	3.52(4.81 – 2.53)	2.29	0.22(0.29 – 0.16)	0.18(0.26 – 0.13)	-0.64(-0.97 – -0.3)
Niger	Male	1.95(3.12 – 1.23)	4.26(7.57 – 2.46)	2.30	0.23(0.37 – 0.14)	0.17(0.3 – 0.09)	-0.91(-1.04 – -0.78)
Nigeria	Male	10.07(16.33 – 6.22)	14.47(24.99 – 9.11)	4.40	0.06(0.1 – 0.04)	0.04(0.07 – 0.03)	-1.14(-1.23 – -1.04)
Niue	Male	0.01(0.01 – 0)	0.01(0.01 – 0)	＜0.01	0.65(0.88 – 0.44)	0.7(0.98 – 0.46)	0.23(0.18 – 0.29)
North Macedonia	Male	28.94(36.7 – 22.46)	50.42(67.36 – 34.96)	21.48	3.51(4.48 – 2.7)	3.47(4.62 – 2.44)	-0.11(-0.52 – 0.31)
Northern Mariana Islands	Male	0.05(0.08 – 0.03)	0.19(0.24 – 0.14)	0.14	0.61(1.02 – 0.42)	0.8(1.05 – 0.58)	0.85(0.7 – 1)
Norway	Male	108.88(127.21 – 93.43)	58.59(73.29 – 44.54)	-50.29	3.61(4.22 – 3.09)	1.2(1.5 – 0.9)	-3.51(-3.74 – -3.28)
Oman	Male	1.66(2.47 – 1.03)	3(4.11 – 2.08)	1.34	0.61(0.91 – 0.37)	0.45(0.63 – 0.3)	-1.05(-2.11 – 0.03)
Pakistan	Male	724.61(964.49 – 525.8)	1169.47(1735.19 – 820.47)	444.87	2.59(3.44 – 1.87)	2.3(3.41 – 1.57)	-0.4(-0.45 – -0.34)
Palau	Male	0.01(0.02 – 0.01)	0.03(0.03 – 0.02)	0.01	0.31(0.43 – 0.22)	0.23(0.31 – 0.16)	-1.04(-1.19 – -0.88)
Palestine	Male	10.8(15.07 – 7.18)	22.73(30.69 – 16.92)	11.93	3.29(4.59 – 2.21)	2.59(3.59 – 1.91)	-0.8(-1.17 – -0.43)
Panama	Male	2.45(2.95 – 2.02)	5.34(7.2 – 3.8)	2.89	0.36(0.43 – 0.3)	0.26(0.35 – 0.18)	-0.99(-1.4 – -0.58)
Papua New Guinea	Male	2.36(3.9 – 1.14)	6.26(10.44 – 3.05)	3.89	0.29(0.47 – 0.14)	0.25(0.41 – 0.13)	-0.43(-0.6 – -0.27)

Paraguay	Male	8.22(10.76 – 6.05)	20.69(29.05 – 13.89)	12.48	0.91(1.19 – 0.67)	0.88(1.25 – 0.59)	-0.08(-0.63 – 0.47)
Peru	Male	15.43(21.76 – 10.45)	36.64(55.89 – 22.79)	21.21	0.3(0.43 – 0.2)	0.24(0.37 – 0.15)	-0.67(-1.6 – 0.28)
Philippines	Male	66.23(86.74 – 50.31)	170.4(250.22 – 125.58)	104.17	0.54(0.74 – 0.41)	0.52(0.79 – 0.38)	-0.12(-0.34 – 0.1)
Poland	Male	807.46(903.02 – 709.2)	1200.17(1449.01 – 973.45)	392.71	4.61(5.2 – 4.03)	3.96(4.8 – 3.18)	-0.58(-0.74 – -0.42)
Portugal	Male	155.54(184.13 – 129.17)	180.72(224.65 – 139.62)	25.18	2.82(3.39 – 2.33)	1.63(2 – 1.29)	-1.74(-2.39 – -1.09)
Puerto Rico	Male	15.06(19.22 – 11.46)	22.75(30.06 – 16.4)	7.69	0.93(1.19 – 0.7)	0.67(0.89 – 0.49)	-1.05(-2.22 – 0.13)
Qatar	Male	0.84(1.18 – 0.57)	3.39(5.35 – 2.03)	2.55	1.85(2.6 – 1.29)	0.95(1.48 – 0.6)	-2.15(-3.1 – -1.2)
Republic of Korea	Male	264.57(327.51 – 196.16)	576.98(764.4 – 387.81)	312.41	3.31(4.18 – 2.45)	1.65(2.21 – 1.07)	-2.26(-2.44 – -2.07)
Republic of Moldova	Male	40.98(47.89 – 33.49)	60.43(70.92 – 49.81)	19.45	2.41(2.84 – 1.94)	2.45(2.89 – 2.01)	-0.04(-1.48 – 1.41)
Romania	Male	306.22(356.03 – 257.48)	466.95(571.67 – 370.57)	160.73	2.48(2.9 – 2.08)	2.9(3.55 – 2.32)	0.5(0.11 – 0.9)
Russian Federation	Male	1935.69(2173.14 – 1707.78)	2133.72(2523.3 – 1762.13)	198.03	3.45(3.91 – 3.03)	2.44(2.89 – 2.01)	-1.08(-1.53 – -0.62)
Rwanda	Male	10.8(15.73 – 7.64)	17.63(29.41 – 9.93)	6.82	1.12(1.62 – 0.79)	0.98(1.6 – 0.57)	-0.42(-0.51 – -0.34)
Saint Kitts and Nevis	Male	0.12(0.15 – 0.09)	0.14(0.19 – 0.1)	0.02	0.73(0.97 – 0.53)	0.56(0.79 – 0.39)	-0.71(-0.99 – -0.43)
Saint Lucia	Male	0.43(0.53 – 0.34)	0.79(1.05 – 0.57)	0.36	1.3(1.66 – 1.02)	0.75(1.01 – 0.53)	-1.77(-2.39 – -1.14)
Saint Vincent and the Grenadines	Male	0.2(0.26 – 0.16)	0.42(0.54 – 0.32)	0.22	0.69(0.88 – 0.53)	0.61(0.79 – 0.45)	-0.45(-1.05 – 0.15)

Samoa	Male	0.35(0.5 – 0.25)	0.5(0.7 – 0.34)	0.15	1(1.39 – 0.73)	0.8(1.11 – 0.54)	-0.7(-0.76 – -0.64)
San Marino	Male	0.79(1.06 – 0.55)	0.67(1 – 0.41)	-0.12	4.94(6.69 – 3.47)	1.7(2.54 – 1.03)	-3.83(-4.28 – -3.38)
Sao Tome and Principe	Male	0.11(0.16 – 0.08)	0.26(0.41 – 0.16)	0.15	0.47(0.67 – 0.33)	0.59(0.92 – 0.36)	0.71(0.2 – 1.22)
Saudi Arabia	Male	14.45(22.44 – 8.86)	43.2(80.63 – 26.42)	28.75	0.5(0.78 – 0.31)	0.45(0.8 – 0.28)	-0.41(-0.49 – -0.34)
Senegal	Male	7.45(10.1 – 5.12)	11.39(17.23 – 7.38)	3.94	0.47(0.64 – 0.33)	0.33(0.5 – 0.21)	-1.23(-1.46 – -1.01)
Serbia	Male	150.35(200.54 – 109.33)	216.16(303.75 – 147.82)	65.81	3.5(4.71 – 2.53)	2.84(3.97 – 1.95)	-0.68(-0.99 – -0.38)
Seychelles	Male	0.8(1.03 – 0.61)	1.05(1.38 – 0.8)	0.24	3.62(4.6 – 2.73)	2.4(3.18 – 1.85)	-1.3(-2.65 – 0.07)
Sierra Leone	Male	4.77(6.43 – 3.32)	5.02(7.34 – 3.54)	0.25	0.5(0.69 – 0.35)	0.31(0.44 – 0.22)	-1.55(-1.67 – -1.44)
Singapore	Male	9.22(10.98 – 7.54)	14.98(18.52 – 11.43)	5.76	1.04(1.24 – 0.84)	0.39(0.49 – 0.29)	-3.2(-4.17 – -2.22)
Slovakia	Male	88.59(115.6 – 68.28)	99.68(138.34 – 70.3)	11.10	3.63(4.75 – 2.78)	2.55(3.49 – 1.81)	-1.11(-1.32 – -0.91)
Slovenia	Male	22.13(26.81 – 18.18)	33.93(43.83 – 25.7)	11.80	2.3(2.81 – 1.88)	1.69(2.19 – 1.28)	-1.02(-1.26 – -0.77)
Solomon Islands	Male	0.46(0.73 – 0.22)	1.19(1.89 – 0.63)	0.73	0.67(1.06 – 0.33)	0.74(1.18 – 0.41)	0.33(0.22 – 0.44)
Somalia	Male	4.23(7.48 – 2.35)	7.88(13.72 – 3.9)	3.65	0.52(0.92 – 0.28)	0.42(0.74 – 0.21)	-0.62(-0.68 – -0.55)
South Africa	Male	84.86(122.64 – 59.41)	126.61(156.74 – 97.31)	41.75	1.16(1.68 – 0.81)	0.77(0.97 – 0.58)	-1.32(-1.9 – -0.74)
South Sudan	Male	6.36(9.72 – 4.03)	6.45(10.29 – 3.99)	0.09	0.5(0.73 – 0.31)	0.41(0.65 – 0.26)	-0.61(-0.71 – -0.51)

Spain	Male	1483.3(1703.32 – 1264.29)	1390.43(1724.14 – 1108.39)	-92.87	6.43(7.37 – 5.52)	3.02(3.73 – 2.41)	-2.41(-2.64 – -2.17)
Sri Lanka	Male	25.55(34.39 – 18.79)	40.71(59.38 – 22.61)	15.16	0.6(0.8 – 0.44)	0.4(0.58 – 0.23)	-1.16(-1.93 – -0.39)
Sudan	Male	54.91(98.17 – 29.71)	88.49(130.32 – 59.27)	33.58	1.27(2.27 – 0.68)	0.97(1.43 – 0.66)	-0.87(-0.91 – -0.84)
Suriname	Male	0.87(1.16 – 0.62)	1.75(2.53 – 1.12)	0.89	0.77(1.03 – 0.54)	0.63(0.92 – 0.4)	-0.49(-1.35 – 0.37)
Sweden	Male	146.19(174.36 – 119.81)	139.06(175.51 – 104.21)	-7.13	2.13(2.55 – 1.75)	1.23(1.55 – 0.93)	-1.76(-2.18 – -1.33)
Switzerland	Male	83.79(99.27 – 69.38)	146.44(179.9 – 116.43)	62.64	1.95(2.31 – 1.62)	1.62(1.99 – 1.29)	-0.67(-1.69 – 0.36)
Syrian Arab Republic	Male	37.81(52.27 – 26.81)	81.41(117.08 – 53.6)	43.60	1.56(2.17 – 1.1)	1.36(1.98 – 0.89)	-0.53(-0.96 – -0.1)
Taiwan (Province of China)	Male	173(198.99 – 148.19)	375.49(442.89 – 308.16)	202.49	2.53(2.9 – 2.13)	1.92(2.26 – 1.58)	-0.72(-1.03 – -0.4)
Tajikistan	Male	7.87(12.97 – 5.06)	8.03(12.31 – 5.1)	0.16	0.74(1.29 – 0.46)	0.33(0.49 – 0.21)	-2.63(-3.12 – -2.13)
Thailand	Male	280.92(363.73 – 194.74)	614.38(859.15 – 439.26)	333.46	2.3(3.01 – 1.56)	1.29(1.8 – 0.93)	-1.84(-2.3 – -1.38)
Timor-Leste	Male	0.54(0.89 – 0.31)	1.86(3.01 – 1.19)	1.32	0.55(0.87 – 0.31)	0.52(0.85 – 0.33)	-0.17(-0.34 – 0)
Togo	Male	4.21(5.69 – 2.99)	8.43(12.19 – 5.69)	4.22	0.91(1.25 – 0.64)	0.68(0.98 – 0.45)	-0.96(-1.07 – -0.86)
Tokelau	Male	0(0.01 – 0)	0(0.01 – 0)	＜0.01	0.68(1.01 – 0.43)	0.58(0.82 – 0.36)	-0.56(-0.59 – -0.52)
Tonga	Male	0.2(0.34 – 0.12)	0.32(0.51 – 0.19)	0.11	0.91(1.51 – 0.54)	0.93(1.52 – 0.57)	0.05(-0.25 – 0.35)
Trinidad and Tobago	Male	2.97(3.52 – 2.41)	5.14(6.87 – 3.51)	2.17	0.82(0.99 – 0.66)	0.56(0.76 – 0.39)	-0.97(-1.39 – -0.55)

Tunisia	Male	63.52(88.93 – 44.07)	162.86(231.76 – 100.8)	99.34	3.04(4.12 – 2.1)	2.91(4.11 – 1.83)	-0.18(-0.32 – -0.03)
Turkey	Male	597.53(815.46 – 418.71)	1143.13(1561.44 – 825.38)	545.60	4.2(5.74 – 3)	2.82(3.85 – 2.05)	-1.26(-1.68 – -0.85)
Turkmenistan	Male	7.29(8.71 – 6.1)	10.18(13.9 – 7.26)	2.89	1.02(1.24 – 0.84)	0.62(0.84 – 0.45)	-1.74(-2.56 – -0.91)
Tuvalu	Male	0.02(0.03 – 0.01)	0.03(0.05 – 0.02)	0.01	0.75(1.09 – 0.48)	0.72(1.05 – 0.46)	-0.15(-0.18 – -0.11)
Uganda	Male	10.2(14.29 – 7)	17.95(26.04 – 12.62)	7.75	0.41(0.56 – 0.28)	0.38(0.53 – 0.26)	-0.29(-0.44 – -0.14)
Ukraine	Male	755.07(1006.02 – 586.81)	686.46(962.53 – 447.1)	-68.61	3.2(4.2 – 2.52)	2.34(3.29 – 1.54)	-0.97(-1.43 – -0.51)
United Arab Emirates	Male	3.93(7.7 – 2.08)	16.99(23.93 – 11.77)	13.07	1.95(3.92 – 1.04)	0.99(1.39 – 0.69)	-2.4(-3.9 – -0.88)
United Kingdom	Male	1865.58(2122.61 – 1626.81)	1151.24(1431.56 – 899.92)	-714.34	5.11(5.83 – 4.41)	1.77(2.2 – 1.4)	-3.35(-3.51 – -3.18)
United Republic of Tanzania	Male	31.59(46.67 – 21.95)	45.2(67.02 – 29.32)	13.61	0.73(1.06 – 0.52)	0.46(0.69 – 0.3)	-1.51(-1.64 – -1.38)
United States of America	Male	2674.92(3119.25 – 2263.23)	3795.56(4701 – 3024.82)	1120.64	2.02(2.37 – 1.7)	1.4(1.73 – 1.11)	-1.19(-1.43 – -0.95)
United States Virgin Islands	Male	0.18(0.26 – 0.12)	0.27(0.42 – 0.16)	0.09	0.53(0.75 – 0.36)	0.32(0.49 – 0.19)	-1.58(-2.2 – -0.95)
Uruguay	Male	60.4(71.36 – 50.12)	65.33(80.18 – 52.28)	4.93	3.53(4.19 – 2.92)	2.76(3.38 – 2.22)	-0.85(-1.51 – -0.19)
Uzbekistan	Male	17.51(24.8 – 11.63)	58.76(78.56 – 42.49)	41.25	0.39(0.58 – 0.25)	0.56(0.75 – 0.41)	0.99(0.43 – 1.55)
Vanuatu	Male	0.15(0.23 – 0.09)	0.34(0.53 – 0.2)	0.19	0.51(0.77 – 0.29)	0.45(0.68 – 0.27)	-0.41(-0.59 – -0.23)

Venezuela (Bolivarian Republic of)	Male	28.93(34.04 – 23.63)	62.44(86.29 – 43.74)	33.51	0.73(0.87 – 0.59)	0.51(0.7 – 0.36)	-1.21(-2.38 – -0.02)
Viet Nam	Male	132.82(177.11 – 89.25)	345.56(439.97 – 260.97)	212.75	0.96(1.27 – 0.67)	1.02(1.3 – 0.78)	0.22(0.13 – 0.3)
Yemen	Male	32.92(53.53 – 17.72)	94.18(132.78 – 62.09)	61.26	1.69(2.68 – 0.91)	1.61(2.29 – 1.07)	-0.17(-0.23 – -0.1)
Zambia	Male	5.47(7.55 – 4.02)	13.24(26.67 – 7.09)	7.77	0.43(0.61 – 0.32)	0.5(0.94 – 0.29)	0.53(0.36 – 0.69)
Zimbabwe	Male	40.74(55.26 – 29.16)	70.41(95.17 – 52.11)	29.66	2.64(3.52 – 1.89)	3.04(4.07 – 2.26)	0.5(0.36 – 0.65)

Supplementary Table 4. The DALYs and age-standardized DALY rate of bladder cancer attributable to smoking in 1990 and 2021, and its temporal trends from 1990 to 2021.

Nation	Sex	DALY No. (95% UI)		Change in absolute number (%)	Age-standardized DALY rate per 100,000 No. (95% UI)		1990-2021 AAPC No.(95 CI)
		1990	2021		1990	2021	
Afghanistan	Both	660.37(1211.35 – 348.38)	994.71(1528.13 – 544.48)	334.34	9.45(17.23 – 5.04)	10.48(15.88 – 5.9)	0.36(0.29 – 0.43)
Albania	Both	50.97(67.86 – 38.51)	105.31(150.4 – 71.8)	54.34	2.63(3.53 – 1.99)	2.33(3.33 – 1.59)	-0.45(-0.95 – 0.06)
Algeria	Both	1301.29(1714.51 – 883.79)	2895.83(3958.61 – 2047.12)	1594.54	12.7(16.63 – 8.62)	9.38(12.92 – 6.6)	-0.98(-1.12 – -0.83)
American Samoa	Both	1.86(2.49 – 1.38)	4.6(6 – 3.5)	2.74	7.93(10.77 – 5.76)	9.07(11.79 – 6.89)	0.5(0.18 – 0.82)
Andorra	Both	28.19(41.6 – 18.31)	37.24(54.86 – 23.17)	9.05	48.64(71.37 – 31.76)	23.98(35.41 – 14.91)	-2.39(-2.91 – -1.86)
Angola	Both	360.19(533.9 – 241.81)	946.58(1333.85 – 675.12)	586.39	9.57(14.07 – 6.53)	8.19(11.45 – 5.84)	-0.48(-0.72 – -0.24)
Antigua and Barbuda	Both	4.26(5.39 – 3.33)	7.65(9.6 – 5.9)	3.39	7.96(10.07 – 6.23)	7.15(9 – 5.48)	-0.33(-1.48 – 0.83)
Argentina	Both	9834.98(11555.73 – 8248.95)	8426.18(10155.24 – 6806.87)	-1408.8	29.67(34.83 – 24.8)	15.18(18.15 – 12.3)	-2.06(-2.46 – -1.65)
Armenia	Both	1442.04(1746.98 – 1173.15)	2238.47(2710.39 – 1807.37)	796.43	51.13(61.58 – 41.54)	50.89(61.61 – 41.12)	0.03(-0.72 – 0.77)
Australia	Both	4402.48(5164.02 – 3709.98)	3467.93(4445.5 – 2749.63)	-934.55	22.07(25.86 – 18.6)	7.75(9.76 – 6.22)	-3.36(-3.47 – -3.24)
Austria	Both	3450.32(4032.04 – 2935.61)	3232.27(3888.2 – 2648.31)	-218.05	29.51(34.39 – 25.22)	18.23(21.75 – 15.07)	-1.5(-2.15 – -0.85)

Azerbaijan	Both	965.49(1349.93 – 700.64)	1711.93(2454.34 – 1140.27)	746.44	18.25(25.73 – 13.41)	16.06(22.64 – 10.83)	-0.43(-0.84 – -0.02)
Bahamas	Both	8.67(11.1 – 6.46)	21.92(28.2 – 15.49)	13.25	5.67(7.31 – 4.23)	5.37(6.9 – 3.81)	-0.04(-0.43 – 0.35)
Bahrain	Both	79.22(103.98 – 58.55)	215.9(326.24 – 139.29)	136.68	51.02(66.68 – 37.27)	27.39(39.44 – 18.03)	-1.95(-2.43 – -1.47)
Bangladesh	Both	4610.74(7303.73 – 2801.89)	8478.79(17456.72 – 4757.31)	3868.05	10.51(16.7 – 6.33)	6.49(13.34 – 3.67)	-1.42(-1.72 – -1.12)
Barbados	Both	22.12(27.15 – 17.43)	29.96(41.1 – 20.52)	7.84	7.22(8.78 – 5.74)	5.64(7.71 – 3.89)	-0.62(-1.19 – -0.05)
Belarus	Both	4121.26(4703.76 – 3566.37)	3807.5(4662.01 – 2967.46)	-313.76	31.11(35.38 – 26.85)	23.17(28.4 – 17.95)	-0.88(-1.54 – -0.22)
Belgium	Both	7723.08(8885.51 – 6555.58)	5178.54(6199.84 – 4339.5)	-2544.54	49.81(57.28 – 42.53)	22.52(26.78 – 19.05)	-2.64(-3.65 – -1.62)
Belize	Both	5.28(6.41 – 4.28)	17.28(21.75 – 13.22)	12	5.78(7.02 – 4.68)	5.98(7.52 – 4.55)	0.27(-0.38 – 0.94)
Benin	Both	89.72(131.97 – 63.8)	113.65(158.29 – 78.15)	23.93	4.59(6.74 – 3.25)	2.25(3.18 – 1.55)	-2.26(-2.45 – -2.08)
Bermuda	Both	15.73(20.17 – 11.96)	22.62(29.68 – 16.81)	6.89	25.18(32.36 – 19.16)	16.34(21.35 – 12.16)	-1.46(-2.09 – -0.83)
Bhutan	Both	8.84(15.51 – 4.48)	19.26(44.19 – 10.93)	10.42	4.16(7.31 – 2.13)	3.37(7.72 – 1.91)	-0.63(-0.7 – -0.56)
Bolivia (Plurinational State of)	Both	215.94(308.93 – 145.35)	446.15(654.14 – 296.7)	230.21	7.04(10.01 – 4.73)	5.05(7.45 – 3.33)	-1.07(-1.14 – -1)
Bosnia and Herzegovina	Both	1080.61(1343.11 – 872.87)	2157.15(2864.34 – 1551.78)	1076.54	25.9(31.93 – 21.07)	33.61(44.64 – 24.1)	0.94(0.63 – 1.26)
Botswana	Both	63.66(86.69 – 43.96)	124.31(179.53 – 87.25)	60.65	11.76(15.9 – 8.25)	8.85(12.43 – 6.28)	-0.89(-1.11 – -0.68)
Brazil	Both	17495.97(19843.6 – 15307.67)	26059.46(31666.85 – 21409.72)	8563.49	20.15(22.97 – 17.53)	10.37(12.63 – 8.51)	-2.07(-2.45 – -1.69)

Brunei Darussalam	Both	20.74(27.79 – 15.69)	34.73(45.39 – 25.37)	13.99	21.99(29.34 – 16.64)	10.59(13.63 – 7.78)	-2.38(-2.66 – -2.1)
Bulgaria	Both	4150.11(4912.75 – 3472.92)	5023.68(6341.71 – 3972.69)	873.57	31.95(37.67 – 26.86)	36.7(46.68 – 28.81)	0.5(0.01 – 0.98)
Burkina Faso	Both	109.56(157.89 – 72.95)	190.79(268.65 – 133.76)	81.23	2.48(3.55 – 1.67)	1.97(2.79 – 1.39)	-0.75(-0.85 – -0.65)
Burundi	Both	186.17(288.29 – 117.24)	169.28(256.07 – 107.32)	-16.89	8.23(12.7 – 5.15)	3.61(5.36 – 2.36)	-2.63(-2.73 – -2.53)
Cabo Verde	Both	1.29(3.73 – 0.72)	16.45(24.27 – 8.07)	15.16	0.59(1.69 – 0.33)	3.65(5.31 – 1.81)	6.08(3.93 – 8.28)
Cambodia	Both	530.19(783.21 – 380.37)	1314.51(1966.56 – 897.06)	784.32	12.38(18.37 – 8.94)	11.06(16.76 – 7.54)	-0.34(-0.44 – -0.24)
Cameroon	Both	229.82(312.37 – 168.97)	510.23(722.08 – 335.64)	280.41	5.03(6.71 – 3.7)	3.79(5.41 – 2.5)	-0.92(-1.04 – -0.79)
Canada	Both	12957.63(14922.6 – 11092.26)	10915.01(13458.06 – 8735.58)	-2042.62	39.66(45.6 – 33.91)	14.78(18.12 – 11.93)	-3.05(-3.98 – -2.11)
Central African Republic	Both	89.13(135.22 – 54.5)	120.09(174.89 – 77.83)	30.96	7.73(11.52 – 4.94)	5.28(7.63 – 3.6)	-1.24(-1.36 – -1.11)
Chad	Both	118.81(166.6 – 73.91)	221.98(327.26 – 134.65)	103.17	4.31(6.01 – 2.68)	3.99(5.9 – 2.44)	-0.23(-0.33 – -0.12)
Chile	Both	1413.15(1702.27 – 1148.45)	1921.7(2336.62 – 1560.7)	508.55	13.82(16.68 – 11.17)	7.48(9.08 – 6.09)	-1.96(-2.55 – -1.38)
China	Both	202646.25(252972.51 – 131999.37)	359985.14(490838.27 – 266680.95)	157338.89	25.29(31.73 – 16.79)	17.09(23.35 – 12.74)	-1.26(-1.54 – -0.97)
Colombia	Both	1325.8(1570.96 – 1096.7)	1608.42(2139.06 – 1216.01)	282.62	7.69(9.18 – 6.4)	2.92(3.88 – 2.21)	-3.12(-3.88 – -2.36)
Comoros	Both	13(20.89 – 7.91)	23.51(40.45 – 12.2)	10.51	7.16(11.37 – 4.38)	5.27(9.15 – 2.75)	-1.02(-1.16 – -0.88)
Congo	Both	88.22(129.55 – 52.62)	206.35(298.55 – 139.21)	118.13	8.48(12.42 – 5.22)	8.32(12.14 – 5.68)	-0.02(-0.2 – 0.17)
Cook Islands	Both	1.86(2.47 – 1.36)	3.03(4.18 – 2.21)	1.17	14.52(19.04 – 10.59)	11.63(16.1 – 8.47)	-0.63(-0.72 – -0.54)

Costa Rica	Both	206.29(242.16 – 173.71)	391.3(487.06 – 306.17)	185.01	12.19(14.36 – 10.25)	7.14(8.9 – 5.58)	-1.67(-2.05 – -1.29)
Croatia	Both	2574.3(3028.3 – 2177.91)	3468.85(4280.2 – 2764.64)	894.55	42.59(50.17 – 35.69)	38.24(47.09 – 30.53)	-0.47(-0.61 – -0.32)
Cuba	Both	2547.09(2944.3 – 2173.08)	4428.2(5376.29 – 3489.28)	1881.11	24.88(28.87 – 21.31)	22.44(27.18 – 17.79)	-0.24(-0.62 – 0.14)
Cyprus	Both	318.48(428.8 – 242.13)	560.93(771.15 – 404.85)	242.45	42.84(57.33 – 32.05)	26.45(36.25 – 19.15)	-0.38(-0.75 – 0)
Czechia	Both	6080.05(7195.13 – 5093.89)	6504.1(8275.36 – 5092.18)	424.05	43.29(51.31 – 36.38)	29.89(37.75 – 23.32)	-1.64(-1.92 – -1.35)
C么te d'Ivoire	Both	203.44(281.74 – 136.33)	504.36(765.83 – 321.8)	300.92	5.4(7.34 – 3.62)	4.7(6.82 – 3.1)	-1.19(-1.47 – -0.9)
Democratic People's Republic of Korea	Both	2257.85(3098.29 – 1561.2)	4036.59(6009.03 – 2833.75)	1778.74	13.6(18.66 – 9.36)	11.92(17.65 – 8.35)	-0.43(-0.48 – -0.37)
Democratic Republic of the Congo	Both	670.99(949.84 – 447.56)	1288.71(1878.69 – 854.61)	617.72	4.32(6.17 – 2.86)	3.43(5.02 – 2.25)	-0.75(-0.88 – -0.62)
Denmark	Both	4196.69(5295.99 – 3410.69)	3630.7(4314.44 – 3055.84)	-565.99	52.15(65.02 – 42.65)	29.57(34.93 – 24.82)	-1.78(-2.55 – -1)
Djibouti	Both	12.39(19.75 – 7.46)	55.97(95.36 – 31.82)	43.58	10.32(15.94 – 6.48)	10.13(16.72 – 5.92)	-0.07(-0.21 – 0.07)
Dominica	Both	5.29(7.18 – 3.81)	6.61(9.44 – 4.58)	1.32	8.73(11.77 – 6.39)	7.73(11.03 – 5.36)	-0.39(-0.51 – -0.28)
Dominican Republic	Both	213.25(272.77 – 159.63)	567.63(822.25 – 385.52)	354.38	6.24(7.98 – 4.7)	5.79(8.35 – 3.95)	-0.24(-0.89 – 0.42)
Ecuador	Both	225.86(268.39 – 182.15)	520.48(695.12 – 365.29)	294.62	4.53(5.43 – 3.65)	3.23(4.33 – 2.28)	-0.87(-2.59 – 0.89)
Egypt	Both	18472.52(23646.22 – 9849.36)	20752.85(36280.51 – 13988.16)	2280.33	63.69(80.87 – 39.16)	34.21(61.01 – 22.78)	-2.04(-2.52 – -1.56)
El Salvador	Both	52.51(70.59 – 38.63)	122.99(170.69 – 88)	70.48	1.78(2.4 – 1.3)	2.03(2.81 – 1.45)	0.47(-0.38 – 1.32)

Equatorial Guinea	Both	12.3(18.58 – 7.37)	29.56(47 – 17.8)	17.26	6.27(9.38 – 4.01)	6.02(9.76 – 3.64)	-0.12(-0.35 – 0.1)
Eritrea	Both	54.55(83.91 – 31.91)	95.51(150.67 – 58.15)	40.96	4.35(6.65 – 2.57)	3.25(5.03 – 2.06)	-0.96(-1.1 – -0.83)
Estonia	Both	552.39(648.67 – 467.78)	542.63(658.45 – 437.81)	-9.76	26.65(31.29 – 22.57)	21.02(25.4 – 17.04)	-0.75(-1.28 – -0.21)
Eswatini	Both	17.01(23.46 – 11.9)	26.41(39.4 – 16.53)	9.4	6.38(8.81 – 4.48)	4.84(7.18 – 3.1)	-0.86(-1.06 – -0.66)
Ethiopia	Both	655.87(1048.04 – 365.14)	805.43(1374.93 – 477.06)	149.56	3.27(5.23 – 1.81)	1.98(3.35 – 1.18)	-1.59(-1.75 – -1.43)
Fiji	Both	27.15(35.12 – 18.43)	50.51(73.11 – 28.44)	23.36	7.25(9.42 – 4.92)	6.09(8.77 – 3.44)	-0.55(-0.88 – -0.21)
Finland	Both	1508.62(1778.46 – 1245.31)	1114.09(1356.76 – 875.43)	-394.53	21.24(24.94 – 17.69)	9.03(10.97 – 7.25)	-2.95(-3.06 – -2.85)
France	Both	35566.24(40887.21 – 30430.98)	32201.93(38715.11 – 26128.65)	-3364.31	43.71(50.16 – 37.67)	23.73(28.34 – 19.17)	-1.83(-1.87 – -1.78)
Gabon	Both	41.01(59.12 – 26.82)	74.83(110.49 – 51.03)	33.82	7.17(10.18 – 4.69)	7.12(10.47 – 4.99)	-0.01(-0.15 – 0.12)
Gambia	Both	14.83(20.48 – 9.58)	29.51(41.66 – 20.32)	14.68	4.33(5.91 – 2.77)	3.11(4.48 – 2.12)	-1.1(-1.82 – -0.37)
Georgia	Both	2025.83(2572.85 – 1578.92)	2706.64(3268.98 – 2208.63)	680.81	31.33(39.66 – 24.44)	46.02(55.66 – 37.52)	1.41(-0.19 – 3.03)
Germany	Both	48215.35(54901.8 – 41101.61)	36350.44(44146.64 – 29464.67)	-11864.91	37.64(42.75 – 32.26)	19.16(23.11 – 15.97)	-2.13(-2.5 – -1.76)
Ghana	Both	219.59(300.98 – 149.06)	528.7(783.95 – 360.12)	309.11	3.94(5.36 – 2.72)	3.45(5.21 – 2.32)	-0.41(-0.56 – -0.26)
Greece	Both	10575.58(11859.39 – 9278.81)	10949.5(12899.39 – 9281.99)	373.92	67.93(76.09 – 59.7)	46.88(54.59 – 40.34)	-1.24(-1.76 – -0.71)
Greenland	Both	14.85(19.39 – 9.67)	17.16(24.05 – 11.46)	2.31	44.74(57.35 – 29.21)	23.56(32.81 – 15.97)	-1.97(-2.38 – -1.55)

Grenada	Both	5.31(6.73 – 3.95)	8.01(10.02 – 5.96)	2.7	7.62(9.56 – 5.72)	6.75(8.54 – 5.04)	-0.22(-0.83 – 0.39)
Guam	Both	5.45(7.29 – 4.06)	13.86(17.76 – 10.35)	8.41	6.38(8.51 – 4.72)	6.58(8.4 – 4.91)	0.09(-0.28 – 0.46)
Guatemala	Both	78.89(99.03 – 62.45)	159.34(202.07 – 119.68)	80.45	2.51(3.16 – 1.96)	1.48(1.88 – 1.11)	-1.7(-2.46 – -0.94)
Guinea	Both	228.01(322.99 – 156.29)	434.88(650.94 – 282.86)	206.87	7.07(9.95 – 4.81)	8.05(11.98 – 5.25)	0.42(0.34 – 0.5)
Guinea-Bissau	Both	14.3(22.27 – 7.87)	29.33(44.22 – 19.04)	15.03	3.56(5.48 – 1.99)	3.76(5.66 – 2.47)	0.21(0 – 0.42)
Guyana	Both	21.43(27.01 – 16.97)	31.79(44.33 – 21.71)	10.36	5.75(7.29 – 4.53)	4.81(6.66 – 3.3)	-0.39(-1.26 – 0.49)
Haiti	Both	201.69(309.62 – 127.97)	283.48(413.01 – 168.48)	81.79	6.16(9.38 – 3.93)	4.07(6 – 2.45)	-1.29(-1.35 – -1.23)
Honduras	Both	43.18(60.89 – 30.55)	165.28(252.8 – 103.59)	122.1	2.18(3.04 – 1.52)	2.69(4.16 – 1.68)	0.75(0.59 – 0.9)
Hungary	Both	6285.84(7322.41 – 5272.82)	6760.03(8446.73 – 5428.59)	474.19	42.11(48.91 – 35.48)	36.36(44.73 – 29.27)	-0.38(-0.9 – 0.15)
Iceland	Both	102.29(118.76 – 86.42)	88.2(112.51 – 69.67)	-14.09	35.86(41.6 – 30.35)	15.09(19.24 – 12.04)	-2.8(-3.13 – -2.46)
India	Both	28135.58(36370.74 – 19208.5)	53664.93(69141.72 – 41655.34)	25529.35	6.51(8.46 – 4.43)	4.7(6.05 – 3.65)	-1.04(-1.42 – -0.65)
Indonesia	Both	6032.83(7958.32 – 4615.86)	16260.39(28369.01 – 10245.3)	10227.56	6.43(8.64 – 4.89)	7.05(12.55 – 4.44)	0.29(0.24 – 0.35)
Iran (Islamic Republic of)	Both	3109.98(4105.47 – 2230.76)	8311.46(10274.83 – 6261.59)	5201.48	11.62(15.3 – 8.32)	10.77(13.39 – 8.03)	-0.23(-0.46 – 0.01)
Iraq	Both	2805.95(3945.41 – 1908.63)	8248.03(12180.44 – 5397.02)	5442.08	36.74(51.54 – 24.95)	37.44(54.54 – 24.61)	0.1(-0.14 – 0.35)
Ireland	Both	1500.35(1723.95 – 1292.86)	1134.85(1395.6 – 893.68)	-365.5	35.77(41.02 – 30.79)	13.88(17.02 – 10.98)	-2.98(-3.29 – -2.66)

Israel	Both	1529.19(1794.11 – 1292.16)	2191.55(2678.17 – 1741.91)	662.36	31.23(36.71 – 26.46)	17.74(21.61 – 14.21)	-1.65(-2.08 – -1.22)
Italy	Both	52909.04(59839.72 – 45287.81)	37094.2(44360.35 – 30037.17)	-15814.84	58.51(66.09 – 50.22)	25.58(30.13 – 21.17)	-2.69(-2.93 – -2.45)
Jamaica	Both	177.08(215.8 – 145.13)	267.52(371.18 – 186.73)	90.44	9.83(11.98 – 8.09)	8.73(12.13 – 6.09)	-0.33(-2.31 – 1.69)
Japan	Both	29684.57(33124.49 – 26361.95)	39094.4(46855.89 – 31528.15)	9409.83	17.43(19.47 – 15.4)	10.59(12.46 – 8.87)	-1.54(-1.83 – -1.26)
Jordan	Both	367.1(487.54 – 267.55)	1544.58(2189.55 – 1058.05)	1177.48	28.95(38.36 – 21.18)	21.37(30.18 – 14.96)	-0.97(-1.14 – -0.8)
Kazakhstan	Both	2476.72(3456.24 – 1791.14)	2389.55(2931.49 – 1875.7)	-87.17	18.68(25.95 – 13.52)	12.55(15.48 – 9.82)	-1.18(-1.66 – -0.71)
Kenya	Both	178.48(265.78 – 113.84)	487.22(640.73 – 355.63)	308.74	2.39(3.56 – 1.52)	2.28(2.96 – 1.65)	-0.15(-0.25 – -0.05)
Kiribati	Both	1.28(1.59 – 0.99)	2.85(3.73 – 2.07)	1.57	3.33(4.13 – 2.57)	3.66(4.74 – 2.7)	0.31(0.24 – 0.37)
Kuwait	Both	88.85(106.94 – 71.41)	461.39(589.77 – 349.06)	372.54	15.82(19.33 – 12.48)	16.66(21.5 – 12.42)	-0.01(-0.98 – 0.97)
Kyrgyzstan	Both	448.92(544.05 – 349.8)	636.57(814.63 – 492.81)	187.65	14.85(18 – 11.54)	12.61(16.09 – 9.78)	-0.53(-1.55 – 0.5)
Lao People's Democratic Republic	Both	250.94(396.18 – 148.96)	409.16(620.1 – 263.46)	158.22	12.29(19.3 – 7.38)	9.42(14.44 – 6.05)	-0.85(-0.92 – -0.77)
Latvia	Both	1070.13(1234.82 – 928.1)	1189.45(1461.77 – 945.33)	119.32	29.66(34.04 – 25.72)	31.45(38.6 – 25.3)	0.23(-0.26 – 0.72)
Lebanon	Both	1521.12(2171.02 – 948.28)	3592.06(4811.76 – 2622.39)	2070.94	71.75(101 – 45.7)	58.87(78.57 – 43.13)	-0.6(-0.8 – -0.41)
Lesotho	Both	65.1(96.4 – 45.03)	138.69(212.47 – 78)	73.59	7.94(11.63 – 5.48)	12.73(19.2 – 7.4)	1.63(1.28 – 1.98)
Liberia	Both	42.83(63.73 – 28.29)	58.95(89.57 – 37.8)	16.12	3.76(5.55 – 2.52)	2.64(3.87 – 1.71)	-1.14(-1.32 – -0.97)

Libya	Both	658.53(961.82 – 441.31)	2045.49(2933.79 – 1413.26)	1386.96	36.49(53.18 – 24.35)	41(58.79 – 28.28)	0.4(0.16 – 0.63)
Lithuania	Both	1377.14(1594.67 – 1146.05)	1411.51(1689.23 – 1157.87)	34.37	30.39(35.01 – 25.33)	24.79(29.62 – 20.3)	-0.99(-3.41 – 1.49)
Luxembourg	Both	226.58(266.8 – 188.07)	208.33(257.3 – 163.27)	-18.25	41.15(48.57 – 33.95)	19.71(24.26 – 15.53)	-2.44(-2.99 – -1.89)
Madagascar	Both	234.04(313.96 – 168)	230.86(323.74 – 148.39)	-3.18	5.08(6.88 – 3.64)	2.34(3.37 – 1.54)	-2.46(-2.62 – -2.29)
Malawi	Both	718.7(934.9 – 537.75)	1670.86(2340.17 – 1104.04)	952.16	21.2(27.27 – 15.95)	24.5(33.56 – 16.71)	0.48(0.32 – 0.63)
Malaysia	Both	1442.98(2007.16 – 847.57)	3697.97(4942.14 – 2511.22)	2254.99	16.7(23.37 – 9.69)	13.73(18.46 – 9.18)	-0.7(-1.06 – -0.34)
Maldives	Both	10.28(15.26 – 6.77)	22.44(29.88 – 16.37)	12.16	14.06(20.65 – 9.17)	7.7(10.15 – 5.65)	-2.01(-2.27 – -1.75)
Mali	Both	497.75(657.73 – 356.24)	1459.7(2109.35 – 1017.25)	961.95	14.15(18.79 – 10.22)	18.99(27.13 – 13.12)	1.01(0.85 – 1.16)
Malta	Both	180.6(212.43 – 151.99)	173.52(217.64 – 139.6)	-7.08	41.62(48.9 – 35.04)	18.81(23.45 – 15.26)	-2.55(-2.8 – -2.3)
Marshall Islands	Both	1.25(1.91 – 0.82)	3.33(4.94 – 2.04)	2.08	7.39(11.13 – 4.84)	8.78(12.8 – 5.55)	0.59(0.42 – 0.77)
Mauritania	Both	51.43(74.45 – 35.99)	72.95(119.09 – 41.56)	21.52	5.17(7.51 – 3.62)	3.38(5.52 – 1.94)	-1.38(-1.57 – -1.19)
Mauritius	Both	185.44(212.46 – 160.34)	223.82(259.81 – 192.64)	38.38	25.97(29.83 – 22.34)	11.99(13.94 – 10.3)	-2.57(-3.38 – -1.75)
Mexico	Both	3222.95(3772.93 – 2733.92)	4641.76(5752.56 – 3685.54)	1418.81	8.16(9.57 – 6.9)	3.74(4.64 – 2.96)	-2.48(-3.07 – -1.88)
Micronesia (Federated States of)	Both	5.43(7.67 – 3.69)	9.53(13.46 – 6.16)	4.1	10.76(15.22 – 7.33)	11.38(15.89 – 7.45)	0.18(0.15 – 0.2)
Monaco	Both	24.89(37.02 – 15.03)	28.13(70.03 – 12.66)	3.24	36.47(54.26 – 21.83)	29.09(73.52 – 12.98)	-0.75(-0.79 – -0.71)

Mongolia	Both	109.43(157.07 – 67.23)	178.08(256.07 – 125.57)	68.65	10.32(14.89 – 6.37)	7.3(10.47 – 5.19)	-1.2(-1.66 – -0.73)
Montenegro	Both	205.63(267.93 – 153.65)	347.15(467.64 – 255.7)	141.52	32.36(42.25 – 24.11)	33.94(45.59 – 25.02)	0.34(0.1 – 0.59)
Morocco	Both	927.3(1257.97 – 644.94)	1943.24(2739.33 – 1313.43)	1015.94	6.68(9.03 – 4.56)	5.62(8.01 – 3.84)	-0.56(-0.75 – -0.37)
Mozambique	Both	300.67(437.64 – 204.06)	583.83(828.03 – 422.3)	283.16	5.53(7.93 – 3.72)	5.58(7.93 – 4.03)	0.04(-0.09 – 0.18)
Myanmar	Both	2708.75(3740.83 – 1967.62)	2654.5(4181.33 – 1817.51)	-54.25	12.22(16.83 – 8.99)	5.73(9.2 – 3.93)	-2.42(-2.49 – -2.35)
Namibia	Both	32.78(43.1 – 24.06)	61.3(80.46 – 45.45)	28.52	5.8(7.52 – 4.32)	5.04(6.62 – 3.82)	-0.43(-0.57 – -0.29)
Nauru	Both	0.64(0.93 – 0.39)	0.78(1.16 – 0.45)	0.14	13.49(19.43 – 8.2)	12.35(18.15 – 7.25)	-0.29(-0.34 – -0.24)
Nepal	Both	653.22(1026.89 – 412.53)	1104.89(2220.87 – 703.41)	451.67	7.93(12.47 – 4.92)	5.18(10.34 – 3.33)	-1.34(-1.46 – -1.22)
Netherlands	Both	10025.96(11312.74 – 8632.84)	8095.67(9890.69 – 6725.06)	-1930.29	49.93(56.16 – 43.07)	22.14(26.84 – 18.47)	-2.6(-3.08 – -2.12)
New Zealand	Both	978.24(1147.96 – 815.65)	1063.53(1320.11 – 838.27)	85.29	24.39(28.58 – 20.41)	12.27(15.16 – 9.77)	-2.2(-2.84 – -1.56)
Nicaragua	Both	29.9(38.26 – 22.08)	85.11(116.14 – 62.1)	55.21	2.04(2.62 – 1.49)	1.78(2.42 – 1.3)	-0.46(-0.8 – -0.11)
Niger	Both	47.06(75.69 – 30.55)	95.63(166.04 – 57.73)	48.57	1.95(3.13 – 1.24)	1.41(2.46 – 0.83)	-1.04(-1.18 – -0.9)
Nigeria	Both	279.47(428.78 – 186.63)	397.66(624.63 – 268.09)	118.19	0.68(1.03 – 0.46)	0.45(0.73 – 0.3)	-1.27(-1.38 – -1.16)
Niue	Both	0.16(0.21 – 0.11)	0.19(0.27 – 0.12)	0.03	7.57(10.21 – 5.02)	8.27(11.75 – 5.38)	0.27(0.19 – 0.35)
North Macedonia	Both	763.25(951.23 – 602.99)	1301.8(1708.76 – 936.19)	538.55	40.33(50.36 – 31.74)	37.94(49.84 – 27.47)	-0.17(-0.41 – 0.07)
Northern Mariana Islands	Both	1.61(2.66 – 1.07)	6.73(8.61 – 5.2)	5.12	8.51(14.12 – 5.89)	11.87(15.31 – 9.14)	1.13(0.66 – 1.6)

Norway	Both	2677.8(3071.23 – 2329.76)	1330.83(1616.8 – 1070.95)	-1346.97	38.83(44.33 – 34.08)	13(15.71 – 10.54)	-3.46(-3.65 – -3.28)
Oman	Both	44.9(66.71 – 29.17)	82.66(112.45 – 57.61)	37.76	7(10.24 – 4.46)	4.56(6.22 – 3.13)	-1.34(-1.84 – -0.84)
Pakistan	Both	16379.19(21364.89 – 12332.37)	27681.93(40572.13 – 19440.28)	11302.74	30.72(39.94 – 22.9)	24.89(36.4 – 17.55)	-0.68(-0.75 – -0.6)
Palau	Both	0.37(0.5 – 0.26)	0.74(1.04 – 0.51)	0.37	3.64(4.97 – 2.58)	2.97(4.12 – 2.09)	-0.66(-0.8 – -0.52)
Palestine	Both	248.34(351.14 – 168.36)	580.39(761.17 – 426.22)	332.05	29.98(42.31 – 20.37)	23.78(31.82 – 17.7)	-0.72(-0.98 – -0.47)
Panama	Both	62.2(74.76 – 51.63)	126.66(168.34 – 91.88)	64.46	4.33(5.22 – 3.58)	2.88(3.83 – 2.09)	-1.27(-1.63 – -0.91)
Papua New Guinea	Both	85.67(133.81 – 43.23)	238.26(389.91 – 126.87)	152.59	4.39(6.84 – 2.33)	4.17(6.6 – 2.27)	-0.14(-0.32 – 0.05)
Paraguay	Both	182.49(237.8 – 139.65)	454.69(637.95 – 309.88)	272.2	8.7(11.34 – 6.64)	8.16(11.41 – 5.6)	-0.09(-0.51 – 0.33)
Peru	Both	339.9(464.16 – 241.09)	779.16(1189.04 – 494.72)	439.26	3(4.12 – 2.1)	2.36(3.61 – 1.49)	-0.65(-1.59 – 0.29)
Philippines	Both	1957.59(2511.69 – 1529.05)	4814.14(6701.82 – 3606.22)	2856.55	6.76(8.71 – 5.22)	5.77(8.21 – 4.37)	-0.45(-0.62 – -0.29)
Poland	Both	21752.99(24125.35 – 19191.09)	30412.64(36248.49 – 25065.42)	8659.65	48.67(54.1 – 42.93)	41.86(49.81 – 34.61)	-0.46(-0.62 – -0.3)
Portugal	Both	3749.89(4402.27 – 3153.01)	4088.65(4972.56 – 3261.48)	338.76	26.37(30.84 – 22.25)	17.96(21.7 – 14.6)	-1.3(-1.59 – -1.01)
Puerto Rico	Both	387.08(495.64 – 297.11)	541(726.11 – 391.51)	153.92	10.65(13.59 – 8.18)	7.75(10.4 – 5.66)	-1.04(-1.98 – -0.08)
Qatar	Both	25.17(35.54 – 17.08)	112.69(174.24 – 68.27)	87.52	24.71(34.66 – 17.42)	12.1(18.82 – 7.49)	-2.4(-3.47 – -1.32)

Republic of Korea	Both	6909.28(8442.16 – 5283.75)	11638.28(15105.55 – 8309.33)	4729	24.72(30.36 – 18.84)	12.28(15.93 – 8.78)	-2.29(-2.46 – -2.13)
Republic of Moldova	Both	1094.17(1277.62 – 888.02)	1565.23(1832.12 – 1299.19)	471.06	23.81(27.86 – 19.23)	25.57(29.76 – 21.27)	0.17(-1.42 – 1.79)
Romania	Both	8658.35(10011.89 – 7298.6)	11539.2(13961.61 – 9337.38)	2880.85	29.63(34.14 – 25.11)	32.1(38.68 – 26.09)	0.25(-0.21 – 0.71)
Russian Federation	Both	51540.8(57514.01 – 45653.57)	53476.14(62661.96 – 44415.82)	1935.34	27.41(30.6 – 24.25)	21.89(25.6 – 18.23)	-0.71(-1.32 – -0.09)
Rwanda	Both	352.08(488.5 – 250.91)	586.73(930.33 – 376.71)	234.65	13.65(18.91 – 9.71)	10.64(16.54 – 7.04)	-0.79(-0.88 – -0.7)
Saint Kitts and Nevis	Both	3.16(4.06 – 2.42)	4.03(5.59 – 2.86)	0.87	8.11(10.34 – 6.29)	6.02(8.2 – 4.31)	-0.94(-1.38 – -0.5)
Saint Lucia	Both	11.27(13.83 – 8.99)	19.96(26.43 – 14.82)	8.69	13.02(16.1 – 10.38)	8.24(10.9 – 6.1)	-1.45(-1.92 – -0.98)
Saint Vincent and the Grenadines	Both	5.05(6.25 – 4)	10.03(12.74 – 7.8)	4.98	7.04(8.66 – 5.61)	6.91(8.77 – 5.38)	-0.05(-0.74 – 0.65)
Samoa	Both	8.56(12.62 – 6.19)	12.13(17.6 – 8.13)	3.57	10.08(14.55 – 7.36)	8.44(12.01 – 5.73)	-0.58(-0.64 – -0.52)
San Marino	Both	17.65(23.25 – 13.06)	14.23(21.43 – 8.78)	-3.42	49.41(65.46 – 36.59)	19.44(30.06 – 11.51)	-3.33(-3.76 – -2.9)
Sao Tome and Principe	Both	2.87(3.96 – 2.01)	7.09(10.58 – 4.49)	4.22	4.39(6.01 – 3.13)	6.17(9.36 – 3.88)	1.11(0.85 – 1.37)
Saudi Arabia	Both	397.93(621.32 – 241.11)	1430.02(2719.08 – 873.4)	1032.09	6.84(10.63 – 4.22)	6.75(12.57 – 4.2)	-0.05(-0.14 – 0.04)
Senegal	Both	202.5(276.91 – 138.22)	309.28(451.02 – 205.53)	106.78	6.02(8.27 – 4.11)	3.81(5.67 – 2.5)	-1.47(-1.76 – -1.19)
Serbia	Both	4145.98(5422.17 – 3146.26)	5596.04(7549.05 – 3982.53)	1450.06	36.59(47.81 – 27.74)	33.82(45.25 – 24.38)	-0.22(-0.36 – -0.07)
Seychelles	Both	17.92(22.84 – 13.83)	24.29(31.99 – 18.89)	6.37	31.67(40.34 – 24.47)	21.47(28.1 – 16.84)	-1.34(-1.75 – -0.94)

Sierra Leone	Both	115.64(156.33 – 79.75)	133.79(197.11 – 92.98)	18.15	5.71(7.68 – 3.96)	3.49(5.06 – 2.46)	-1.56(-1.75 – -1.36)
Singapore	Both	247.93(297.02 – 202.36)	366.57(452.19 – 290.92)	118.64	11.5(13.78 – 9.4)	4.21(5.19 – 3.34)	-3.34(-4.56 – -2.11)
Slovakia	Both	2201.39(2842.71 – 1731.1)	2416.38(3305.28 – 1753.04)	214.99	36.35(46.99 – 28.56)	24.76(33.52 – 18.08)	-1.23(-1.51 – -0.94)
Slovenia	Both	658.82(772.83 – 545.42)	912.36(1159.12 – 710.57)	253.54	26.56(31.22 – 22.06)	21.58(27.11 – 16.81)	-0.77(-1.64 – 0.11)
Solomon Islands	Both	14.02(21.92 – 6.53)	39.08(59.92 – 21.69)	25.06	9.8(15.15 – 4.74)	10.5(16.05 – 5.89)	0.21(0.08 – 0.35)
Somalia	Both	125.13(212.77 – 72.47)	243.89(411.33 – 133.47)	118.76	5.72(9.79 – 3.43)	4.18(6.99 – 2.33)	-0.99(-1.08 – -0.91)
South Africa	Both	2513.36(3430.28 – 1833.13)	3769.45(4565.46 – 2982.2)	1256.09	12.11(16.73 – 8.76)	7.95(9.66 – 6.29)	-1.39(-1.97 – -0.81)
South Sudan	Both	156.16(237.68 – 101.03)	169.87(271.45 – 105.36)	13.71	6.28(9.52 – 4.1)	4.79(7.38 – 2.97)	-0.85(-0.97 – -0.74)
Spain	Both	34253.96(38823.25 – 29354.05)	28692.61(35050.34 – 23198.46)	-5561.35	61.71(69.76 – 52.95)	30.21(36.34 – 24.65)	-2.32(-2.63 – -2.01)
Sri Lanka	Both	570.39(759.89 – 430.42)	878.75(1282.84 – 484.49)	308.36	5.83(7.76 – 4.39)	3.27(4.76 – 1.83)	-1.86(-2.55 – -1.18)
Sudan	Both	1394.58(2457.77 – 773)	2225.81(3246.09 – 1475.84)	831.23	15.17(26.71 – 8.54)	11.77(17.08 – 7.89)	-0.81(-0.84 – -0.78)
Suriname	Both	23.3(30.12 – 17.52)	47.22(66.25 – 31.28)	23.92	9.23(12.08 – 6.91)	7.28(10.26 – 4.81)	-0.67(-1.3 – -0.05)
Sweden	Both	3806.58(4430.08 – 3201.2)	3423.05(4223.42 – 2686.7)	-383.53	24.91(28.93 – 21.11)	15.22(18.71 – 12.1)	-1.56(-1.84 – -1.29)
Switzerland	Both	2090.33(2435.29 – 1751.79)	3234.22(3927.73 – 2620.24)	1143.89	20.22(23.47 – 16.93)	17.18(20.79 – 13.93)	-0.62(-1.56 – 0.32)
Syrian Arab Republic	Both	957.25(1302.16 – 683.57)	2084.28(3009.66 – 1365.51)	1127.03	18.68(25.36 – 13.45)	15.93(22.77 – 10.52)	-0.5(-0.78 – -0.22)

Taiwan (Province of China)	Both	4352.43(4948.12 – 3725.16)	7420.45(8646.49 – 6166.79)	3068.02	27.66(31.53 – 23.64)	17.33(20.11 – 14.43)	-1.4(-1.71 – -1.09)
Tajikistan	Both	199.68(303.28 – 130.14)	218.1(329.56 – 134.95)	18.42	7.22(11.03 – 4.71)	3.66(5.51 – 2.32)	-2.27(-2.61 – -1.94)
Thailand	Both	6751.77(8690.82 – 4821.66)	13030.7(18369.24 – 9171.83)	6278.93	20.47(26.24 – 14.47)	11.87(16.75 – 8.39)	-1.78(-2.02 – -1.55)
Timor-Leste	Both	14.35(23.37 – 8.45)	43.06(67.6 – 27.66)	28.71	5.73(9.1 – 3.4)	5.23(8.23 – 3.36)	-0.33(-0.43 – -0.24)
Togo	Both	108.98(148.34 – 76.92)	239.77(346.84 – 163.54)	130.79	9.42(12.72 – 6.74)	6.25(8.89 – 4.27)	-1.32(-1.43 – -1.21)
Tokelau	Both	0.12(0.17 – 0.07)	0.11(0.15 – 0.07)	-0.01	8.22(11.98 – 5.17)	7.21(10.26 – 4.5)	-0.44(-0.53 – -0.36)
Tonga	Both	5.19(8.37 – 3.19)	7.63(12.38 – 4.5)	2.44	9.53(15.52 – 5.85)	9.56(15.53 – 5.67)	-0.01(-0.26 – 0.23)
Trinidad and Tobago	Both	77.75(90.7 – 64.21)	134.14(179.38 – 93.63)	56.39	9.33(10.88 – 7.72)	6.7(8.94 – 4.67)	-0.76(-0.94 – -0.57)
Tunisia	Both	1401.78(1952.49 – 987.6)	3534.88(5181.83 – 2210.1)	2133.1	29.7(40.97 – 20.91)	27.03(39.48 – 16.89)	-0.35(-0.46 – -0.24)
Turkey	Both	15554.19(21307.27 – 10964.57)	28108.89(38202.93 – 20165.93)	12554.7	44.74(61.1 – 31.78)	29.46(40.03 – 21.19)	-1.32(-1.68 – -0.96)
Turkmenistan	Both	211.64(251.2 – 179)	296.27(406.62 – 210.09)	84.63	10.57(12.64 – 8.92)	6.85(9.4 – 4.88)	-1.37(-2.52 – -0.22)
Tuvalu	Both	0.62(0.85 – 0.41)	0.98(1.36 – 0.65)	0.36	8.61(11.76 – 5.79)	8.97(12.41 – 6.01)	0.13(0.1 – 0.17)
Uganda	Both	290.99(400.85 – 199.7)	549.4(788.76 – 397)	258.41	4.83(6.58 – 3.35)	3.98(5.58 – 2.9)	-0.68(-0.84 – -0.52)
Ukraine	Both	18661.48(25040.3 – 14407.52)	16935.04(23875.77 – 10969.26)	-1726.44	25.13(33.67 – 19.34)	21.8(30.89 – 14.06)	-0.39(-0.93 – 0.15)
United Arab Emirates	Both	125.38(241.33 – 67.42)	580.01(815.18 – 404.78)	454.63	27.07(52.66 – 14.13)	16.13(22.67 – 11.23)	-1.68(-2.36 – -1)

United Kingdom	Both	49982(56618.69 – 43516.74)	28486.08(34317.66 – 23272.06)	-21495.92	54.67(61.77 – 47.62)	21.28(25.31 – 17.62)	-2.97(-3.12 – -2.81)
United Republic of Tanzania	Both	873.39(1257.25 – 620.3)	1276.85(1871.09 – 851.51)	403.46	8.5(12.22 – 6.07)	5.24(7.58 – 3.53)	-1.56(-1.72 – -1.39)
United States of America	Both	82179.85(94513.71 – 69941.31)	107082.78(129617.13 – 87698.7)	24902.93	25.98(29.82 – 22.3)	18.13(21.81 – 14.89)	-1.18(-1.45 – -0.91)
United States Virgin Islands	Both	5.11(7.14 – 3.55)	6.42(9.53 – 4.05)	1.31	6.03(8.54 – 4.21)	3.38(4.98 – 2.16)	-1.93(-2.54 – -1.32)
Uruguay	Both	1532.72(1782.97 – 1293.98)	1519.46(1818.77 – 1240.01)	-13.26	38.88(45.08 – 32.91)	28.43(33.76 – 23.21)	-1.1(-1.65 – -0.55)
Uzbekistan	Both	479.78(662.68 – 328.74)	1600.94(2116.36 – 1146.74)	1121.16	4.08(5.59 – 2.79)	5.86(7.78 – 4.24)	0.98(0.31 – 1.66)
Vanuatu	Both	4.25(6.52 – 2.49)	9.87(15.16 – 6.01)	5.62	6.67(10.05 – 3.9)	5.43(8.28 – 3.29)	-0.7(-0.96 – -0.45)
Venezuela (Bolivarian Republic of)	Both	785.58(921.9 – 645.5)	1604.67(2224.52 – 1131.42)	819.09	8.41(9.88 – 6.92)	5.37(7.38 – 3.8)	-1.55(-2.52 – -0.57)
Viet Nam	Both	3038.45(4114.03 – 2081.82)	8296.23(10720.11 – 6133.94)	5257.78	7.69(10.3 – 5.3)	8.4(10.8 – 6.3)	0.29(0.23 – 0.36)
Yemen	Both	977.89(1550.91 – 563.05)	2582.09(3611.12 – 1700.79)	1604.2	19.48(30.82 – 11.24)	18.7(25.95 – 12.5)	-0.15(-0.26 – -0.04)
Zambia	Both	173.5(234.19 – 130.18)	424.09(836 – 233.99)	250.59	6.71(8.96 – 5.12)	6.64(12.16 – 3.78)	-0.02(-0.18 – 0.14)
Zimbabwe	Both	1170.58(1546.98 – 854.42)	2108.85(2892.51 – 1547.17)	938.27	30.82(40.54 – 22.78)	32.09(43.23 – 23.78)	0.16(0.03 – 0.29)
Afghanistan	Female	44.81(92.92 – 15.06)	84.29(162.17 – 34)	39.48	1.32(2.62 – 0.47)	1.49(2.74 – 0.65)	0.4(0.31 – 0.49)
Albania	Female	3.14(4.6 – 2.05)	8.65(12.64 – 5.57)	5.51	0.31(0.45 – 0.2)	0.37(0.53 – 0.24)	0.5(0.15 – 0.84)

Algeria	Female	26.78(41.03 – 17.49)	43.92(70.75 – 25.5)	17.14	0.67(1.17 – 0.39)	0.34(0.61 – 0.19)	-2.06(-2.35 – -1.77)
American Samoa	Female	0.14(0.26 – 0.09)	0.95(1.36 – 0.6)	0.81	1.11(2.1 – 0.72)	3.61(5.14 – 2.26)	3.88(2.77 – 5.01)
Andorra	Female	0.31(0.45 – 0.2)	0.55(0.81 – 0.34)	0.24	1.1(1.62 – 0.72)	0.71(1.05 – 0.44)	-1.49(-1.74 – -1.24)
Angola	Female	18.21(29.92 – 10.58)	54.83(83.81 – 32.54)	36.62	0.89(1.4 – 0.52)	0.79(1.17 – 0.48)	-0.37(-0.68 – -0.07)
Antigua and Barbuda	Female	0.86(1.19 – 0.63)	1.72(2.28 – 1.24)	0.86	2.87(3.86 – 2.07)	3.05(4.14 – 2.19)	-0.02(-0.8 – 0.76)
Argentina	Female	1210.07(1504.63 – 950.67)	1448.14(1814.4 – 1142.29)	238.07	6.69(8.29 – 5.28)	4.79(5.99 – 3.78)	-0.85(-1.18 – -0.52)
Armenia	Female	10.68(15.77 – 6.95)	15.82(22.34 – 10.92)	5.14	0.64(0.94 – 0.43)	0.62(0.86 – 0.43)	-0.03(-0.77 – 0.71)
Australia	Female	990.54(1199.29 – 802.89)	851.19(1122.62 – 622.11)	-139.35	9.07(10.98 – 7.36)	3.54(4.63 – 2.66)	-2.93(-3 – -2.85)
Austria	Female	526.83(652.4 – 423.43)	480.64(618.59 – 370.47)	-46.19	7.65(9.43 – 6.28)	5.25(6.5 – 4.16)	-1.12(-1.38 – -0.85)
Azerbaijan	Female	3.86(6.06 – 2.32)	6.31(10.63 – 3.67)	2.45	0.12(0.2 – 0.08)	0.1(0.16 – 0.06)	-0.78(-1.14 – -0.42)
Bahamas	Female	1.2(1.7 – 0.82)	3.06(4.11 – 2.06)	1.86	1.4(1.99 – 0.95)	1.39(1.87 – 0.94)	0.01(-0.75 – 0.77)
Bahrain	Female	3.7(5.17 – 2.44)	8.68(14.07 – 5.31)	4.98	5.15(7.06 – 3.39)	2.79(4.48 – 1.75)	-1.95(-2.36 – -1.54)
Bangladesh	Female	103.88(150.02 – 69.94)	184.36(324.99 – 114.56)	80.48	0.55(0.81 – 0.36)	0.28(0.5 – 0.18)	-2.06(-2.41 – -1.71)

Barbados	Female	1.54(2.17 – 1.06)	2.12(3.03 – 1.42)	0.58	0.97(1.31 – 0.69)	0.77(1.09 – 0.52)	-0.79(-1.27 – -0.3)
Belarus	Female	72.77(101.86 – 50.91)	57.3(80.46 – 37.75)	-15.47	0.94(1.3 – 0.66)	0.64(0.88 – 0.43)	-1.21(-2.45 – 0.05)
Belgium	Female	1128.31(1358.76 – 916.43)	798.33(1003.4 – 610.05)	-329.98	12.87(15.42 – 10.57)	6.71(8.17 – 5.33)	-2.28(-3.04 – -1.52)
Belize	Female	0.65(0.89 – 0.45)	2.21(3.01 – 1.63)	1.56	1.43(1.95 – 0.99)	1.5(2.06 – 1.09)	0.09(-0.48 – 0.67)
Benin	Female	5.13(7.82 – 3.01)	6.4(10.49 – 3.58)	1.27	0.5(0.77 – 0.3)	0.24(0.39 – 0.14)	-2.32(-2.52 – -2.11)
Bermuda	Female	2.05(2.91 – 1.44)	2.37(3.44 – 1.69)	0.32	5.81(8.23 – 4.08)	3.01(4.27 – 2.18)	-2.21(-3.21 – -1.2)
Bhutan	Female	0.87(1.53 – 0.46)	1.4(2.95 – 0.79)	0.53	0.75(1.35 – 0.4)	0.48(1.03 – 0.27)	-1.41(-1.52 – -1.3)
Bolivia (Plurinational State of)	Female	21.55(35.74 – 11.55)	49.42(81.95 – 28.58)	27.87	1.27(2.11 – 0.68)	1.02(1.7 – 0.6)	-0.69(-0.79 – -0.58)
Bosnia and Herzegovina	Female	129.91(171.15 – 98.12)	253.81(358.52 – 162.53)	123.9	5.6(7.35 – 4.29)	7.37(10.34 – 4.72)	0.97(0.6 – 1.33)
Botswana	Female	7.75(11.83 – 4.66)	13.37(20.32 – 8.39)	5.62	2.64(4.13 – 1.61)	1.73(2.57 – 1.11)	-1.45(-2.5 – -0.39)
Brazil	Female	3322.56(3982.4 – 2705.17)	6122.61(7459.13 – 4827.53)	2800.05	7.05(8.54 – 5.71)	4.38(5.34 – 3.45)	-1.52(-1.84 – -1.2)
Brunei Darussalam	Female	3.01(4.47 – 2.03)	5.29(7.45 – 3.65)	2.28	6.84(10.31 – 4.6)	3.31(4.68 – 2.25)	-2.33(-2.54 – -2.11)
Bulgaria	Female	300.37(381.05 – 226.24)	477.58(616.83 – 368.87)	177.21	4.53(5.68 – 3.47)	7.28(9.37 – 5.59)	1.52(0.46 – 2.59)

Burkina Faso	Female	5.11(7.65 – 3.04)	6.15(10.02 – 3.46)	1.04	0.24(0.37 – 0.14)	0.13(0.21 – 0.07)	-1.96(-2.16 – -1.75)
Burundi	Female	14.94(23.93 – 8.83)	9.47(13.86 – 6.27)	-5.47	1.2(1.9 – 0.72)	0.42(0.61 – 0.27)	-3.36(-3.51 – -3.21)
Cabo Verde	Female	0.05(0.07 – 0.03)	0.31(0.54 – 0.09)	0.26	0.04(0.05 – 0.02)	0.12(0.22 – 0.03)	4.08(3.27 – 4.89)
Cambodia	Female	31.14(51.42 – 16.58)	52.96(80.79 – 34.25)	21.82	1.28(2.07 – 0.69)	0.77(1.17 – 0.49)	-1.66(-1.72 – -1.59)
Cameroon	Female	8.35(13.07 – 5.16)	12.85(19.62 – 7.24)	4.5	0.48(0.79 – 0.28)	0.26(0.41 – 0.14)	-2(-2.15 – -1.84)
Canada	Female	2531.26(3002.02 – 2068.63)	2543.32(3248.48 – 1974.62)	12.06	13.9(16.52 – 11.46)	6.49(8.08 – 5.14)	-2.36(-2.99 – -1.73)
Central African Republic	Female	4.08(7.16 – 2)	5.3(9.11 – 2.79)	1.22	0.65(1.16 – 0.33)	0.43(0.71 – 0.24)	-1.32(-1.46 – -1.18)
Chad	Female	8.16(12.13 – 4.8)	9.31(14.53 – 5.57)	1.15	0.59(0.88 – 0.34)	0.4(0.62 – 0.24)	-1.28(-1.41 – -1.14)
Chile	Female	302.37(381.13 – 239.85)	396.14(505.51 – 313.79)	93.77	5.4(6.83 – 4.27)	2.91(3.68 – 2.3)	-2.01(-2.38 – -1.65)
China	Female	11797.44(15301.45 – 8566.36)	13982.41(19356.73 – 9889.27)	2184.97	3.1(4.05 – 2.27)	1.26(1.74 – 0.89)	-2.89(-3.17 – -2.61)
Colombia	Female	228.63(293.59 – 172.98)	291.86(381.7 – 217.62)	63.23	2.53(3.27 – 1.89)	0.96(1.26 – 0.72)	-3.18(-3.77 – -2.58)
Comoros	Female	0.94(1.53 – 0.52)	1.96(3.1 – 1.14)	1.02	1.02(1.66 – 0.55)	0.8(1.26 – 0.46)	-0.84(-0.94 – -0.74)
Congo	Female	2.67(4.62 – 1.28)	5.26(8.1 – 3.06)	2.59	0.42(0.73 – 0.21)	0.34(0.51 – 0.2)	-0.68(-0.95 – -0.41)

Cook Islands	Female	0.5(0.75 – 0.32)	0.66(1 – 0.44)	0.16	7.89(11.8 – 5.1)	5.07(7.63 – 3.37)	-1.42(-2.01 – -0.82)
Costa Rica	Female	22.14(28.26 – 16.78)	39.24(52.19 – 28.7)	17.1	2.52(3.23 – 1.9)	1.31(1.74 – 0.96)	-2.21(-2.65 – -1.78)
Croatia	Female	362.22(449.38 – 287.36)	575.21(747.93 – 425.32)	212.99	9.98(12.36 – 7.91)	11.49(14.72 – 8.61)	-0.9(-1.23 – -0.57)
Cuba	Female	342.39(439.11 – 261.33)	529.58(699.37 – 383.47)	187.19	6.68(8.54 – 5.09)	5.02(6.59 – 3.66)	0.35(-1.38 – 2.1)
Cyprus	Female	24.56(34.2 – 17.19)	35.55(47.45 – 25.06)	10.99	5.73(8.03 – 3.95)	3.35(4.46 – 2.4)	-0.84(-1.18 – -0.5)
Czechia	Female	771.04(956.68 – 594.71)	1047.77(1395 – 796.17)	276.73	9.48(11.7 – 7.38)	9.05(11.86 – 6.87)	-1.68(-2.02 – -1.35)
C么te d'Ivoire	Female	15.38(23.14 – 9.91)	32.3(51.29 – 19.17)	16.92	0.8(1.22 – 0.51)	0.59(0.93 – 0.36)	-0.32(-0.81 – 0.17)
Democratic People's Republic of Korea	Female	111.19(182.64 – 62.22)	180.3(306.69 – 111.57)	69.11	1.18(1.93 – 0.67)	0.93(1.56 – 0.58)	-0.77(-0.82 – -0.72)
Democratic Republic of the Congo	Female	35.39(57.05 – 20.91)	63.95(97.81 – 39.7)	28.56	0.4(0.62 – 0.25)	0.31(0.47 – 0.19)	-0.83(-0.93 – -0.74)
Denmark	Female	1108.96(1298.89 – 927.43)	875.69(1079.24 – 683.19)	-233.27	24.92(29.03 – 20.92)	13.39(16.33 – 10.66)	-1.93(-2.48 – -1.36)
Djibouti	Female	1.11(1.87 – 0.59)	4.09(7.09 – 2.17)	2.98	1.94(3.35 – 1.04)	1.62(2.77 – 0.88)	-0.56(-0.65 – -0.46)
Dominica	Female	1.07(1.48 – 0.73)	0.99(1.39 – 0.71)	-0.08	2.93(4.03 – 2.03)	2.21(3.08 – 1.58)	-0.92(-1.04 – -0.79)
Dominican Republic	Female	57.89(76.23 – 42.88)	134(187.38 – 93.27)	76.11	3.48(4.64 – 2.57)	2.6(3.64 – 1.81)	-0.96(-1.31 – -0.6)

Ecuador	Female	26.4(34.8 – 19.97)	61.47(85.99 – 41.07)	35.07	1(1.33 – 0.75)	0.72(1 – 0.48)	-1.26(-2.99 – 0.49)
Egypt	Female	154.81(218.02 – 107.4)	117.32(185.83 – 76.25)	-37.49	1.43(2.04 – 0.96)	0.58(0.96 – 0.34)	-2.89(-3.47 – -2.29)
El Salvador	Female	9.89(13.44 – 7.31)	24.96(34.78 – 17.49)	15.07	0.62(0.85 – 0.46)	0.71(0.99 – 0.5)	0.48(0.33 – 0.63)
Equatorial Guinea	Female	0.4(0.69 – 0.21)	0.94(1.57 – 0.51)	0.54	0.37(0.64 – 0.2)	0.32(0.54 – 0.18)	-0.42(-0.85 – 0.01)
Eritrea	Female	1.37(2.46 – 0.65)	2.65(4.35 – 1.46)	1.28	0.22(0.39 – 0.11)	0.18(0.29 – 0.1)	-0.78(-0.89 – -0.67)
Estonia	Female	53.49(70.93 – 39.07)	52.14(70.02 – 38.9)	-1.35	4.14(5.47 – 3.06)	3.37(4.5 – 2.51)	-0.82(-1.08 – -0.55)
Eswatini	Female	3.38(5.39 – 1.97)	5.12(8.59 – 2.46)	1.74	2.41(3.9 – 1.43)	1.75(2.92 – 0.9)	-1.02(-1.2 – -0.83)
Ethiopia	Female	28.81(51.66 – 13.04)	39.71(59.54 – 25.68)	10.9	0.28(0.49 – 0.13)	0.17(0.26 – 0.11)	-1.49(-1.67 – -1.32)
Fiji	Female	3.15(4.29 – 2.07)	5.68(8.3 – 3.09)	2.53	1.53(2.08 – 1.02)	1.27(1.85 – 0.7)	-0.61(-1.01 – -0.21)
Finland	Female	214.88(280.89 – 166.72)	169.11(217.9 – 125.72)	-45.77	5.18(6.65 – 4.06)	2.65(3.32 – 2.02)	-2.1(-2.18 – -2.02)
France	Female	3801.72(4657.44 – 2932.75)	3828.52(4987.5 – 2873.29)	26.8	7.72(9.34 – 6.04)	5.33(6.67 – 4.17)	-1.15(-1.59 – -0.71)
Gabon	Female	1.42(2.16 – 0.86)	2.3(3.49 – 1.34)	0.88	0.45(0.68 – 0.28)	0.41(0.63 – 0.24)	-0.29(-0.54 – -0.04)
Gambia	Female	0.24(0.34 – 0.15)	0.49(0.71 – 0.32)	0.25	0.16(0.23 – 0.1)	0.1(0.15 – 0.06)	-1.3(-1.78 – -0.82)

Georgia	Female	62.91(93.44 – 39.82)	44(61.53 – 31.49)	-18.91	1.68(2.49 – 1.07)	1.31(1.79 – 0.96)	-0.58(-1.66 – 0.52)
Germany	Female	8465.18(10239.45 – 6797.56)	6056.87(7648.13 – 4699.94)	-2408.31	11(13.17 – 8.93)	6.19(7.66 – 4.85)	-1.89(-2.27 – -1.51)
Ghana	Female	14.37(20.83 – 9.31)	33.74(52.86 – 21.23)	19.37	0.5(0.73 – 0.32)	0.39(0.61 – 0.24)	-0.81(-0.91 – -0.7)
Greece	Female	611.26(750.47 – 503.57)	972.78(1241.82 – 744.15)	361.52	7.48(9.09 – 6.22)	7.82(9.64 – 6.14)	0.16(-0.18 – 0.5)
Greenland	Female	3.29(4.19 – 2.35)	2.91(3.92 – 2.09)	-0.38	20.19(25.84 – 14.57)	8.7(12.06 – 6.16)	-2.62(-3.21 – -2.02)
Grenada	Female	0.86(1.17 – 0.6)	1(1.37 – 0.71)	0.14	2.12(2.87 – 1.51)	1.65(2.28 – 1.19)	-0.79(-1.37 – -0.21)
Guam	Female	0.27(0.4 – 0.19)	1.18(1.61 – 0.83)	0.91	0.64(0.93 – 0.45)	1.1(1.5 – 0.78)	1.59(0.93 – 2.25)
Guatemala	Female	10.28(14.08 – 7.47)	22.33(29.76 – 16.35)	12.05	0.64(0.91 – 0.45)	0.38(0.51 – 0.28)	-1.7(-3.38 – 0.01)
Guinea	Female	7.05(10.42 – 4.56)	9.81(15.36 – 5.98)	2.76	0.44(0.66 – 0.29)	0.38(0.6 – 0.23)	-0.54(-0.66 – -0.41)
Guinea-Bissau	Female	0.48(0.8 – 0.26)	0.98(1.52 – 0.62)	0.5	0.24(0.39 – 0.13)	0.25(0.39 – 0.16)	0.13(-0.06 – 0.32)
Guyana	Female	2.16(2.87 – 1.56)	3.11(4.56 – 2.08)	0.95	1.13(1.52 – 0.81)	0.91(1.34 – 0.61)	-0.6(-1.26 – 0.06)
Haiti	Female	29.72(54.75 – 13.42)	43.54(80.82 – 22.39)	13.82	1.79(3.24 – 0.83)	1.2(2.21 – 0.62)	-1.26(-1.37 – -1.15)

Honduras	Femal e	3.95(5.42 – 2.77)	15.07(22.4 – 10.03)	11.12	0.36(0.49 – 0.25)	0.43(0.65 – 0.29)	0.7(0.19 – 1.21)
Hungary	Femal e	816.82(1019.37 – 630.07)	1151.06(1477.53 – 885.94)	334.24	9.91(12.32 – 7.74)	11.66(14.84 – 9.06)	0.62(0.09 – 1.15)
Iceland	Femal e	22.89(28.12 – 17.79)	18.39(24.09 – 13.58)	-4.5	14.81(18 – 11.72)	6.02(7.71 – 4.59)	-3.1(-3.62 – -2.57)
India	Femal e	1601.08(2207.27 – 1147.8)	2792.32(3835.48 – 2079.41)	1191.2 4	0.78(1.07 – 0.55)	0.48(0.66 – 0.35)	-1.58(-2.17 – -0.98)
Indonesia	Femal e	203.3(317.74 – 116.47)	408.97(643.53 – 242.61)	205.67	0.44(0.69 – 0.25)	0.38(0.61 – 0.22)	-0.47(-0.55 – -0.38)
Iran (Islamic Republic of)	Femal e	107.66(151.8 – 73.41)	269.13(355.12 – 189.29)	161.47	0.86(1.22 – 0.59)	0.7(0.93 – 0.49)	-0.68(-0.86 – -0.5)
Iraq	Femal e	202.17(288.73 – 136.77)	463.5(669.36 – 304.28)	261.33	5.17(7.35 – 3.53)	4.18(5.95 – 2.81)	-0.65(-0.87 – -0.43)
Ireland	Femal e	366.39(435.68 – 311.56)	291.55(384.13 – 218.36)	-74.84	15.95(18.96 – 13.61)	6.78(8.88 – 5.14)	-2.66(-3.14 – -2.18)
Israel	Femal e	191.03(248.43 – 145.42)	237.87(320.59 – 173.01)	46.84	7.34(9.56 – 5.67)	3.5(4.59 – 2.6)	-2.4(-2.71 – -2.09)
Italy	Femal e	4612.47(5629.71 – 3740.31)	4098.49(5262.91 – 3083.79)	-513.98	9.15(11.07 – 7.47)	5.36(6.68 – 4.25)	-1.58(-1.81 – -1.36)
Jamaica	Femal e	31.38(42.13 – 23.4)	44.71(62.79 – 29.99)	13.33	3.3(4.42 – 2.48)	2.77(3.9 – 1.87)	-0.46(-0.81 – -0.11)
Japan	Femal e	2744.88(3334.54 – 2203.74)	3682.19(5210.51 – 2442.63)	937.31	2.85(3.46 – 2.29)	1.75(2.26 – 1.29)	-1.59(-1.84 – -1.34)
Jordan	Femal e	17.39(24.52 – 11.64)	52.75(79.43 – 32.89)	35.36	2.99(4.11 – 2)	1.8(2.71 – 1.11)	-1.57(-2.25 – -0.88)

Kazakhstan	Female	51.11(77.24 – 31.89)	39.7(53.73 – 28.78)	-11.41	0.65(0.96 – 0.41)	0.37(0.49 – 0.27)	-1.81(-2.46 – -1.15)
Kenya	Female	26.67(38.7 – 18.38)	48.23(70.39 – 31.47)	21.56	0.72(1.06 – 0.49)	0.44(0.66 – 0.29)	-1.54(-1.71 – -1.36)
Kiribati	Female	0.58(0.78 – 0.37)	1.37(1.87 – 0.8)	0.79	2.76(3.74 – 1.73)	3.13(4.22 – 1.79)	0.41(0.29 – 0.53)
Kuwait	Female	5.57(7.82 – 3.76)	12.42(18.2 – 8.13)	6.85	2.72(3.88 – 1.8)	1.06(1.58 – 0.68)	-3.03(-3.56 – -2.5)
Kyrgyzstan	Female	5.17(7.4 – 3.46)	12.53(18.04 – 8.57)	7.36	0.29(0.41 – 0.19)	0.41(0.58 – 0.28)	1.41(-0.81 – 3.67)
Lao People's Democratic Republic	Female	12.15(21.96 – 5.33)	17.05(27.44 – 10.14)	4.9	1.18(2.07 – 0.53)	0.76(1.23 – 0.46)	-1.39(-1.48 – -1.31)
Latvia	Female	54.77(70.49 – 39.84)	49.88(65.07 – 37.04)	-4.89	2.48(3.16 – 1.82)	2.37(3.03 – 1.79)	-0.18(-0.94 – 0.58)
Lebanon	Female	177.3(264.8 – 111.32)	453.56(641.33 – 309.18)	276.26	16.18(24.27 – 10.33)	13.79(19.39 – 9.47)	-0.46(-0.69 – -0.23)
Lesotho	Female	10.81(16.86 – 6.2)	16.91(27.86 – 9.37)	6.1	2.16(3.38 – 1.25)	2.91(4.71 – 1.57)	1.03(0.71 – 1.35)
Liberia	Female	2.1(3.29 – 1.34)	2.82(4.21 – 1.73)	0.72	0.41(0.63 – 0.26)	0.27(0.4 – 0.17)	-1.31(-1.55 – -1.06)
Libya	Female	4.9(7.23 – 3.18)	11.79(18.06 – 7.19)	6.89	0.58(0.85 – 0.37)	0.49(0.75 – 0.3)	-0.57(-0.78 – -0.35)
Lithuania	Female	40.63(52.85 – 30.37)	54.45(73.26 – 40.51)	13.82	1.54(2.01 – 1.16)	1.88(2.52 – 1.39)	0.58(-0.39 – 1.57)
Luxembourg	Female	35.15(44.97 – 25.79)	36.05(47.71 – 25.81)	0.9	11.31(14.34 – 8.42)	6.45(8.37 – 4.65)	-1.91(-2.34 – -1.48)

Madagascar	Female	19.26(28.63 – 12.66)	21.52(32.71 – 12.98)	2.26	0.85(1.28 – 0.55)	0.43(0.65 – 0.25)	-2.2(-2.29 – -2.12)
Malawi	Female	98.95(144.52 – 65.63)	162.44(272.53 – 92.51)	63.49	4.57(6.56 – 3.04)	3.96(6.64 – 2.27)	-0.46(-0.54 – -0.37)
Malaysia	Female	46.12(65.96 – 28.5)	93.06(135.73 – 60.39)	46.94	1.08(1.59 – 0.66)	0.79(1.19 – 0.5)	-1.09(-1.31 – -0.88)
Maldives	Female	0.3(0.53 – 0.12)	0.58(0.84 – 0.38)	0.28	0.93(1.63 – 0.43)	0.43(0.63 – 0.27)	-2.57(-3.06 – -2.09)
Mali	Female	20.41(29.18 – 14.04)	48.87(75.64 – 29.99)	28.46	1.01(1.45 – 0.71)	1.12(1.7 – 0.69)	0.31(0.19 – 0.44)
Malta	Female	22.08(28.31 – 16.67)	26.38(35.24 – 18.72)	4.3	9.29(11.84 – 7.03)	5.59(7.23 – 4.1)	-1.74(-2.48 – -1.01)
Marshall Islands	Female	0.08(0.13 – 0.05)	0.27(0.45 – 0.13)	0.19	0.93(1.54 – 0.56)	1.34(2.22 – 0.71)	1.19(1.11 – 1.27)
Mauritania	Female	2.6(4.24 – 1.64)	3.26(5.22 – 1.98)	0.66	0.48(0.79 – 0.3)	0.28(0.45 – 0.17)	-1.74(-1.99 – -1.48)
Mauritius	Female	9.62(12.65 – 7.1)	7.13(9.6 – 5.2)	-2.49	2.49(3.32 – 1.83)	0.7(0.94 – 0.52)	-4.11(-5.44 – -2.76)
Mexico	Female	465.97(585.23 – 365.89)	460.17(591.64 – 344.67)	-5.8	2.33(2.99 – 1.78)	0.69(0.9 – 0.51)	-3.83(-4.99 – -2.65)
Micronesia (Federated States of)	Female	0.61(0.89 – 0.36)	1.39(2.1 – 0.74)	0.78	2.42(3.47 – 1.43)	3.09(4.71 – 1.67)	0.79(0.73 – 0.84)
Monaco	Female	4.06(7.05 – 2.16)	4.96(13.02 – 1.61)	0.9	10.96(19 – 5.91)	9.97(26.6 – 3.23)	-0.31(-0.35 – -0.27)
Mongolia	Female	13.33(20.48 – 7.21)	10.62(16.43 – 6.76)	-2.71	2.33(3.59 – 1.26)	0.8(1.24 – 0.51)	-3.48(-4 – -2.96)

Montenegro	Female	31.8(42.41 – 22.89)	48.54(65.7 – 35.54)	16.74	9.05(12.11 – 6.59)	8.88(11.91 – 6.59)	0.04(-0.26 – 0.34)
Morocco	Female	5.93(8.41 – 3.76)	9.49(14.08 – 5.8)	3.56	0.08(0.12 – 0.05)	0.05(0.08 – 0.03)	-1.37(-1.42 – -1.31)
Mozambique	Female	37.99(53.93 – 24.53)	56.22(87.33 – 32.71)	18.23	1.26(1.8 – 0.83)	0.97(1.46 – 0.57)	-0.87(-0.95 – -0.79)
Myanmar	Female	517.8(866.48 – 279.97)	317.18(522.64 – 209.77)	-200.62	4.41(7.29 – 2.49)	1.2(2.02 – 0.79)	-4.13(-4.3 – -3.96)
Namibia	Female	12.48(17.18 – 8.69)	19.99(29.51 – 12.74)	7.51	3.96(5.34 – 2.72)	2.83(4.16 – 1.8)	-1.07(-1.21 – -0.94)
Nauru	Female	0.09(0.14 – 0.05)	0.15(0.24 – 0.07)	0.06	3.57(5.52 – 1.93)	4.02(6.42 – 2.01)	0.38(0.25 – 0.5)
Nepal	Female	148.84(236.85 – 103.47)	181.94(339.73 – 121.19)	33.1	3.72(5.83 – 2.53)	1.63(3.06 – 1.08)	-2.62(-2.67 – -2.56)
Netherlands	Female	1619.16(1909.62 – 1348.84)	1602.33(2002.8 – 1244.4)	-16.83	14.28(16.71 – 12.1)	8.36(10.43 – 6.63)	-1.68(-2.22 – -1.14)
New Zealand	Female	249.59(303.18 – 201.57)	214.12(271.45 – 162.93)	-35.47	11.33(13.61 – 9.2)	4.67(5.86 – 3.65)	-2.89(-3.9 – -1.88)
Nicaragua	Female	2.97(3.95 – 2.18)	8.3(11.34 – 5.84)	5.33	0.34(0.45 – 0.25)	0.3(0.41 – 0.21)	-0.41(-0.68 – -0.14)
Niger	Female	1.57(2.45 – 0.9)	3.43(5.5 – 1.82)	1.86	0.13(0.21 – 0.07)	0.08(0.13 – 0.04)	-1.44(-1.63 – -1.25)
Nigeria	Female	44.56(71.97 – 28.06)	46.31(72.48 – 27.89)	1.75	0.24(0.39 – 0.15)	0.1(0.17 – 0.06)	-2.72(-2.86 – -2.58)
Niue	Female	0.02(0.02 – 0.01)	0.02(0.03 – 0.01)	0	1.33(2.09 – 0.79)	1.49(2.32 – 0.8)	0.36(0.23 – 0.5)

North Macedonia	Female	70.62(93.83 – 53.18)	127.33(170.97 – 89.17)	56.71	7.03(9.36 – 5.34)	7.46(9.94 – 5.29)	0.19(-0.08 – 0.46)
Northern Mariana Islands	Female	0.08(0.2 – 0.05)	1.51(2.22 – 0.7)	1.43	1.03(2.4 – 0.59)	5.18(7.32 – 2.44)	5.3(4.71 – 5.89)
Norway	Female	522.62(638.61 – 414.71)	246.57(330.99 – 176.77)	-276.05	13.77(16.44 – 11.19)	4.56(5.87 – 3.43)	-3.53(-4 – -3.06)
Oman	Female	2.88(4.39 – 1.9)	3.7(5.32 – 2.38)	0.82	1.02(1.55 – 0.66)	0.47(0.67 – 0.3)	-2.52(-2.98 – -2.05)
Pakistan	Female	817.1(1146.02 – 582.14)	1121.59(1646.31 – 727.17)	304.49	3.35(4.74 – 2.41)	2.08(3.09 – 1.36)	-1.55(-1.67 – -1.44)
Palau	Female	0.01(0.01 – 0)	0.01(0.02 – 0.01)	0	0.12(0.18 – 0.08)	0.09(0.13 – 0.06)	-0.89(-0.95 – -0.84)
Palestine	Female	7.17(11.02 – 4.6)	9.55(14.25 – 6.05)	2.38	1.7(2.64 – 1.07)	0.85(1.31 – 0.53)	-2.27(-2.5 – -2.03)
Panama	Female	10.26(13.23 – 7.64)	19.76(27.52 – 13.53)	9.5	1.44(1.88 – 1.06)	0.86(1.2 – 0.59)	-1.59(-2.62 – -0.54)
Papua New Guinea	Female	16.57(25.44 – 9.26)	50.26(76.37 – 28.05)	33.69	1.66(2.5 – 0.94)	1.8(2.7 – 1)	0.28(0.02 – 0.55)
Paraguay	Female	21.66(28.75 – 15.39)	44.27(62.92 – 29.78)	22.61	1.93(2.57 – 1.37)	1.48(2.1 – 1)	-0.82(-1.29 – -0.35)
Peru	Female	28.9(40.5 – 20.4)	75.06(105.57 – 51.54)	46.16	0.48(0.67 – 0.33)	0.43(0.61 – 0.29)	-0.34(-1.77 – 1.1)
Philippines	Female	196.38(255.04 – 146.86)	356.62(475.93 – 263.78)	160.24	1.65(2.28 – 1.13)	0.9(1.21 – 0.67)	-2(-2.26 – -1.74)
Poland	Female	1643.7(1964.67 – 1375.23)	4391.17(5398.88 – 3501.14)	2747.47	6.48(7.71 – 5.45)	11.08(13.6 – 8.94)	1.75(1.09 – 2.41)

Portugal	Female	318.33(401.03 – 247.35)	222.29(281.74 – 171.57)	-96.04	4.07(5.09 – 3.2)	2.12(2.65 – 1.67)	-2.09(-2.54 – -1.64)
Puerto Rico	Female	72.95(101.29 – 50.5)	84.6(123.64 – 56.83)	11.65	3.73(5.17 – 2.59)	2.13(3.05 – 1.46)	-1.71(-2.06 – -1.37)
Qatar	Female	0.35(0.55 – 0.21)	1.39(2.23 – 0.79)	1.04	1.11(1.73 – 0.63)	0.54(0.9 – 0.31)	-2.23(-2.68 – -1.77)
Republic of Korea	Female	496.12(674.96 – 351.02)	885.38(1362.82 – 550.52)	389.26	3.44(4.94 – 2.4)	1.53(2.34 – 0.97)	-2.62(-2.9 – -2.34)
Republic of Moldova	Female	25.17(38.49 – 16.36)	25.25(35.56 – 18)	0.08	0.93(1.4 – 0.6)	0.72(1 – 0.52)	-1(-2.38 – 0.41)
Romania	Female	660.61(888.58 – 488.47)	798.14(1052.77 – 584.51)	137.53	4.19(5.59 – 3.1)	4.36(5.7 – 3.25)	0.19(-0.7 – 1.08)
Russian Federation	Female	931.69(1191.45 – 717.39)	1628.08(2034.64 – 1291.27)	696.39	0.82(1.04 – 0.64)	1.21(1.49 – 0.97)	1.22(0.37 – 2.07)
Rwanda	Female	94.05(146.29 – 57.35)	187.07(307.83 – 98.35)	93.02	6.78(10.59 – 4.19)	5.92(9.55 – 3.09)	-0.44(-0.52 – -0.37)
Saint Kitts and Nevis	Female	0.74(1.05 – 0.5)	0.73(1.03 – 0.5)	-0.01	3.41(4.76 – 2.34)	2.12(2.94 – 1.45)	-1.43(-2.68 – -0.17)
Saint Lucia	Female	1.96(2.57 – 1.41)	2.92(4.11 – 1.99)	0.96	4.11(5.41 – 2.95)	2.27(3.19 – 1.55)	-2.08(-3.01 – -1.14)
Saint Vincent and the Grenadines	Female	0.61(0.82 – 0.45)	0.75(1.04 – 0.53)	0.14	1.53(2.06 – 1.14)	1.06(1.47 – 0.75)	-1.27(-2.25 – -0.27)
Samoa	Female	0.04(0.06 – 0.03)	0.08(0.1 – 0.05)	0.04	0.1(0.15 – 0.07)	0.1(0.14 – 0.07)	0.02(-0.08 – 0.11)
San Marino	Female	2.02(2.84 – 1.37)	1.68(2.62 – 0.96)	-0.34	10.67(14.7 – 7.37)	4.49(7.01 – 2.51)	-2.92(-3.34 – -2.51)

Sao Tome and Principe	Female	0.09(0.13 – 0.06)	0.16(0.23 – 0.11)	0.07	0.26(0.38 – 0.17)	0.28(0.42 – 0.19)	0.28(0.05 – 0.5)
Saudi Arabia	Female	13.93(22.44 – 8.38)	42.42(68.98 – 24.18)	28.49	0.58(0.94 – 0.35)	0.49(0.8 – 0.28)	-0.52(-0.71 – -0.32)
Senegal	Female	3.89(5.84 – 2.36)	4.79(7.3 – 2.85)	0.9	0.25(0.37 – 0.15)	0.12(0.18 – 0.07)	-2.45(-2.69 – -2.21)
Serbia	Female	476.83(613.83 – 360.6)	763.19(1005.26 – 554.94)	286.36	7.78(10.09 – 5.89)	8.94(11.74 – 6.63)	0.47(0.12 – 0.83)
Seychelles	Female	0.91(1.33 – 0.56)	1.16(1.67 – 0.79)	0.25	2.78(4.06 – 1.74)	1.96(2.85 – 1.34)	-1.21(-1.61 – -0.82)
Sierra Leone	Female	4.42(6.51 – 2.61)	5.45(8.35 – 3.49)	1.03	0.45(0.65 – 0.27)	0.28(0.42 – 0.18)	-1.5(-1.67 – -1.34)
Singapore	Female	22.47(30.5 – 15.8)	34.35(51.45 – 22.56)	11.88	2(2.77 – 1.37)	0.75(1.12 – 0.5)	-3.04(-3.44 – -2.63)
Slovakia	Female	134.19(180.87 – 100.23)	185.82(252.2 – 130.88)	51.63	4.11(5.47 – 3.06)	3.72(5 – 2.61)	-0.36(-0.73 – 0.01)
Slovenia	Female	95.46(119.75 – 72.98)	165.24(213.37 – 121.52)	69.78	6.64(8.2 – 5.1)	7.63(9.76 – 5.72)	0.31(-0.31 – 0.95)
Solomon Islands	Female	1.09(1.82 – 0.53)	4.37(6.61 – 2.36)	3.28	1.59(2.64 – 0.82)	2.27(3.35 – 1.23)	1.14(0.9 – 1.39)
Somalia	Female	12.73(22.91 – 6.15)	30.71(53.4 – 15.43)	17.98	1.13(1.97 – 0.57)	0.97(1.69 – 0.5)	-0.49(-0.58 – -0.41)
South Africa	Female	463.38(605.49 – 351.88)	525.58(688.1 – 398.55)	62.2	3.82(5.08 – 2.84)	1.94(2.56 – 1.46)	-2.33(-2.43 – -2.23)
South Sudan	Female	9.38(17.06 – 5.07)	12.93(21.43 – 6.77)	3.55	0.96(1.76 – 0.52)	0.77(1.29 – 0.4)	-0.7(-0.77 – -0.62)

Spain	Female	1480.33(1871.66 – 1141.8)	1563.21(1938.92 – 1215.98)	82.88	5.01(6.19 – 3.9)	3.53(4.28 – 2.83)	-1(-1.34 – -0.66)
Sri Lanka	Female	14.4(20.41 – 10.06)	24.95(38.85 – 13.71)	10.55	0.32(0.47 – 0.21)	0.17(0.27 – 0.09)	-2.06(-2.51 – -1.61)
Sudan	Female	47.2(82.79 – 24.14)	57.08(93.84 – 33.79)	9.88	1.12(1.96 – 0.57)	0.67(1.11 – 0.4)	-1.64(-1.69 – -1.6)
Suriname	Female	2.55(3.46 – 1.82)	4.56(6.56 – 3.13)	2.01	1.94(2.66 – 1.39)	1.32(1.9 – 0.9)	-1.19(-1.59 – -0.8)
Sweden	Female	922.24(1153.78 – 730.77)	914.66(1202.43 – 682.76)	-7.58	11.04(13.3 – 8.94)	7.8(10.04 – 5.96)	-1.14(-1.47 – -0.81)
Switzerland	Female	364.94(452.74 – 288.6)	602.67(784.99 – 453.03)	237.73	6.33(7.71 – 5.15)	5.94(7.49 – 4.63)	-0.26(-1.33 – 0.81)
Syrian Arab Republic	Female	25.73(37.22 – 17.32)	38.82(61.09 – 24.13)	13.09	1.05(1.51 – 0.71)	0.64(0.98 – 0.4)	-1.57(-1.87 – -1.27)
Taiwan (Province of China)	Female	129.61(177.41 – 92.78)	140.65(198.84 – 99.93)	11.04	1.67(2.27 – 1.2)	0.64(0.91 – 0.46)	-2.99(-3.84 – -2.13)
Tajikistan	Female	3.28(5.18 – 1.96)	3.5(5.39 – 2.14)	0.22	0.2(0.32 – 0.12)	0.1(0.15 – 0.06)	-2.35(-2.7 – -2.01)
Thailand	Female	372.44(522.74 – 265.32)	408.56(627.42 – 268.87)	36.12	2.18(3.1 – 1.54)	0.67(1.02 – 0.44)	-3.76(-4 – -3.52)
Timor-Leste	Female	0.64(1.09 – 0.3)	1.33(2.21 – 0.77)	0.69	0.54(0.94 – 0.26)	0.32(0.54 – 0.19)	-1.66(-1.85 – -1.46)
Togo	Female	7.45(11.6 – 4.27)	14.85(24.88 – 8.46)	7.4	1.25(1.94 – 0.71)	0.7(1.13 – 0.4)	-1.86(-1.95 – -1.77)
Tokelau	Female	0.01(0.02 – 0.01)	0.01(0.02 – 0.01)	0	1.59(2.49 – 0.98)	1.58(2.47 – 0.82)	0(-0.06 – 0.06)

Tonga	Female	0.37(0.54 – 0.23)	0.5(0.81 – 0.25)	0.13	1.29(1.92 – 0.8)	1.18(1.89 – 0.59)	-0.27(-0.38 – -0.16)
Trinidad and Tobago	Female	10.69(13.99 – 8.18)	14.32(20.08 – 9.98)	3.63	2.47(3.27 – 1.89)	1.39(1.95 – 0.98)	-1.94(-2.76 – -1.11)
Tunisia	Female	25.29(35.94 – 16.59)	48.67(76.37 – 29.54)	23.38	1.12(1.6 – 0.73)	0.72(1.14 – 0.43)	-1.45(-1.55 – -1.35)
Turkey	Female	496.2(710.91 – 330.7)	726.88(1011.19 – 515.53)	230.68	2.59(3.7 – 1.72)	1.45(2.02 – 1.02)	-1.88(-2.21 – -1.55)
Turkmenistan	Female	8.52(11.67 – 5.99)	6.06(8.59 – 4.1)	-2.46	0.73(1.01 – 0.52)	0.24(0.34 – 0.16)	-3.8(-4.99 – -2.59)
Tuvalu	Female	0.08(0.13 – 0.05)	0.13(0.2 – 0.07)	0.05	2.04(3.14 – 1.23)	2.23(3.4 – 1.25)	0.31(0.24 – 0.38)
Uganda	Female	48.95(73.53 – 29.89)	106.24(156.15 – 72.03)	57.29	1.69(2.57 – 1.03)	1.46(2.17 – 0.99)	-0.51(-0.73 – -0.3)
Ukraine	Female	281.61(398.2 – 189.47)	283.2(421.17 – 165.68)	1.59	0.67(0.96 – 0.45)	0.68(1.04 – 0.4)	-0.08(-1.16 – 1.01)
United Arab Emirates	Female	4.63(9.1 – 2.09)	14.07(20.47 – 9.62)	9.44	3.21(6.34 – 1.44)	2.81(4.2 – 1.74)	-0.35(-1.59 – 0.92)
United Kingdom	Female	12081.38(13896.02 – 10249.35)	8152.86(10067.24 – 6585.05)	-3928.52	23.22(26.51 – 19.85)	11.4(13.76 – 9.31)	-2.28(-2.5 – -2.06)
United Republic of Tanzania	Female	121.6(171.89 – 83.99)	203.86(301.49 – 127.88)	82.26	2.45(3.5 – 1.67)	1.62(2.42 – 1.03)	-1.34(-1.45 – -1.23)
United States of America	Female	20045.51(23562.9 – 16787.32)	24210.16(29589.27 – 18934.64)	4164.65	11.15(13 – 9.48)	7.65(9.24 – 6.07)	-1.28(-1.41 – -1.14)

United States Virgin Islands	Female	0.74(1.1 – 0.5)	0.73(1.23 – 0.42)	-0.01	1.63(2.44 – 1.09)	0.74(1.25 – 0.42)	-2.59(-2.9 – -2.28)
Uruguay	Female	117.22(150.84 – 89.61)	161.75(201.12 – 127.88)	44.53	5.43(6.93 – 4.19)	5.72(6.93 – 4.61)	0.13(-0.2 – 0.47)
Uzbekistan	Female	5.53(8.21 – 3.41)	21.4(30.48 – 14.35)	15.87	0.08(0.12 – 0.05)	0.13(0.19 – 0.09)	1.44(0.92 – 1.97)
Vanuatu	Female	0.15(0.24 – 0.08)	0.4(0.62 – 0.21)	0.25	0.52(0.83 – 0.31)	0.46(0.73 – 0.25)	-0.41(-0.77 – -0.05)
Venezuela (Bolivarian Republic of)	Female	134.38(173.78 – 102.01)	271.72(382.53 – 180.88)	137.34	2.65(3.44 – 2.01)	1.63(2.28 – 1.09)	-1.64(-2.17 – -1.1)
Viet Nam	Female	88.04(123.42 – 62.06)	100.83(147.83 – 66.51)	12.79	0.39(0.55 – 0.27)	0.19(0.28 – 0.13)	-2.31(-2.4 – -2.22)
Yemen	Female	81.95(134.44 – 44.99)	194.91(303.49 – 120.81)	112.96	3.3(5.25 – 1.86)	2.78(4.3 – 1.69)	-0.55(-0.64 – -0.46)
Zambia	Female	42.72(59.82 – 27.96)	80.97(157.2 – 37.49)	38.25	3.89(5.38 – 2.54)	2.82(5.28 – 1.3)	-1.05(-1.17 – -0.93)
Zimbabwe	Female	188.43(276.41 – 120.59)	279.44(472.57 – 147.1)	91.01	10.74(15.83 – 6.68)	8.8(14.28 – 4.72)	-0.61(-0.95 – -0.27)
Afghanistan	Male	615.57(1181.64 – 308.75)	910.42(1429.32 – 493.94)	294.85	16.6(31.5 – 8.33)	21.69(33.96 – 12.07)	0.87(0.81 – 0.93)
Albania	Male	47.83(64.6 – 35.07)	96.66(142.85 – 64.98)	48.83	5.6(7.67 – 4.13)	4.58(6.78 – 3.07)	-0.7(-1.26 – -0.14)
Algeria	Male	1274.51(1684.71 – 861.41)	2851.9(3909.92 – 2018.81)	1577.39	23.55(31.22 – 15.86)	17.3(23.71 – 12.17)	-1.01(-1.12 – -0.91)
American Samoa	Male	1.73(2.31 – 1.28)	3.65(4.89 – 2.75)	1.92	14.8(20.04 – 10.79)	14.53(19.39 – 11.03)	0.02(-0.29 – 0.33)

Andorra	Male	27.88(41.24 – 18.12)	36.69(54.07 – 22.66)	8.81	95.07(138.93 – 62.16)	46.95(69.01 – 29.17)	-2.37(-2.62 – -2.11)
Angola	Male	341.97(515.62 – 224.61)	891.75(1266.69 – 632.09)	549.78	18.56(27.6 – 12.52)	17.86(25.05 – 12.61)	-0.11(-0.33 – 0.11)
Antigua and Barbuda	Male	3.39(4.3 – 2.61)	5.93(7.54 – 4.47)	2.54	14.49(18.36 – 11.15)	11.8(15.13 – 8.86)	-0.56(-2.21 – 1.11)
Argentina	Male	8624.91(10106.4 – 7107.65)	6978.04(8437.24 – 5601.92)	-1646.87	58.21(68.32 – 48.01)	28.02(33.87 – 22.41)	-2.31(-2.95 – -1.67)
Armenia	Male	1431.37(1734.69 – 1165.22)	2222.65(2691.15 – 1794.58)	791.28	123.06(148.1 – 100.54)	120.3(145.84 – 97.26)	-0.03(-0.8 – 0.74)
Australia	Male	3411.94(4028.5 – 2870.91)	2616.74(3337.67 – 2058.52)	-795.2	37.89(45.08 – 31.75)	12.41(15.57 – 9.85)	-3.6(-3.73 – -3.47)
Austria	Male	2923.49(3434.44 – 2479.4)	2751.63(3306.47 – 2238.52)	-171.86	64.37(75.79 – 54.55)	34.14(40.8 – 27.87)	-1.99(-2.72 – -1.25)
Azerbaijan	Male	961.64(1344.61 – 696.56)	1705.63(2445.99 – 1136.05)	743.99	46.02(63.9 – 33.98)	36.85(51.78 – 25.18)	-0.72(-1.02 – -0.41)
Bahamas	Male	7.47(9.59 – 5.59)	18.86(24.46 – 13.23)	11.39	11.19(14.41 – 8.35)	10.31(13.25 – 7.22)	-0.21(-0.65 – 0.24)
Bahrain	Male	75.52(99.99 – 54.89)	207.23(315.71 – 132.73)	131.71	96.24(128.27 – 69.4)	50.09(71.96 – 32.79)	-2.02(-2.6 – -1.45)
Bangladesh	Male	4506.86(7200.74 – 2707.01)	8294.43(17223.63 – 4622.58)	3787.57	18.3(29.34 – 10.93)	12.2(25.28 – 6.89)	-1.17(-1.61 – -0.72)
Barbados	Male	20.58(25.34 – 16.26)	27.84(38.09 – 19.1)	7.26	16.26(19.84 – 12.83)	11.74(15.98 – 8.1)	-0.91(-1.53 – -0.29)
Belarus	Male	4048.49(4604.01 – 3506.96)	3750.2(4595.09 – 2918.05)	-298.29	86.37(98.36 – 74.8)	61.26(75.15 – 47.76)	-1.06(-1.74 – -0.38)
Belgium	Male	6594.77(7562.63 – 5631.13)	4380.21(5311.26 – 3609.71)	-2214.56	103.18(118.81 – 87.79)	41.28(49.63 – 34.25)	-3.03(-4.06 – -1.99)
Belize	Male	4.62(5.6 – 3.78)	15.06(19.1 – 11.42)	10.44	10.24(12.44 – 8.34)	10.31(13.07 – 7.84)	0.19(-0.51 – 0.91)

Benin	Male	84.59(125.38 – 58.98)	107.25(151.4 – 72.47)	22.66	8.93(13.19 – 6.25)	4.64(6.65 – 3.16)	-2.09(-2.27 – -1.91)
Bermuda	Male	13.68(17.66 – 10.47)	20.25(26.35 – 14.97)	6.57	51.51(66.48 – 39.31)	32.84(42.72 – 24.35)	-1.53(-2.25 – -0.81)
Bhutan	Male	7.97(14.39 – 3.72)	17.86(41.48 – 10.01)	9.89	8.09(14.47 – 3.84)	6.26(14.51 – 3.49)	-0.79(-0.85 – -0.72)
Bolivia (Plurinational State of)	Male	194.39(283.92 – 131.16)	396.72(577.51 – 263.67)	202.33	13.82(20.47 – 9.26)	9.72(14.02 – 6.39)	-1.12(-1.18 – -1.05)
Bosnia and Herzegovina	Male	950.7(1209.01 – 749.97)	1903.35(2571.38 – 1352.45)	952.65	53.86(67.93 – 42.97)	67.16(90.54 – 47.75)	0.74(0.35 – 1.14)
Botswana	Male	55.91(77.87 – 37.8)	110.94(163.7 – 74.66)	55.03	23.98(33 – 16.37)	19.18(27.1 – 12.97)	-0.67(-0.96 – -0.38)
Brazil	Male	14173.41(15964.22 – 12390.8)	19936.85(24147.23 – 16395.55)	5763.44	35.65(40.37 – 31.2)	18.12(22.01 – 14.79)	-2.1(-2.46 – -1.73)
Brunei Darussalam	Male	17.73(24.55 – 12.64)	29.45(39.14 – 21.15)	11.72	37.03(50.91 – 26.34)	19.52(25.69 – 14.01)	-2.09(-2.5 – -1.68)
Bulgaria	Male	3849.74(4567.07 – 3206.92)	4546.1(5770.01 – 3576.21)	696.36	63.56(75.02 – 53.32)	73.83(94.14 – 58.03)	0.52(-0.02 – 1.06)
Burkina Faso	Male	104.45(152.01 – 69.34)	184.65(261.57 – 128.91)	80.2	4.94(7.1 – 3.3)	4.04(5.8 – 2.83)	-0.65(-0.75 – -0.55)
Burundi	Male	171.23(268.63 – 104.9)	159.81(243.12 – 99.74)	-11.42	16.8(26.18 – 10.28)	6.6(9.95 – 4.31)	-2.98(-3.08 – -2.88)
Cabo Verde	Male	1.25(3.68 – 0.67)	16.13(23.84 – 7.86)	14.88	1.37(3.95 – 0.75)	8.51(12.52 – 4.13)	6.11(4 – 8.25)
Cambodia	Male	499.05(746.14 – 344.96)	1261.56(1905.53 – 848.16)	762.51	28.01(41.98 – 19.46)	27.03(41.3 – 18)	-0.09(-0.2 – 0.03)
Cameroon	Male	221.46(301.63 – 161.28)	497.38(709.16 – 323.46)	275.92	9.79(13.1 – 7.18)	7.59(10.85 – 4.93)	-0.83(-0.98 – -0.69)
Canada	Male	10426.36(12035.72 – 8901.76)	8371.69(10358.76 – 6628.27)	-2054.67	72.76(83.63 – 61.97)	24.58(30.34 – 19.46)	-3.41(-3.73 – -3.1)

Central African Republic	Male	85.05(130.89 – 51.27)	114.79(169.42 – 73.61)	29.74	16.41(24.7 – 10.38)	11.84(17.34 – 8.01)	-1.06(-1.19 – -0.93)
Chad	Male	110.64(156.27 – 68.14)	212.67(316.13 – 127.73)	102.03	8.32(11.72 – 5.1)	7.06(10.51 – 4.25)	-0.53(-0.64 – -0.42)
Chile	Male	1110.78(1330.37 – 890.69)	1525.57(1881.78 – 1201.74)	414.79	24.1(28.9 – 19.17)	13.08(16.17 – 10.28)	-1.92(-2.61 – -1.23)
China	Male	190848.81(240797.23 – 119458.36)	346002.73(476137.27 – 253304.48)	155153.92	52.05(65.84 – 33.09)	35.83(49.57 – 26.48)	-1.21(-1.49 – -0.92)
Colombia	Male	1097.18(1313.17 – 900.58)	1316.56(1764.51 – 981.57)	219.38	13.27(16.11 – 10.89)	5.37(7.21 – 4.02)	-2.96(-3.87 – -2.04)
Comoros	Male	12.05(19.86 – 7.19)	21.55(37.96 – 10.27)	9.5	13.8(22.35 – 8.25)	10.78(19.22 – 5.21)	-0.84(-0.98 – -0.7)
Congo	Male	85.55(124.36 – 50.53)	201.09(294.43 – 134.4)	115.54	19.17(28.05 – 11.85)	17.49(25.58 – 11.97)	-0.27(-0.44 – -0.1)
Cook Islands	Male	1.37(1.84 – 0.98)	2.37(3.3 – 1.66)	1	20.63(27.63 – 14.65)	18.6(26.1 – 13.08)	-0.31(-0.57 – -0.06)
Costa Rica	Male	184.15(216.41 – 154.58)	352.06(436.89 – 272.72)	167.91	22.54(26.52 – 18.84)	14.1(17.52 – 10.92)	-1.46(-1.85 – -1.07)
Croatia	Male	2212.07(2626.29 – 1867.12)	2893.64(3581.61 – 2262.03)	681.57	97.8(116 – 82.05)	74.44(91.96 – 58.62)	-0.33(-0.48 – -0.17)
Cuba	Male	2204.7(2545.33 – 1881.28)	3898.62(4730.46 – 3081.93)	1693.92	43.73(50.55 – 37.34)	42.46(51.63 – 33.52)	-0.78(-1.14 – -0.41)
Cyprus	Male	293.93(401.82 – 216.65)	525.37(732.49 – 373.56)	231.44	93.32(127.76 – 67.08)	53.89(75.64 – 38.44)	0.04(-0.11 – 0.18)
Czechia	Male	5309.01(6327.93 – 4428.91)	5456.33(7007.25 – 4242.64)	147.32	91.54(108.83 – 76.95)	55.91(71.65 – 43.36)	-1.77(-2 – -1.54)
C么te d'Ivoire	Male	188.06(264.17 – 123.87)	472.06(722 – 299.1)	284	9.56(13.01 – 6.38)	8.7(12.67 – 5.71)	-1.56(-1.86 – -1.25)
Democratic People's Republic of Korea	Male	2146.66(2941.5 – 1471.75)	3856.29(5823.64 – 2683.39)	1709.63	35.17(48.42 – 24.09)	27.81(41.75 – 19.79)	-0.75(-0.78 – -0.72)

Democratic Republic of the Congo	Male	635.6(920.18 – 406.03)	1224.76(1807.22 – 800.68)	589.16	8.77(12.78 – 5.7)	7.47(11.19 – 4.85)	-0.53(-0.72 – -0.34)
Denmark	Male	3087.73(4021.18 – 2453.25)	2755.02(3285.51 – 2304.18)	-332.71	88.61(114.44 – 70.8)	48.32(57.19 – 40.48)	-1.91(-2.99 – -0.81)
Djibouti	Male	11.28(18.3 – 6.71)	51.88(89.18 – 28.17)	40.6	19.42(30.32 – 12.09)	18.22(30.54 – 10.31)	-0.21(-0.34 – -0.09)
Dominica	Male	4.22(6.05 – 2.92)	5.62(8.4 – 3.78)	1.4	17.82(25.09 – 12.44)	14.12(21.05 – 9.52)	-0.75(-0.89 – -0.61)
Dominican Republic	Male	155.36(203.24 – 111.27)	433.63(663.21 – 266.11)	278.27	9.06(11.97 – 6.47)	9.28(14.22 – 5.76)	0.11(-0.72 – 0.95)
Ecuador	Male	199.46(241.08 – 159.72)	459(620.54 – 324.77)	259.54	8.29(10.05 – 6.63)	6.16(8.31 – 4.38)	-0.72(-2.68 – 1.27)
Egypt	Male	18317.71(23491.94 – 9710.92)	20635.53(36046.26 – 13901.82)	2317.82	122.26(155.74 – 73.82)	62.25(110.73 – 41.31)	-2.22(-2.69 – -1.75)
El Salvador	Male	42.63(59.2 – 29.94)	98.03(138.93 – 68.59)	55.4	3.11(4.33 – 2.18)	3.85(5.47 – 2.69)	0.73(-0.26 – 1.73)
Equatorial Guinea	Male	11.9(18.14 – 7.2)	28.62(45.64 – 17.12)	16.72	13.73(20.69 – 8.59)	13.94(22.67 – 8.33)	0.06(-0.23 – 0.34)
Eritrea	Male	53.18(82.04 – 30.66)	92.86(146.5 – 56.19)	39.68	10.39(15.89 – 6.08)	7.98(12.48 – 5.05)	-0.87(-0.98 – -0.76)
Estonia	Male	498.9(586.98 – 420.65)	490.49(593.86 – 394.3)	-8.41	65.68(77.46 – 55.4)	48.14(58.15 – 38.77)	-0.99(-1.53 – -0.45)
Eswatini	Male	13.63(19.96 – 9.01)	21.29(31.6 – 12.88)	7.66	11.97(17.21 – 8.05)	9.92(14.38 – 6.19)	-0.58(-0.71 – -0.44)
Ethiopia	Male	627.06(1029.83 – 327.76)	765.72(1334.86 – 444.26)	138.66	5.97(9.71 – 3.13)	3.71(6.42 – 2.18)	-1.52(-1.7 – -1.34)
Fiji	Male	23.99(31.67 – 15.76)	44.83(65.14 – 23.55)	20.84	13.45(17.66 – 8.79)	12.01(17.41 – 6.42)	-0.35(-0.72 – 0.02)
Finland	Male	1293.74(1517.49 – 1063.13)	944.98(1168.77 – 734.82)	-348.76	46.04(54.2 – 37.68)	16.52(20.21 – 13.07)	-3.56(-3.68 – -3.44)

France	Male	31764.51(36654.99 – 27475.63)	28373.4(34297.18 – 22758.7)	-3391.11	92.75(106.74 – 79.67)	46.12(55.34 – 37.05)	-2.14(-2.19 – -2.1)
Gabon	Male	39.59(57.49 – 25.41)	72.53(107.74 – 49.45)	32.94	15.63(22.29 – 10.15)	15.11(22.42 – 10.57)	-0.09(-0.26 – 0.09)
Gambia	Male	14.59(20.21 – 9.4)	29.02(41.18 – 19.97)	14.43	8.28(11.37 – 5.23)	6.47(9.39 – 4.39)	-0.8(-1.47 – -0.14)
Georgia	Male	1962.92(2496.86 – 1533.27)	2662.64(3197.89 – 2171.77)	699.72	77.19(97.66 – 59.86)	110.79(133.42 – 90.31)	1.35(-0.32 – 3.05)
Germany	Male	39750.17(45327.09 – 33991.43)	30293.56(36751.98 – 24729.03)	-9456.61	82.24(93.85 – 70.17)	34.68(41.68 – 28.6)	-2.78(-3.16 – -2.41)
Ghana	Male	205.21(282.36 – 138.69)	494.96(750.18 – 332.46)	289.75	7.82(10.77 – 5.4)	7.84(12.06 – 5.15)	0.03(-0.13 – 0.19)
Greece	Male	9964.32(11152.78 – 8773.37)	9976.73(11711.76 – 8383.49)	12.41	141.19(157.91 – 124.02)	92.7(108 – 79.56)	-1.4(-1.93 – -0.87)
Greenland	Male	11.57(15.61 – 6.78)	14.26(20.62 – 8.66)	2.69	73.9(98.59 – 42.84)	37.06(53.66 – 22.65)	-2.15(-2.44 – -1.85)
Grenada	Male	4.45(5.7 – 3.31)	7.01(8.8 – 5.22)	2.56	15(19.13 – 11.25)	13.35(17.12 – 9.94)	-0.14(-0.97 – 0.7)
Guam	Male	5.18(6.93 – 3.85)	12.68(16.43 – 9.37)	7.5	11.87(16.01 – 8.73)	12.33(15.91 – 9.04)	0.11(-0.36 – 0.58)
Guatemala	Male	68.61(85.45 – 54.11)	137.01(174.49 – 101.11)	68.4	4.48(5.65 – 3.5)	2.75(3.51 – 2.03)	-1.62(-2.6 – -0.63)
Guinea	Male	220.96(314.28 – 149.46)	425.07(639.48 – 275.19)	204.11	13.59(19.22 – 9.16)	15.43(23 – 10.05)	0.42(0.3 – 0.54)
Guinea-Bissau	Male	13.81(21.68 – 7.55)	28.34(43.09 – 18.28)	14.53	7.04(10.9 – 3.9)	8.05(12.18 – 5.29)	0.47(0.28 – 0.67)
Guyana	Male	19.27(24.58 – 15.05)	28.68(40.49 – 19.61)	9.41	10.78(13.82 – 8.43)	9.32(12.98 – 6.35)	-0.3(-1.18 – 0.6)
Haiti	Male	171.97(274.04 – 106.74)	239.93(376.17 – 140.64)	67.96	10.87(17.59 – 6.79)	7.23(11.29 – 4.24)	-1.28(-1.34 – -1.22)

Honduras	Male	39.23(55.95 – 26.98)	150.21(236.17 – 90.2)	110.98	4.15(5.84 – 2.83)	5.32(8.28 – 3.17)	0.83(0.71 – 0.95)
Hungary	Male	5469.02(6375.53 – 4604.54)	5608.97(6992.28 – 4447.62)	139.95	87.64(102.46 – 73.39)	69.64(86.5 – 55.53)	-0.63(-1.25 – -0.01)
Iceland	Male	79.4(93.54 – 65.76)	69.81(89.73 – 55.52)	-9.59	60.34(71.15 – 50.16)	24.78(31.81 – 19.65)	-2.82(-3.26 – -2.39)
India	Male	26534.5(34707.44 – 17518.28)	50872.61(65535.72 – 39036.33)	24338.11	12.05(15.88 – 7.9)	9.43(12.26 – 7.27)	-0.78(-1.17 – -0.38)
Indonesia	Male	5829.53(7752.64 – 4439.57)	15851.41(27944.66 – 9895.55)	10021.88	13.13(17.82 – 9.86)	14.64(26.41 – 9.08)	0.35(0.29 – 0.41)
Iran (Islamic Republic of)	Male	3002.32(3992.53 – 2131.49)	8042.34(9983.1 – 5991.58)	5040.02	21.78(28.97 – 15.43)	20.96(26.14 – 15.57)	-0.11(-0.35 – 0.14)
Iraq	Male	2603.78(3712.29 – 1732.98)	7784.53(11658.2 – 4945.05)	5180.75	70.19(99.57 – 46.65)	74.45(109.66 – 47.33)	0.23(-0.02 – 0.48)
Ireland	Male	1133.96(1309.12 – 972.46)	843.3(1032.85 – 665.93)	-290.66	61.13(70.64 – 52.23)	21.85(26.76 – 17.37)	-3.25(-3.77 – -2.74)
Israel	Male	1338.16(1566.27 – 1120.89)	1953.68(2385.95 – 1547.43)	615.52	59.85(70.22 – 50.18)	34.46(42.08 – 27.54)	-1.62(-2.07 – -1.17)
Italy	Male	48296.57(54421.13 – 41420.14)	32995.71(39675.94 – 26882.98)	-15300.86	124.55(140.73 – 106.93)	50.22(59.95 – 41.24)	-2.96(-3.21 – -2.71)
Jamaica	Male	145.7(177.63 – 118.28)	222.81(313.61 – 151.14)	77.11	17.43(21.15 – 14.22)	15.14(21.28 – 10.31)	-0.44(-2.72 – 1.9)
Japan	Male	26939.7(29886.16 – 24011.67)	35412.21(41899.01 – 28874.63)	8472.51	38(42.32 – 33.65)	21.35(24.86 – 17.84)	-1.83(-2.13 – -1.54)
Jordan	Male	349.7(467.11 – 252.93)	1491.83(2118.45 – 1007.65)	1142.13	54.38(72.69 – 39.77)	39.13(55.55 – 26.87)	-1.05(-1.22 – -0.88)
Kazakhstan	Male	2425.6(3396.44 – 1752.61)	2349.85(2876.63 – 1842.38)	-75.75	48.86(67.51 – 35.49)	30.8(38.5 – 23.95)	-1.4(-1.89 – -0.91)

Kenya	Male	151.81(238.95 – 91.25)	438.99(589.19 – 308.14)	287.18	4.13(6.42 – 2.51)	4.61(6.14 – 3.22)	0.36(0.23 – 0.49)
Kiribati	Male	0.69(0.91 – 0.51)	1.48(2.05 – 1)	0.79	4.11(5.4 – 3.06)	4.43(6.02 – 3.11)	0.24(0.21 – 0.28)
Kuwait	Male	83.27(100.74 – 66.63)	448.97(577.82 – 339.63)	365.7	24.95(30.34 – 19.75)	27.75(35.96 – 20.63)	0.05(-0.95 – 1.06)
Kyrgyzstan	Male	443.75(537.76 – 345.32)	624.04(799.18 – 483.25)	180.29	38.37(46.6 – 29.65)	29.55(37.63 – 22.81)	-0.81(-1.74 – 0.13)
Lao People's Democratic Republic	Male	238.79(385.52 – 135.09)	392.12(597.17 – 248.12)	153.33	25.17(40.52 – 14.43)	19.27(29.82 – 12.17)	-0.86(-0.91 – -0.8)
Latvia	Male	1015.36(1170.07 – 882.44)	1139.57(1398.81 – 906.29)	124.21	77.62(89.44 – 66.71)	78.18(96.1 – 62.33)	0.02(-0.47 – 0.51)
Lebanon	Male	1343.82(1946.63 – 824.93)	3138.5(4333.01 – 2227.38)	1794.68	132.87(191.67 – 81.36)	112.23(154.81 – 79.4)	-0.52(-0.71 – -0.32)
Lesotho	Male	54.29(84.89 – 36.39)	121.78(190.6 – 66.11)	67.49	18.98(29.76 – 12.95)	29.53(45.78 – 16.53)	1.48(1.06 – 1.91)
Liberia	Male	40.73(60.82 – 26.43)	56.13(86.09 – 35.57)	15.4	6.6(9.77 – 4.36)	4.86(7.16 – 3.12)	-1.01(-1.19 – -0.84)
Libya	Male	653.63(954.29 – 437.86)	2033.7(2917.43 – 1401.9)	1380.07	69.24(101.27 – 45.82)	81.49(116.89 – 56.15)	0.52(0.27 – 0.78)
Lithuania	Male	1336.51(1548.11 – 1113.72)	1357.06(1623.7 – 1111.97)	20.55	77.76(89.79 – 64.28)	63.43(75.93 – 51.91)	-1.01(-3.63 – 1.69)
Luxembourg	Male	191.44(226.57 – 159.04)	172.28(211.27 – 135.32)	-19.16	84.51(100.33 – 69.79)	34.72(42.6 – 27.3)	-2.99(-3.67 – -2.31)
Madagascar	Male	214.77(298.87 – 151.02)	209.35(302.49 – 131.04)	-5.42	9.19(12.74 – 6.43)	4.52(6.57 – 2.85)	-2.3(-2.55 – -2.06)
Malawi	Male	619.75(826.23 – 456.28)	1508.42(2134.7 – 981.13)	888.67	40.8(54.01 – 29.89)	54.02(75.89 – 36.95)	0.92(0.77 – 1.07)
Malaysia	Male	1396.86(1959.55 – 809.79)	3604.9(4834.89 – 2411)	2208.04	33.77(47.71 – 19.25)	26.97(36.49 – 17.67)	-0.79(-1.16 – -0.43)

Maldives	Male	9.98(15.05 – 6.42)	21.87(29.34 – 15.93)	11.89	21.96(32.51 – 14.08)	14.13(18.76 – 10.34)	-1.48(-1.75 – -1.21)
Mali	Male	477.34(633.93 – 341.47)	1410.83(2060.12 – 971.89)	933.49	27.29(36.32 – 19.57)	35.7(51.39 – 24.45)	0.91(0.77 – 1.06)
Malta	Male	158.52(186.44 – 132.7)	147.15(185.25 – 117.88)	-11.37	82.5(97.21 – 68.78)	33.31(41.76 – 26.94)	-2.74(-3.33 – -2.15)
Marshall Islands	Male	1.17(1.84 – 0.75)	3.06(4.62 – 1.81)	1.89	14.35(22.23 – 9.09)	16.01(23.83 – 9.74)	0.38(0.26 – 0.49)
Mauritania	Male	48.83(71.17 – 33.9)	69.69(114.99 – 38.33)	20.86	10.47(15.31 – 7.31)	6.47(10.67 – 3.64)	-1.58(-1.75 – -1.4)
Mauritius	Male	175.82(201.23 – 151.37)	216.69(251.61 – 186.34)	40.87	58.19(66.91 – 49.6)	26.18(30.46 – 22.39)	-2.62(-3.45 – -1.77)
Mexico	Male	2756.98(3205.53 – 2343.8)	4181.59(5214.75 – 3275.1)	1424.6 1	14.36(16.74 – 12.19)	7.32(9.11 – 5.73)	-2.27(-2.38 – -2.16)
Micronesia (Federated States of)	Male	4.82(6.97 – 3.12)	8.13(11.74 – 4.88)	3.31	19.63(28.52 – 12.73)	20.85(30.07 – 12.9)	0.19(0.17 – 0.21)
Monaco	Male	20.83(32.7 – 12.18)	23.17(66.54 – 10.21)	2.34	70.32(111.4 – 40.99)	51.12(147.91 – 22.32)	-1.05(-1.1 – -1)
Mongolia	Male	96.1(142.78 – 57.8)	167.46(244.31 – 116.44)	71.36	20.06(29.66 – 12.15)	16.34(23.76 – 11.33)	-0.74(-0.99 – -0.48)
Montenegro	Male	173.83(233.57 – 126.29)	298.61(416.51 – 207.96)	124.78	63.07(85.18 – 45.67)	66.04(91.58 – 45.85)	0.28(0 – 0.55)
Morocco	Male	921.38(1251.69 – 639.86)	1933.75(2727.88 – 1306.76)	1012.3 7	13.18(17.85 – 9)	11.29(16.08 – 7.71)	-0.5(-0.59 – -0.41)
Mozambique	Male	262.68(392.29 – 168.09)	527.61(765.27 – 374.47)	264.93	10.47(15.43 – 6.8)	12.19(17.66 – 8.67)	0.51(0.36 – 0.66)
Myanmar	Male	2190.95(3190.5 – 1532.01)	2337.32(3800.26 – 1579.67)	146.37	21.58(31.21 – 15.19)	12.21(20.5 – 8.25)	-1.83(-1.9 – -1.76)
Namibia	Male	20.31(28.54 – 14.11)	41.31(55.51 – 30.95)	21	8.45(11.79 – 5.88)	8.63(11.2 – 6.57)	0.1(-0.04 – 0.23)
Nauru	Male	0.56(0.85 – 0.31)	0.63(1 – 0.34)	0.07	20.81(30.9 – 11.92)	23.01(35.16 – 12.7)	0.32(0.28 – 0.37)

Nepal	Male	504.37(821.6 – 290.34)	922.95(1949.07 – 567.06)	418.58	12.01(19.4 – 6.77)	9.19(19.31 – 5.68)	-0.84(-0.99 – -0.69)
Netherlands	Male	8406.8(9436.17 – 7227.16)	6493.34(7916.9 – 5407.06)	-1913.46	100.66(113.48 – 86.32)	38.47(46.86 – 32.2)	-3.02(-3.71 – -2.34)
New Zealand	Male	728.65(865.78 – 608.52)	849.41(1067.8 – 656.79)	120.76	41.43(49.07 – 34.47)	21.01(26.28 – 16.33)	-2.13(-2.72 – -1.53)
Nicaragua	Male	26.93(34.95 – 19.34)	76.81(106.13 – 55.69)	49.88	4.16(5.48 – 2.98)	3.66(5.05 – 2.62)	-0.45(-0.81 – -0.09)
Niger	Male	45.49(73.58 – 29.02)	92.2(162.35 – 54.19)	46.71	3.87(6.31 – 2.42)	2.83(4.97 – 1.64)	-1.01(-1.15 – -0.87)
Nigeria	Male	234.9(376.03 – 143.88)	351.35(586.3 – 226.68)	116.45	1.13(1.83 – 0.7)	0.87(1.48 – 0.55)	-0.83(-0.96 – -0.7)
Niue	Male	0.14(0.19 – 0.1)	0.17(0.24 – 0.11)	0.03	15.35(20.52 – 10.25)	16.3(23.25 – 10.45)	0.19(0.12 – 0.25)
North Macedonia	Male	692.63(868.98 – 537.19)	1174.47(1571.86 – 816.15)	481.84	77.32(97.54 – 60.07)	72.64(96.55 – 51.01)	-0.19(-0.45 – 0.07)
Northern Mariana Islands	Male	1.53(2.48 – 1.02)	5.22(6.83 – 3.91)	3.69	14.02(23.38 – 9.63)	18.43(24.05 – 13.73)	0.91(0.58 – 1.25)
Norway	Male	2155.18(2482.87 – 1863.3)	1084.25(1330.26 – 858.56)	-1070.93	71.83(82.76 – 62.43)	22.61(27.6 – 17.94)	-3.64(-3.87 – -3.4)
Oman	Male	42.02(63.18 – 26.54)	78.96(108.55 – 53.74)	36.94	12.73(18.88 – 7.86)	8.69(12.03 – 5.89)	-1.26(-1.89 – -0.62)
Pakistan	Male	15562.08(20507.13 – 11465.94)	26560.34(39419.86 – 18281.95)	10998.26	51.6(67.48 – 37.92)	45.55(67.38 – 31.78)	-0.42(-0.48 – -0.36)
Palau	Male	0.36(0.5 – 0.25)	0.73(1.02 – 0.5)	0.37	7.29(9.94 – 5.17)	5.64(7.86 – 3.95)	-0.88(-1.02 – -0.74)
Palestine	Male	241.17(344.39 – 162.22)	570.84(750.27 – 417.13)	329.67	65.34(92.21 – 44.14)	51.02(68.46 – 37.97)	-0.79(-1.05 – -0.53)
Panama	Male	51.94(62.24 – 43.01)	106.89(143.25 – 77.14)	54.95	7.2(8.62 – 5.95)	5.06(6.79 – 3.65)	-1.05(-1.47 – -0.64)

Papua New Guinea	Male	69.1(114.17 – 32.66)	188(321.34 – 88.62)	118.9	6.91(11.37 – 3.34)	6.25(10.38 – 3.04)	-0.33(-0.51 – -0.16)
Paraguay	Male	160.83(212.82 – 117.65)	410.42(580.4 – 269.97)	249.59	16.56(21.84 – 12.12)	16.02(22.55 – 10.62)	0.01(-0.43 – 0.45)
Peru	Male	311(432.35 – 216.63)	704.09(1088.1 – 434.96)	393.09	5.72(8.05 – 3.92)	4.49(6.96 – 2.77)	-0.67(-1.64 – 0.31)
Philippines	Male	1761.21(2296.43 – 1357.37)	4457.52(6330.08 – 3263.65)	2696.31	12.16(15.88 – 9.25)	11.66(17 – 8.55)	-0.07(-0.14 – 0)
Poland	Male	20109.29(22248.41 – 17842.7)	26021.47(31119.06 – 21238.63)	5912.18	107.9(120.06 – 95.13)	83.45(99.74 – 68.33)	-0.85(-1.01 – -0.68)
Portugal	Male	3431.56(4016.07 – 2885.32)	3866.36(4714.02 – 3077.38)	434.8	57.28(67.05 – 48)	37.55(45.46 – 30.4)	-1.44(-1.74 – -1.14)
Puerto Rico	Male	314.14(399.78 – 243.27)	456.39(617.93 – 336.19)	142.25	18.8(23.91 – 14.49)	14.66(19.77 – 10.74)	-0.81(-1.85 – 0.24)
Qatar	Male	24.82(35.13 – 16.78)	111.3(172.64 – 66.98)	86.48	38.63(53.89 – 27.15)	19.66(30.72 – 12.18)	-2.17(-2.93 – -1.4)
Republic of Korea	Male	6413.16(7890.42 – 4799.95)	10752.9(14067.29 – 7492.08)	4339.74	61.19(76.49 – 45.62)	27.53(36.35 – 18.91)	-2.56(-2.7 – -2.42)
Republic of Moldova	Male	1069(1247.92 – 871.33)	1539.99(1803 – 1278.95)	470.99	57.63(67.56 – 46.76)	60.72(70.77 – 50.6)	0.1(-1.44 – 1.67)
Romania	Male	7997.74(9198.22 – 6738.7)	10741.06(13002.5 – 8670.96)	2743.32	61.07(70.14 – 51.51)	67.56(81.29 – 54.8)	0.27(0.06 – 0.47)
Russian Federation	Male	50609.11(56484.43 – 44867.32)	51848.05(60833.11 – 42950.41)	1238.94	79.57(89.28 – 70.24)	55.5(65.2 – 46.09)	-1.13(-1.71 – -0.54)
Rwanda	Male	258.04(376.46 – 180.44)	399.65(663.17 – 219.22)	141.61	22.43(32.34 – 15.89)	17.92(29.63 – 10.22)	-0.73(-0.84 – -0.62)
Saint Kitts and Nevis	Male	2.42(3.09 – 1.84)	3.3(4.57 – 2.24)	0.88	14.55(18.69 – 11.01)	10.95(15.17 – 7.53)	-0.78(-1.1 – -0.47)
Saint Lucia	Male	9.3(11.4 – 7.48)	17.05(22.6 – 12.54)	7.75	25.28(31.42 – 20.1)	15.03(19.97 – 11.07)	-1.57(-2 – -1.15)

Saint Vincent and the Grenadines	Male	4.44(5.45 – 3.49)	9.28(11.7 – 7.14)	4.84	14.22(17.55 – 11.16)	12.66(15.99 – 9.71)	-0.37(-1.13 – 0.41)
Samoa	Male	8.51(12.56 – 6.14)	12.05(17.5 – 8.08)	3.54	20.99(30.12 – 15.34)	17.21(24.42 – 11.7)	-0.64(-0.68 – -0.6)
San Marino	Male	15.62(21.01 – 11.28)	12.55(19.11 – 7.55)	-3.07	97(130.28 – 70.06)	35.79(55.69 – 20.88)	-3.57(-4.04 – -3.09)
Sao Tome and Principe	Male	2.78(3.85 – 1.93)	6.93(10.39 – 4.36)	4.15	9.77(13.43 – 7.03)	12.82(19.7 – 7.96)	0.91(0.54 – 1.29)
Saudi Arabia	Male	384(602.35 – 231.33)	1387.6(2664.39 – 839.08)	1003.6	11.43(17.77 – 7.02)	10.83(20.06 – 6.65)	-0.19(-0.28 – -0.1)
Senegal	Male	198.61(272.22 – 135.73)	304.49(445.26 – 201.14)	105.88	11.59(15.94 – 7.95)	7.82(11.69 – 5.1)	-1.28(-1.56 – -1)
Serbia	Male	3669.15(4880.71 – 2705.74)	4832.85(6809.99 – 3333.14)	1163.7	73.48(98.02 – 54.08)	63.88(89.42 – 44.25)	-0.41(-0.56 – -0.25)
Seychelles	Male	17.01(21.74 – 12.99)	23.13(30.73 – 17.82)	6.12	71.35(91.09 – 54.44)	45.13(59.27 – 34.66)	-1.55(-2.44 – -0.65)
Sierra Leone	Male	111.22(151.09 – 76.31)	128.33(190.47 – 89.11)	17.11	10.64(14.42 – 7.33)	6.67(9.75 – 4.71)	-1.48(-1.65 – -1.3)
Singapore	Male	225.46(269.64 – 184.87)	332.23(409.24 – 260.8)	106.77	22.87(27.41 – 18.74)	7.94(9.83 – 6.2)	-3.5(-4.73 – -2.24)
Slovakia	Male	2067.2(2721.77 – 1610.3)	2230.57(3085.26 – 1590.25)	163.37	80.6(105.26 – 62.63)	53.78(74.42 – 38.18)	-1.29(-1.58 – -0.99)
Slovenia	Male	563.36(671.36 – 468.91)	747.13(949.91 – 576.52)	183.77	55.82(66.79 – 46.13)	37.83(48.02 – 29.28)	-1.29(-1.55 – -1.04)
Solomon Islands	Male	12.93(20.68 – 5.92)	34.71(55.63 – 18.39)	21.78	16.19(25.73 – 7.68)	18.61(29.5 – 9.92)	0.44(0.31 – 0.56)
Somalia	Male	112.4(200.25 – 63.36)	213.18(371.93 – 103.39)	100.78	10.98(19.58 – 6.1)	9.02(15.6 – 4.56)	-0.62(-0.71 – -0.54)
South Africa	Male	2049.98(2892.15 – 1450.17)	3243.87(3962.84 – 2487.46)	1193.89	23.9(34.31 – 16.86)	16.7(20.59 – 12.81)	-1.19(-1.8 – -0.58)
South Sudan	Male	146.77(228.07 – 93.8)	156.93(257.15 – 96.04)	10.16	10.12(15.21 – 6.43)	8.39(13.35 – 5.21)	-0.6(-0.7 – -0.49)

Spain	Male	32773.63(37170.49 – 28142.36)	27129.4(33173.37 – 21874.3)	-5644.23	136.67(155.22 – 117.24)	62.67(76.12 – 50.81)	-2.52(-2.75 – -2.28)
Sri Lanka	Male	556(744.74 – 416.01)	853.8(1250.74 – 463.03)	297.8	11.25(15.03 – 8.39)	7.47(10.91 – 4.2)	-1.33(-2.03 – -0.62)
Sudan	Male	1347.37(2412.26 – 721.29)	2168.73(3171.13 – 1435.4)	821.36	27.98(50.01 – 15.23)	21.01(30.62 – 14)	-0.93(-0.97 – -0.89)
Suriname	Male	20.76(27.44 – 15.12)	42.66(60.46 – 27.2)	21.9	16.94(22.48 – 12.25)	14.32(20.32 – 9.21)	-0.45(-1.15 – 0.26)
Sweden	Male	2884.34(3361.35 – 2401.95)	2508.4(3166.11 – 1953.77)	-375.94	42.78(49.61 – 35.56)	23.51(29.41 – 18.36)	-1.91(-2.42 – -1.4)
Switzerland	Male	1725.38(2017.26 – 1437.47)	2631.55(3193.95 – 2130.39)	906.17	39.65(46.24 – 33.03)	30.57(36.85 – 24.77)	-0.95(-1.92 – 0.03)
Syrian Arab Republic	Male	931.52(1269.56 – 660.57)	2045.46(2956.66 – 1337.27)	1113.94	34.49(47.11 – 24.63)	29.87(42.92 – 19.66)	-0.53(-0.94 – -0.13)
Taiwan (Province of China)	Male	4222.83(4813.54 – 3626.14)	7279.8(8490.32 – 6050.19)	3056.97	52.32(59.82 – 44.48)	37.04(43.01 – 30.81)	-0.99(-1.3 – -0.67)
Tajikistan	Male	196.4(300.46 – 127.21)	214.6(325.55 – 132.05)	18.2	16.47(25.62 – 10.78)	7.43(11.24 – 4.72)	-2.58(-2.99 – -2.17)
Thailand	Male	6379.33(8329.63 – 4487.25)	12622.14(17932.27 – 8887.55)	6242.81	43.44(56.34 – 30.2)	25.58(36.27 – 18.08)	-1.72(-1.98 – -1.46)
Timor-Leste	Male	13.71(22.72 – 7.88)	41.73(65.97 – 26.59)	28.02	10.84(17.61 – 6.21)	10.25(16.26 – 6.55)	-0.17(-0.38 – 0.04)
Togo	Male	101.52(138.61 – 72)	224.92(328.24 – 152.06)	123.4	18.54(25.09 – 13.25)	14.22(20.31 – 9.63)	-0.86(-0.96 – -0.75)
Tokelau	Male	0.1(0.15 – 0.07)	0.1(0.14 – 0.06)	0	15.4(22.73 – 9.53)	13.32(19.18 – 8.38)	-0.47(-0.51 – -0.42)
Tonga	Male	4.83(7.9 – 2.89)	7.12(11.78 – 4.2)	2.29	18.63(30.8 – 11.17)	19.36(32.07 – 11.56)	0.1(-0.16 – 0.36)
Trinidad and Tobago	Male	67.06(78.75 – 54.69)	119.82(159.97 – 82.36)	52.76	17.13(20.24 – 13.99)	12.51(16.66 – 8.53)	-0.64(-0.85 – -0.43)

Tunisia	Male	1376.49(1920.76 – 959.28)	3486.22(5112.28 – 2172.81)	2109.73	57.26(78.59 – 40.07)	56.34(81.94 – 35.12)	-0.09(-0.19 – 0.01)
Turkey	Male	15057.99(20755.2 – 10470.37)	27382.01(37324.58 – 19610.6)	12324.02	92.9(127.45 – 65.37)	62.11(84.65 – 44.51)	-1.29(-1.63 – -0.94)
Turkmenistan	Male	203.12(242.52 – 171.38)	290.22(398.11 – 205.74)	87.1	24.61(29.68 – 20.58)	15.24(20.83 – 10.85)	-1.64(-2.48 – -0.79)
Tuvalu	Male	0.54(0.77 – 0.35)	0.85(1.23 – 0.55)	0.31	17.5(24.81 – 11.37)	16.77(24.19 – 10.9)	-0.14(-0.17 – -0.1)
Uganda	Male	242.04(342.14 – 163.69)	443.16(650.7 – 308.6)	201.12	8.23(11.45 – 5.67)	7.49(10.87 – 5.27)	-0.36(-0.52 – -0.19)
Ukraine	Male	18379.87(24697.72 – 14202.55)	16651.84(23634.49 – 10754.35)	-1728.03	70.76(93.8 – 54.87)	54.84(77.82 – 35.53)	-0.76(-1.27 – -0.25)
United Arab Emirates	Male	120.75(237.95 – 64.63)	565.93(797.58 – 394.46)	445.18	43.59(87.73 – 22.53)	19.66(27.73 – 13.55)	-2.53(-3.29 – -1.77)
United Kingdom	Male	37900.62(42872.97 – 33244.02)	20333.22(24559.63 – 16261.4)	-17567.4	99.76(112.94 – 87.45)	32.8(39.48 – 26.48)	-3.53(-3.7 – -3.37)
United Republic of Tanzania	Male	751.79(1126.79 – 520.04)	1072.98(1655.33 – 674.31)	321.19	14.96(22.06 – 10.44)	9.29(13.92 – 6)	-1.54(-1.69 – -1.39)
United States of America	Male	62134.34(71413.33 – 53307.8)	82872.63(100983.37 – 67965.42)	20738.29	45.94(52.88 – 39.34)	30.59(37.29 – 25.07)	-1.31(-1.51 – -1.1)
United States Virgin Islands	Male	4.37(6.23 – 2.99)	5.68(8.73 – 3.42)	1.31	11.36(16.15 – 7.68)	6.55(10.08 – 3.97)	-1.7(-2.39 – -1)
Uruguay	Male	1415.5(1652.79 – 1197.71)	1357.7(1640.97 – 1095.97)	-57.8	80.84(94.43 – 68.32)	58.65(70.83 – 47.37)	-1.13(-1.73 – -0.54)
Uzbekistan	Male	474.25(655.31 – 324.5)	1579.54(2088.95 – 1129.16)	1105.29	9.68(13.43 – 6.53)	12.92(17.18 – 9.34)	0.75(0.11 – 1.4)
Vanuatu	Male	4.11(6.36 – 2.35)	9.47(14.79 – 5.72)	5.36	11.8(17.97 – 6.77)	10.63(16.45 – 6.3)	-0.38(-0.6 – -0.16)

Venezuela (Bolivarian Republic of)	Male	651.2(765.32 – 537.3)	1332.96(1861.47 – 937.42)	681.76	15.04(17.7 – 12.29)	10.02(13.99 – 7.04)	-1.42(-2.56 – -0.26)
Viet Nam	Male	2950.41(3999.56 – 1996.68)	8195.4(10591.06 – 6047.01)	5244.99	18.69(24.83 – 12.7)	20.23(25.82 – 15.3)	0.27(0.21 – 0.33)
Yemen	Male	895.94(1469.88 – 477.48)	2387.18(3411.63 – 1557.26)	1491.24	38(61.23 – 20.45)	35.36(49.75 – 23.1)	-0.23(-0.3 – -0.15)
Zambia	Male	130.78(179.14 – 94.39)	343.12(727.13 – 178.34)	212.34	9.21(12.66 – 6.72)	10.89(21.7 – 5.84)	0.56(0.39 – 0.73)
Zimbabwe	Male	982.15(1342.57 – 699.46)	1829.41(2498.86 – 1337.28)	847.26	53.18(71.65 – 37.8)	65.62(88.63 – 48.47)	0.71(0.53 – 0.9)

Supplementary Table 5. Age distribution of death rate (per 100,000) for bladder cancer attributable to smoking in different region by sex in 2021.

	Female						Male					
Age group	30-49		50-69		70+		30-49		50-69		70+	
Location	Death cases No. *103	Death rate per 100,000	Death cases No. *103	Death rate per 100,000	Death cases No. *103	Death rate per 100,000	Death cases No. *103	Death rate per 100,000	Death cases No. *103	Death rate per 100,000	Death cases No. *103	Death rate per 100,000
Global	98.58	0.01	1555.06	0.21	4023.09	1.45	1129.40	0.10	17310.36	2.48	34650.38	17.40

High SDI	45.67	0.03	798.52	0.53	2512.08	2.83	167.21	0.10	4125.87	2.84	11535.97	18.97
High middle SDI	28.03	0.01	382.62	0.22	850.12	1.23	382.42	0.18	6290.36	3.95	11716.17	26.01
Middle SDI	14.65	＜0.01	226.98	0.09	434.53	0.59	377.76	0.10	4436.41	1.92	8132.31	14.46
Low middle SDI	6.47	＜0.01	103.11	0.08	162.34	0.46	153.74	0.07	2001.64	1.67	2675.29	9.05
Low SDI	3.55	＜0.01	40.05	0.09	58.06	0.57	47.16	0.05	429.80	1.01	548.79	5.95
Andean Latin America	0.27	＜0.01	2.98	0.06	5.62	0.32	1.71	0.02	21.60	0.47	55.77	3.79
Australasia	1.07	0.02	11.44	0.29	46.56	2.22	2.85	0.06	41.49	1.13	131.99	8.02
Caribbean	0.81	0.01	14.49	0.32	29.49	1.63	4.08	0.06	92.70	2.25	176.44	12.42
Central Asia	0.49	＜0.01	3.18	0.04	2.19	0.10	20.85	0.17	239.50	3.38	214.84	17.44
Central Europe	9.79	0.05	190.91	1.09	247.10	2.68	40.27	0.20	1238.59	7.77	1760.91	31.66
Central Latin America	1.95	0.01	19.67	0.09	30.72	0.41	9.19	0.03	121.08	0.62	242.26	4.12
Central Sub-Saharan Africa	0.44	＜0.01	2.78	0.05	1.53	0.15	6.34	0.05	54.02	1.14	34.68	5.17
East Asia	6.82	＜0.01	177.60	0.09	589.53	0.93	454.29	0.19	5199.29	2.68	11287.61	24.73
Eastern Europe	6.49	0.02	40.74	0.12	36.12	0.24	72.59	0.23	1571.01	6.30	1539.60	23.70
Eastern Sub-Saharan Africa	1.46	＜0.01	17.63	0.12	24.60	0.73	13.33	0.04	119.46	0.86	135.82	4.88
High-income Asia Pacific	2.38	0.01	33.27	0.12	282.90	1.08	19.35	0.06	413.75	1.56	2286.64	15.18

High-income North America	19.89	0.04	339.13	0.65	941.33	3.73	45.03	0.09	1291.46	2.65	2917.32	16.07
North Africa and Middle East	5.76	0.01	43.79	0.11	68.85	0.75	139.73	0.15	1572.82	3.84	1988.70	21.12
Oceania	0.24	0.01	1.61	0.24	0.49	0.34	1.34	0.08	7.05	0.97	3.79	2.98
South Asia	3.14	＜0.01	72.84	0.06	128.91	0.36	97.21	0.04	1510.72	1.20	2377.53	7.79
Southeast Asia	1.79	＜0.01	25.07	0.04	67.16	0.42	80.58	0.08	889.97	1.55	1211.44	10.76
Southern Latin America	2.64	0.03	36.73	0.52	46.80	1.43	9.02	0.09	180.97	2.88	242.03	10.97
Southern Sub-Saharan Africa	2.00	0.02	14.55	0.25	20.73	1.32	13.71	0.14	106.55	2.42	88.48	10.17
Tropical Latin America	7.16	0.02	115.28	0.50	152.98	1.83	13.55	0.04	339.81	1.72	629.56	10.95
Western Europe	23.60	0.04	386.52	0.63	1294.66	3.07	69.09	0.10	2207.44	3.73	7218.64	25.18
Western Sub-Saharan Africa	0.39	＜0.01	4.85	0.03	4.83	0.13	15.27	0.04	91.08	0.58	106.34	3.04

Supplementary Table 6. Age distribution of DALY rate (per 100,000) for bladder cancer attributable to smoking in different region by sex in 2021.

	Female						Male					
Age group	30-49		50-69		70+		30-49		50-69		70+	
Location	DALYs number	DALY rate per 100,000	DALYs number	DALY rate per 100,000	DALYs number	DALY rate per 100,000	DALYs number	DALY rate per 100,000	DALYs number	DALY rate per 100,000	DALYs number	DALY rate per 100,000
Global	4903.05	0.46	48620.88	6.59	58363.45	21.07	55794.64	5.11	533117.01	76.39	537504.12	255.33
High SDI	2331.85	1.47	25393.54	17.15	35966.80	43.08	8516.06	5.06	128918.30	89.95	173939.34	282.82
High middle SDI	1384.31	0.66	11961.14	6.96	12419.04	18.10	19018.43	8.92	194159.02	122.18	184491.67	387.56
Middle SDI	702.53	0.19	6888.75	2.83	6474.06	8.51	18594.12	5.07	136099.76	58.70	126542.38	208.39
Low middle SDI	306.80	0.13	3073.02	2.41	2502.37	6.75	7372.88	3.11	60295.50	49.95	42974.50	134.99
Low SDI	167.31	0.16	1188.78	2.69	911.64	8.25	2239.65	2.16	12855.98	29.66	8884.18	87.33
Andean Latin America	13.05	0.15	91.41	1.84	81.49	4.64	83.69	0.97	647.79	13.99	828.34	55.06
Australasia	54.44	1.22	362.12	9.47	648.76	32.16	142.84	3.27	1318.31	36.53	2005.00	120.18

Caribbean	38.65	0.59	448.57	9.93	420.51	23.69	194.05	3.04	2813.06	67.87	2766.73	192.24
Central Asia	24.10	0.18	99.89	1.16	35.95	1.69	1002.38	8.01	7333.20	102.02	3481.05	268.50
Central Europe	476.88	2.52	5796.00	34.13	3868.23	42.63	1941.55	9.84	36688.54	234.96	28904.08	500.20
Central Latin America	93.34	0.25	599.80	2.67	460.28	6.09	444.03	1.32	3643.58	18.65	3664.50	60.85
Central Sub-Saharan Africa	20.80	0.17	87.07	1.56	24.70	2.19	298.52	2.42	1648.89	33.76	586.13	78.59
East Asia	334.60	0.15	5287.35	2.67	8681.43	13.22	22677.44	9.53	160558.66	82.69	173902.70	341.69
Eastern Europe	326.31	0.96	1282.00	4.00	541.99	3.60	3505.11	10.88	47288.02	191.43	25984.06	384.45
Eastern Sub-Saharan Africa	69.10	0.18	519.06	3.45	380.78	10.46	625.49	1.69	3588.39	25.21	2196.91	72.18
High-income Asia Pacific	121.66	0.42	1031.22	3.83	3454.32	14.74	1007.35	3.28	13123.43	50.28	32396.01	217.64
High-income North America	1061.90	2.11	11229.52	22.02	14465.39	59.00	2392.57	4.83	42113.61	87.65	46753.83	250.14
North Africa and Middle East	276.41	0.34	1370.68	3.45	1019.74	10.37	6982.90	7.44	50099.73	120.56	32664.00	329.86
Oceania	11.67	0.72	51.75	7.54	8.76	5.85	67.44	4.10	220.20	29.67	63.36	45.22
South Asia	148.06	0.06	2131.41	1.67	2002.15	5.30	4575.88	1.89	44531.17	35.33	37561.14	112.47
Southeast Asia	86.02	0.08	747.07	1.18	959.78	5.81	3938.20	3.83	27181.75	46.91	18829.31	154.75

Southern Latin America	127.18	1.27	1128.94	16.12	750.02	23.39	432.02	4.49	5482.17	87.49	3947.67	174.35
Southern Sub-Saharan Africa	95.31	0.92	447.07	7.71	318.03	19.28	653.26	6.51	3272.63	73.44	1442.70	149.80
Tropical Latin America	341.17	0.96	3519.11	15.28	2306.60	27.76	648.59	1.94	10038.28	50.46	9660.40	161.81
Western Europe	1163.83	1.82	12240.87	20.09	17860.82	45.96	3440.12	5.27	68824.44	117.30	108196.10	380.57
Western Sub-Saharan Africa	18.57	0.04	149.99	0.80	73.72	1.79	741.21	1.77	2701.17	16.84	1670.09	44.43

Supplementary Table 7. Prediction the burden of bladder cancer attributable to smoking

	Prediction of bladder cancer death cases attributable to smoking		
year	Female cases	Male cases	Both cases
2022	5687.94	53562.00	59997.82
2023	5715.50	54619.72	61023.26
2024	5766.17	56062.33	62282.20
2025	5818.43	57448.43	63553.75
2026	5873.20	58729.20	64816.60
2027	5932.46	60004.60	66106.69
2028	5996.68	61327.82	67458.56
2029	6057.46	62622.70	68782.81
2030	6114.49	63885.33	70071.03
2031	6172.71	65129.85	71338.88
2032	6237.47	66416.05	72648.82
2033	6308.92	67778.49	74039.50
2034	6374.89	69105.02	75389.65

2035	6432.88	70377.33	76676.49
2036	6488.12	71612.62	77920.01
2037	6548.47	72876.44	79191.38
2038	6614.99	74219.55	80544.29
2039	6673.81	75527.56	81853.18
2040	6722.01	76778.92	83092.44
2041	6763.21	77974.51	84265.37
2042	6806.40	79166.19	85432.28
2043	6855.31	80420.81	86663.53
2044	6894.46	81635.90	87841.54
2045	6921.71	82803.85	88956.10
2046	6939.43	83939.66	90021.45

© 2025 Qi K. et al.
